# Biotic and Human Vulnerability to Projected Changes in Ocean Biogeochemistry over the 21st Century

**DOI:** 10.1371/journal.pbio.1001682

**Published:** 2013-10-15

**Authors:** Camilo Mora, Chih-Lin Wei, Audrey Rollo, Teresa Amaro, Amy R. Baco, David Billett, Laurent Bopp, Qi Chen, Mark Collier, Roberto Danovaro, Andrew J. Gooday, Benjamin M. Grupe, Paul R. Halloran, Jeroen Ingels, Daniel O. B. Jones, Lisa A. Levin, Hideyuki Nakano, Karl Norling, Eva Ramirez-Llodra, Michael Rex, Henry A. Ruhl, Craig R. Smith, Andrew K. Sweetman, Andrew R. Thurber, Jerry F. Tjiputra, Paolo Usseglio, Les Watling, Tongwen Wu, Moriaki Yasuhara

**Affiliations:** 1 Department of Geography, University of Hawaii, Honolulu, Hawaii, United States of America; 2 Ocean Science Centre, Memorial University of Newfoundland, St. John's, Newfoundland, Canada; 3 Pacific Islands Fisheries Science Center, Honolulu, Hawaii, United States of America; 4 Norwegian Institute for Water Research, Bergen, Norway; 5 Florida State University, Tallahassee, Florida, United States of America; 6 National Oceanography Centre, University of Southampton Waterfront Campus, Southampton, United Kingdom; 7 Institut Pierre Simon Laplace/Laboratoire des Sciences du Climat et de l'Environnement, Centre National de la Recherche Scientifique, Gif sur Yvette, France; 8 The Centre for Australian Weather and Climate Research, Commonwealth Scientific and Industrial Research Organisation Marine and Atmospheric Research, Aspendale, Victoria, Australia; 9 Department of Life and Environmental Sciences, Polytechnic University of Marche, Ancona, Italy; 10 Center for Marine Biodiversity and Conservation, Scripps Institution of Oceanography, La Jolla, California, United States of America; 11 Met Office Hadley Centre, Exeter, United Kingdom; 12 College of Life and Environmental Sciences, University of Exeter, Exeter, United Kingdom; 13 Marine Biology Research Group, Biology Department, Ghent University, Ghent, Belgium; 14 Plymouth Marine Laboratory, Plymouth, United Kingdom; 15 Meteorological Research Institute, Tsukuba, Japan; 16 Norwegian Institute for Water Research, Oslo, Norway; 17 Institut de Ciències Marines, Consejo Superior de Investigaciones Científicas, Barcelona, Spain; 18 Department of Biology, University of Massachusetts, Boston, Massachusetts, United States of America; 19 Department of Oceanography, University of Hawaii at Manoa, Hawaii, United States of America; 20 International Research Institute of Stavanger, Thormøhlensgate, Bergen, Norway; 21 College of Earth, Ocean, and Atmospheric Sciences, Oregon State University, Corvallis, Oregon, United States of America; 22 Uni Climate, Uni Research, Bergen, Norway; 23 Department of Biology, University of Hawaii at Manoa, Hawaii, United States of America; 24 Centro de innovacion Fundacion In-nova Castilla La Mancha, Madrid, Spain; 25 Beijing Climate Center, China Meteorological Administration, Beijing, China; 26 School of Biological Sciences, Swire Institute of Marine Science, and Department of Earth Sciences, University of Hong Kong, Hong Kong, China; University College London, United Kingdom

## Abstract

Ongoing greenhouse gas emissions can modify climate processes and induce shifts in ocean temperature, pH, oxygen concentration, and productivity, which in turn could alter biological and social systems. Here, we provide a synoptic global assessment of the simultaneous changes in future ocean biogeochemical variables over marine biota and their broader implications for people. We analyzed modern Earth System Models forced by greenhouse gas concentration pathways until 2100 and showed that the entire world's ocean surface will be simultaneously impacted by varying intensities of ocean warming, acidification, oxygen depletion, or shortfalls in productivity. In contrast, only a small fraction of the world's ocean surface, mostly in polar regions, will experience increased oxygenation and productivity, while almost nowhere will there be ocean cooling or pH elevation. We compiled the global distribution of 32 marine habitats and biodiversity hotspots and found that they would all experience simultaneous exposure to changes in multiple biogeochemical variables. This superposition highlights the high risk for synergistic ecosystem responses, the suite of physiological adaptations needed to cope with future climate change, and the potential for reorganization of global biodiversity patterns. If co-occurring biogeochemical changes influence the delivery of ocean goods and services, then they could also have a considerable effect on human welfare. Approximately 470 to 870 million of the poorest people in the world rely heavily on the ocean for food, jobs, and revenues and live in countries that will be most affected by simultaneous changes in ocean biogeochemistry. These results highlight the high risk of degradation of marine ecosystems and associated human hardship expected in a future following current trends in anthropogenic greenhouse gas emissions.

## Abstract 2

Mora and colleagues show that ongoing greenhouse gas emissions are likely to have a considerable effect on several biogeochemical properties of the world's oceans, with potentially serious consequences for biodiversity and human welfare.

## Author Summary

Climate change caused by human activity could damage biological and social systems. Here we gathered climate, biological, and socioeconomic data to describe some of the events by which ocean biogeochemical changes triggered by ongoing greenhouse gas emissions could cascade through marine habitats and organisms, eventually influencing humans. Our results suggest that the entire world's ocean surface will be simultaneously impacted by varying intensities of ocean warming, acidification, oxygen depletion, or shortfalls in productivity. Only a very small fraction of the oceans, mostly in polar regions, will face the opposing effects of increases in oxygen or productivity, and almost nowhere will there be cooling or pH increase. The biological responses to such biogeochemical changes could be considerable since marine habitats and hotspots for several marine taxa will be simultaneously exposed to biogeochemical changes known to be deleterious. The social ramifications are also likely to be massive and challenging as some 470 to 870 million people – who can least afford dramatic changes to their livelihoods – live in areas where ocean goods and services could be compromised by substantial changes in ocean biogeochemistry. These results underline the need for urgent mitigation of greenhouse gas emissions if degradation of marine ecosystems and associated human hardship are to be prevented.

## Introduction

As CO**_2_** and other greenhouse gas emissions continue to rise, ocean biogeochemistry is being altered in ways that could potentially impact nature and mankind. Atmospheric CO**_2_** concentrations have already risen to ∼400 ppm from ∼280 ppm in pre-industrial times and could rise to between 550 and 900 ppm by 2100, depending upon the emission scenario [1]–[7]. In the marine realm, the surplus of CO**_2_** has been associated with ocean warming from the greenhouse effect [1] and acidification caused by the fact that approximately 25% of the annually emitted CO_2_ enters the ocean, where it reacts with water to produce carbonic acid, thereby reducing pH [6]–[8]. Ocean warming and other climatic changes can trigger additional responses in connection to ocean circulation and stratification, which in turn reduce oxygen concentration [9],[10] and primary productivity [11] (additional responses may include sea-level rise and extreme weather events, which we do not analyze here but that certainly will add to the stress likely to be exerted by greenhouse gas emissions [10]). Several analyses predict that, by the year 2100, depending on the emission scenario, surface ocean temperature could increase by 2 to 3°C [9], pH decline by over 0.2 units [6],[7], oxygen concentration decrease by 2% to 4% [9], and ocean productivity by 2% to 20% [11], from current values. The magnitude of these changes would be unprecedented in the Earth's history during the last 20 million years [12],[13].

Species are adapted to their environment, and therefore shifts in environmental parameters can induce considerable change in species fitness and trigger additional responses in community composition, functioning, and overall biodiversity [2],[3],[9]–[11],[14]–[16]. Ocean warming, acidification, oxygen depletion, and reduction in primary production have all been highlighted as potentially having negative biological consequences [2],[3],[9]–[11],[14]–[16]. Changes in temperature, for instance, can affect metabolism, reproduction, and survival [10],[17], which is already evident in multiple shallow and deep-sea ecosystems [2],[18]. Parameters related to food supply, such as primary productivity and sinking organic-carbon flux, and dissolved oxygen can influence metabolism, body size, reproduction, and thus control, in part, the biomass that can be sustained in any given area of the ocean [19]. Moreover, depending on the magnitude of shifts in biogeochemical parameters and/or their proximity to physiological thresholds, these changes can make entire areas essentially unsuitable for metazoans (except for some meiofaunal organisms, as well as viruses, prokaryotes, and certain protists [20]–[23]). There is already evidence that oxygen minimum zones have increased in vertical extent over recent decades, with important consequences for ecosystems and coastal communities [24]. Likewise, pH can influence rates of calcification and several other physiological processes [10],[15],[25],[26]. Co-occurring changes in biogeochemical parameters could also accelerate biological responses, either additively or synergistically [10],[27]–[30]. Warming, for instance, can increase metabolism but, if combined with a reduction in dissolved oxygen and food availability, it could also lead to considerable reductions in body size [31], survival, and synergistic responses of ecosystems [32] and cause range expansions or contractions [2],[10],[17]. Studies on marine invertebrates have also revealed that embryos that survive exposure to warming may later die as larvae if exposed to acidification [33]. This is not to say that all species will be impacted negatively. Some species may expand to new areas or thrive in areas where they were once rare. It is certain, however, that biogeochemical changes in the ocean, especially their co-occurrence, have considerable potential to reorganize patterns in biodiversity, body size, and abundance (Table 1). Additionally, the number of species within ecosystems, variations in life histories, and susceptibility to climate change among species suggest that ecosystem responses to ocean biogeochemistry change are likely to be varied and highly idiosyncratic (Table 1).

**Table 1:** Likely biological responses to changes in ocean biogeochemistry.

	Temperature	pH	Oxygen	Productivity
**Body size and growth**	Due to temperature control over metabolism [60], everything else being equal, warming should reduce growth and body size [31],[61],[62]. In some regions, warming of extreme cold places could enhance individual body growth [63].	Acidification may reduce skeletogenesis [33],[64] and increase metabolic costs of calcification [32], although some taxa are resistant [65] and some plants may benefit [66] (but see [67]). CO_2_ can increase in the blood (i.e., hypercapnia) reducing growth [33],[68]–[70].	Hypoxia (reduced oxygen) should reduce growth and body size [71]–[73]. Oxygen concentration also exerts a strong control over calcification rates of corals [74].	Growth and body size should decline with lowered productivity [19],[31],[75]–[79]. Changes in life-history strategies of abyssal macrofauna may be related to changes in surface productivity [80].
**Survival and abundance**	In some taxa, thermal tolerance thresholds could be surpassed by warming leading to excessive mortality [3],[81]–[83], especially if in interacting with other stressors [29],[84]. Warming thus reduces abundance [83],[85]–[87] and may enhance diseases [88]–[93].	Acidification increases mortality in selected adult [94] and juvenile [95]–[98] marine invertebrates [33] and plants [67]. Abundance can decline among producer species [67] (but see [66],[99]).	Hypoxia causes mortality in most large eukaryote species [23],[71],[84],[100], and anoxia (complete lack of oxygen) could cause extinction in macro- and megafauna [71],[101]–[104]. Hypoxia may enhance dominance by some taxa that are hypoxia tolerant [103],[105],[106] or that are released from ecological interactions [16],[71],[107],[108].	Mortality of benthic invertebrates is generally higher with reductions in food supply [83]. Reduced productivity could reduce abundance [75],[83],[108]–[114] and lead to dominance shifts from large to small taxa [115].
**Range and distribution**	Warming could cause range shifts poleward and to deeper waters [116]–[119], which in turn could affect the strength of ecological interactions [120], gene flow, and rates of evolution [121]. Warming also reduces habitat suitability for species that do not shift ranges [122].	Reduced calcium carbonate saturation could prevent calcification and growth and thus lead to the disappearance of calcifying species from certain shallow [3],[123] and deep-sea [124] areas.	Some taxa may disappear from hypoxic waters [24],[71],[103],[125]–[129] but others may appear and thrive [24],[125],[128]. Some evidence exists for increased endemism among benthic foraminifera in core regions of oxygen minimum zones [130].	Certain species are unlikely to maintain their distribution in food-limited areas of the seafloor [131].
**Species richness**	Theory suggests a positive relation between richness and temperature [132]–[135], which is confirmed in several marine studies [54],[117],[136],[137]; although some regions and/or taxa fail to show a relationship [138].	Acidification will likely lead to loss of species [94],[139],[140].	Diversity declines as oxygen declines for protists [16],[23],[101], meiofauna [16], macrofauna, and megafauna [23],[24],[71],[101],[125].	Richness shows a unimodal [83],[112],[114],[131] or no [137],[138] relationship with proxies of food supply. Productivity seasonality may negatively affect diversity [141],[142]. Eutrophication causes diversity decline via hypoxia and anoxia [16].
**Functioning**	Ecosystem malfunctioning could be extensive if key-stone species are affected [3],[55],[56],[120],[122]. Trophic cascades (e.g., rise of jellyfish) could also occur [105].	Acidification can affect nutrient cycling [140],[143], while reduced calcification can reduce sinking rates and carbon export fluxes to the seafloor via less mineral ballast [144].	Carbon cycling could shift from metazoans to benthic foraminifera [145] and microbiota [20],[145] in suboxic and anoxic zones. Hypoxia can reduce colonization, recovery, and resilience [146].	Reduced food supply can reduce carbon cycling [19],[147],[148], modify food-web structures [114], and cause shifts from macrofaunal- to microbial-dominated nutrient cycling [75],[149],[150].

Socioeconomic systems can also be sensitive to ocean biogeochemical changes, depending upon the exposure of ocean goods and services to environmental change, human dependence on affected services, and social adaptive capacity [34]–[38]. Examples of the goods and services likely to be impacted by ocean climate change are diverse. Ocean warming and acidification, for instance, are causing a new set of conditions that are very close to the tolerance thresholds of corals, making them vulnerable to massive bleaching and mortality when long-term trends related to climate change are “added” to natural variability. The decay of coral reefs could potentially impair their ability to deliver goods and services such as fisheries, tourism, coastal protection, and in some cases aesthetic and spiritual values [35],[37], which have been grossly valued at over US$375 billion annually [39]. Likewise, future changes in ocean temperature are expected to cause a redistribution in the global diversity of cetaceans [40], which in turn could impact local economies that rely on tourism or the fishing of these species. A similar example is the effect of ocean climate change on the world's fisheries, where a combination of warming, oxygen depletion, and reduction in primary productivity can induce changes in body size [31], abundance, and distribution of exploited species [41],[42]. These would add to the ongoing decline of fisheries yields, which are considerable sources of food, revenues, and jobs [36],[42],[43]. Shifts in the distribution and abundance of species could also bring new opportunities for local communities, although adaptability (e.g., flexibility and responsiveness) will be needed to realize any potential benefits [38]. However, the vulnerability of societies to the changes in ocean goods and services ultimately depends on the balance among exposure to environmental change, human dependency on impacted goods and services, and social adaptability [34],[35],[44],[45]. In that context, we are aware of two relevant studies analyzing social vulnerability to ocean climate change over large spatial scales: one for fisheries [34] and the other for coral reefs [35]. Given the limited availability of ocean climate projections at the time, the former study used projected mean surface air temperature to 2050 as the underlying indicator of exposure to climate change, while the latter study focused on five countries of the Western Indian Ocean and used thermal stress on coral reefs as a proxy of climate change. As far as we are aware, more detailed studies connecting the exposure to several and co-occurring stressors of climate change with a variety of ocean goods and services at the global scale are lacking.

As indicated above, we have a relatively good understanding of the potential changes in ocean biogeochemical parameters expected under different greenhouse gas scenarios [7],[9],[11],[46], and conceptually we know some of the mechanisms through which ecological and social systems may be impacted by such changes. However, we lack a synthetic global quantification of the simultaneous projection of biogeochemical changes on the ocean and how they may pertain to marine biota and people worldwide. To address this gap, we compiled all available data generated by Earth Systems Models as part of the Coupled Model Intercomparison Project Phase 5 (CMIP5) to the Fifth Assessment Report of the Intergovernmental Panel on Climate Change [47] to assess the extent of co-occurrence of changes in temperature, pH, oxygen, and primary productivity. We complemented the analysis by assembling global distribution maps of 32 marine habitats and biodiversity hotspots to assess the potential vulnerability of biological systems to co-occurring biogeochemical changes in the ocean. Finally, we used available data on human dependency on ocean goods and services and social adaptability to quantify the vulnerability of coastal people to projected ocean biogeochemical change. We would like to emphasize that our results primarily concern the vulnerability of biological and social systems resulting from their exposure to projected anthropogenic ocean biogeochemical change, while cautioning that, although biotic and social responses will certainly occur, the type and magnitude of such responses will be difficult to predict.

## Results and Discussion

Important contributions have been made to understand future projections in the ocean biogeochemical parameters analyzed here [9],. We repeated the collection of such projections for the purposes of identifying patterns of co-occurrence in biogeochemical variables and to quantify sources of error due to model accuracy and precision. Earth Systems Models in the CMIP5 improve upon earlier models and runs by incorporating better knowledge of the climate, improved computational capability, and CO**_2_** pathways that use more detailed and up-to-date data and integrate multiple forcing agents of climate change [48]. Additionally, as noted below, multimodel averages were always more accurate than individual models, further justifying the assembly of biogeochemical projections based on all available Earth System Models. Although these data on ocean biogeochemical parameters represent an important component of our study, our main goals are to identify how their patterns of co-occurrence may pertain to marine biota and thereby social systems worldwide that rely on marine biodiversity goods and services.

### Earth System Models Precision and Accuracy

The reliability of climate change projections is primarily determined by the skilfulness with which climate models are able to predict the climate [49],[50]. Climate model realism has improved over recent years owing to increased computing power, better scientific understanding of Earth System processes, and the ability to integrate atmosphere, ocean, land, and sea-ice components of the climate system [49]–[51]. However, our theoretical understanding of the climate system is still incomplete and a myriad of unresolved differences exist among models (e.g., spatial and temporal resolution, numerical solution techniques, process parameterizations, and complexity of atmospheric convection, carbon cycle coupling, ocean mixing, unresolved attributes of the biosphere, etc. [49]). As a result, one of the major motivations in climate research has been to quantify the agreement among models as well as between models and actual climate observations. To address these standing concerns, we measured two proxies for model precision and accuracy. Accuracy was defined as the proximity of the model projections to actual data and precision as the standard deviation among the projections of all models. Of course, the availability of actual observations is restricted to recent times and so we assume that a model that accurately simulates present climate will produce better projections of future climates [51].

**Figure 1:**
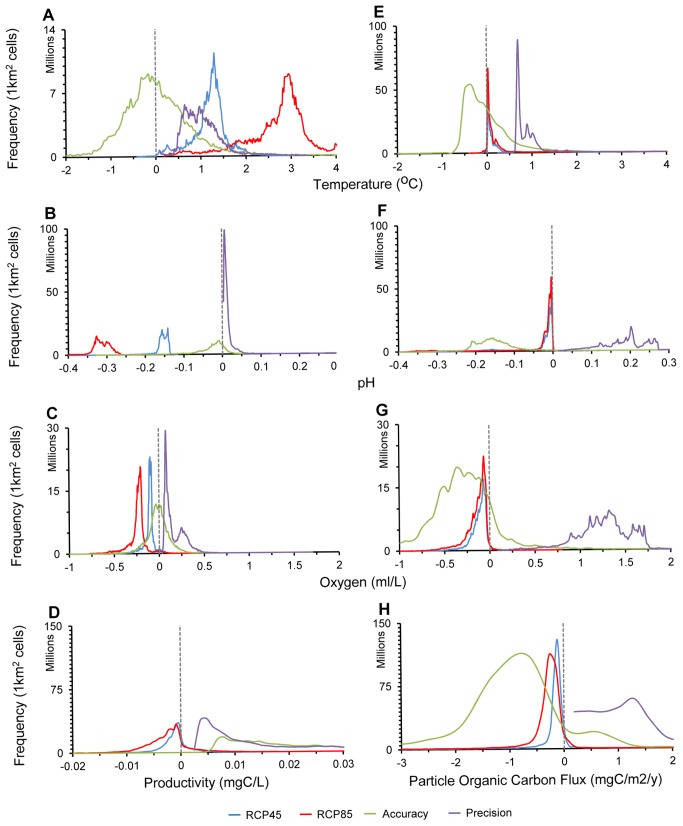
Accuracy and precision on future ocean biogeochemical projections. Plots A–D refer to sea-surface parameters; plots E–H to seafloor parameters. These plots illustrate the number of 1 km^2^ cells by their projected change to the year 2100 under the RCP45 (blue lines), RCP85 (red lines), errors in accuracy (green lines), and precision (purple lines). Accuracy was defined as the difference between multimodel average projections and actual data and precision as the standard deviation among the projections of all models. Comparison of these frequency distributions illustrates that errors in accuracy and precision are insufficient to offset projected changes in surface temperature, oxygen, and pH. Note that in those cases, accuracy (green lines) is centered to zero, meaning that for the great majority of cells the multimodel average prediction was identical to actual observations. Errors in precision were often larger, but they are added to both sides of the projections, meaning that they will broaden expected projections but will not reverse them. However, in the case of surface productivity and all parameters at the seafloor, errors in accuracy and precision were larger than the projected change, highlighting the need for caution in those cases. Further details are presented in Table S2; the performance of individual models is shown in Table S1.

We found that the average of all models was always closer to actual observations than any model was individually (Tables S1, S2). Thus, errors in precision were often larger than those in accuracy (Figure 1). That is, there were often large differences among the suite of model predictions, but their multimodel average was often closer to actual observations (Tables S1, S2, Figure 1). We also found that the accuracy of the multimodel average varied by parameter and ocean domain. Specifically, there was a stronger predictability of temperature, oxygen, and pH at the ocean surface and a lower predictability of phytoplankton carbon concentration and of all parameters at the seafloor (Figure 1; complete results and details of accuracy and precision plus Taylor diagrams are presented in Tables S1, S2). This low predictability may emerge from the limited availability of actual observations [this may be the case for “phytoplankton carbon concentration” and “particulate organic carbon flux,” which are modeled from other parameters, and there is often a significant disagreement among available products of such parameters (Page 5 in Table S2)] [11] and the inevitable complexity of deep-water processes, which may remain poorly modeled by Earth System Models. With these considerations in mind, we used results based on the upper layer of the ocean and the multimodel average, unless otherwise indicated.

It is worth noticing that discrepancies between Earth System Models outputs and present-day climate observation are partly due to the fact that these models simulate their own internal climate variability (i.e., complex, nonlinear interactions among different components such as atmosphere, ocean, ice, physics, biogeochemistry, etc.) rather than those observed in reality. Thus, a perfect match between any individual model output and observations is unlikely for all places and times. However, these offsetting errors between a given global model and current-day observations have been found to be ameliorated by averaging the output of multiple models (e.g., this study, [52]). This property of multimodel averaging is likely to be just as useful in future climate projections, which highlight the key reason for using the broad range of available models in future predictions of the climate, including those models with moderate capacity to predict current observations [50],[52].

### Future Projections in Ocean Biogeochemistry

In this study, we analyzed ocean biogeochemical projections under two alternative pathways in which CO**_2_** concentrations could increase to 550 and 900 ppm by 2100 (as reference, atmospheric CO_2_ concentrations are now at ∼400 ppm from 280 ppm in pre-industrial times; see Figure S1 [1],[48],[53]). These two scenarios are based on Representative Concentration Pathways 4.5 (RCP45) and 8.5 (RCP85) and represent alternative mitigation efforts between a concerted rapid CO_2_ mitigation and a “business-as-usual” scenario, respectively [48]; there is a more aggressive mitigation scenario called RCP26, which we do not use because it was not consistently used among models and some consider it realistically unattainable (see Figure S1).

**Figure 2:**
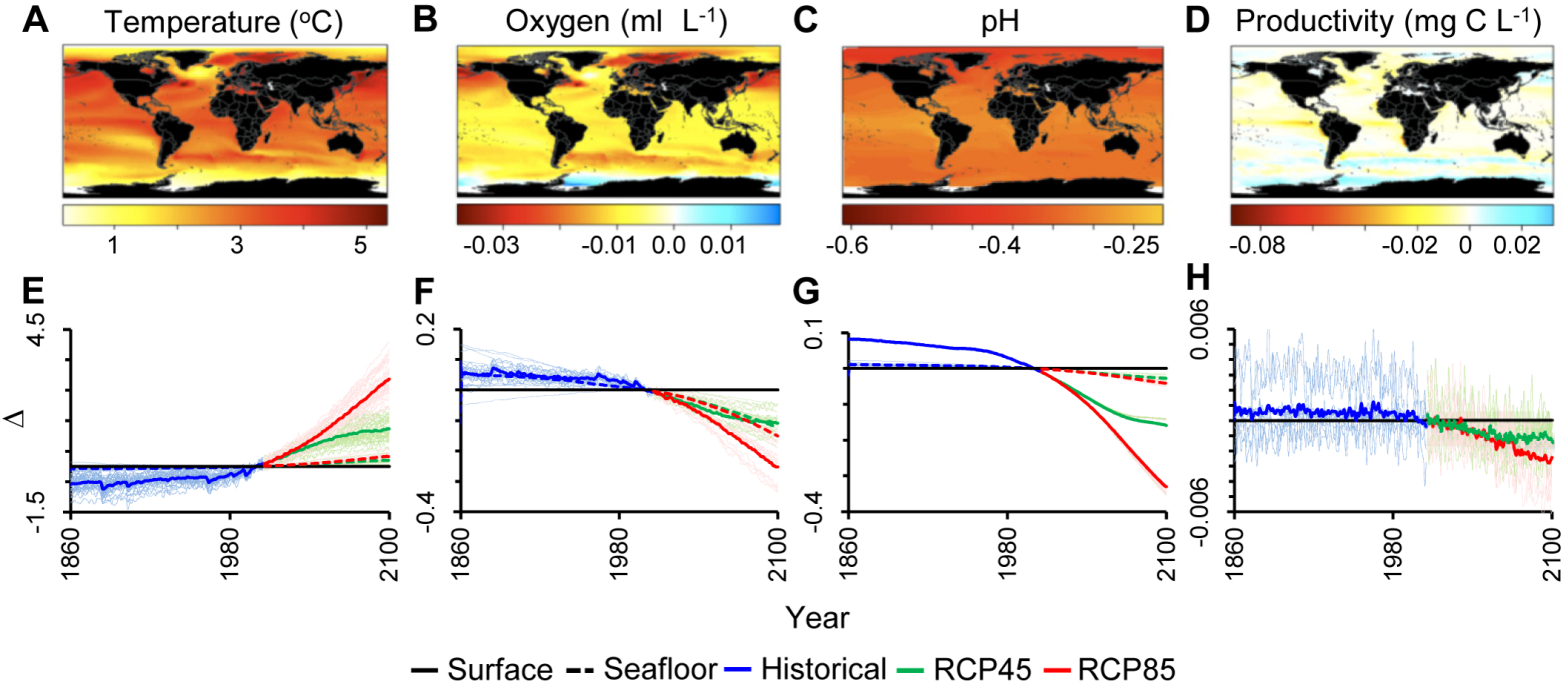
Future biogeochemistry change in the world's oceans. Plots A–D show the spatial difference between future (i.e., the average from 2091 to 2100) and contemporary (i.e., the average from years 1996 to 2005) values under the RCP85 scenario (decadal averages were chosen to minimize aliasing by interannual variability; beside each color scale we provide the absolute change, whereas the numbers on top indicate the rescaled values; complete results for the RCP85 and RCP45 for the ocean surface and floor are shown in Figure S2). Plots E–H show the global average change relative to contemporary values under the RCP45 and RCP85 at the ocean surface and seafloor; semitransparent lines are the projections for individual models.

Projections of biogeochemical parameters under RCP45 and RCP85 were variable in magnitude among analyzed Earth System Models (semitransparent lines in Figure 2E–H) but followed remarkably similar trends overall (solid lines in Figure 2E–H, Table S1). By 2100, global averages for the upper layer of the ocean could experience a temperature increase of 1.2 to 2.6°C (Figure 2E, Table S3), a dissolved oxygen concentration reduction of 0.11 to 0.24 ml l^−1^ (i.e., a ∼2% to 4% reduction of current values, Figure 2F, Table S3), a pH decline of 0.15 to 0.31 (Figure 2G, Table S3), and a diminished phytoplankton concentration of 0.001 to 0.003 mg C l^−1^ (i.e., a ∼4% to 10% reduction of current values, Figure 1H, Table S3) according to RCP45 and RCP85, respectively. In contrast, the world's seafloor was projected to experience smaller changes in temperature and pH (i.e., warming of 0.20 to 0.31°C and acidification of 0.03 to 0.04 pH units) but larger reductions in particulate carbon flux (i.e., food supply) reaching the seafloor (i.e., particulate carbon flux will decline 0.18 to 0.36 mg C m^−2^ y^−1^ or 6% to 13% reduction of current values, Table S3); reductions in dissolved oxygen will be similar to those observed at the sea surface (i.e., oxygen will decline by 0.11 to 0.14 ml l^−1^ compared to current values, Table S3); all values are according to RCP45 and RCP85, respectively.

**Figure 3:**
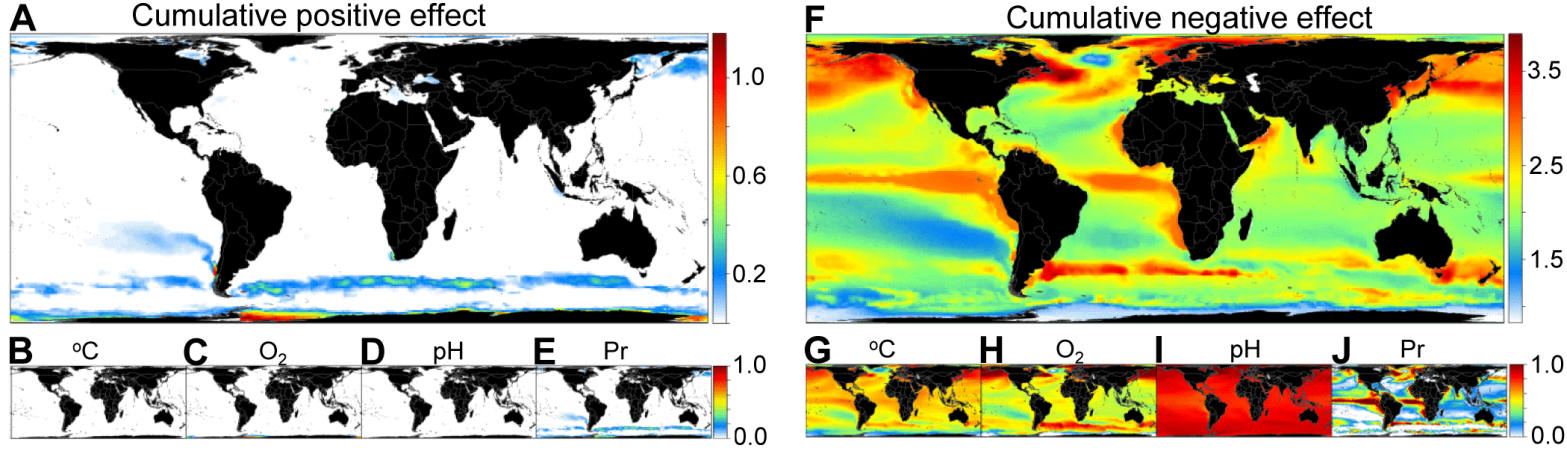
Co-occurring ocean biogeochemical changes to the year 2100 under the RCP85. For these plots, we separated absolute changes shown in Figure 2A–D between those that will be positive (i.e., cooling, basification, oxygenation, and productivity increase; Plots A–E) and negative (i.e., warming, acidification, oxygen depletion, and primary food reduction; Plots F–J). Resulting absolute changes were scaled between 0 and 1 (Plots B–E, G–J), 0 being zero absolute change and 1 being the extreme 97.5% observed value globally. The resulting scaled scores from each variable were added to provide a global composite map of co-occurring positive (Plot A) and negative (Plot F) changes in ocean biogeochemistry. These cumulative change maps ranged from 4 (i.e., the maximum predicted change in all four parameters occurred in that cell) to 0 (i.e., no negative or positive change in any of the four parameters occurred in that cell). The results for the RCP45 at the ocean surface and both RCPs for the seafloor are presented in the Supporting Information section.

**Figure 4:**
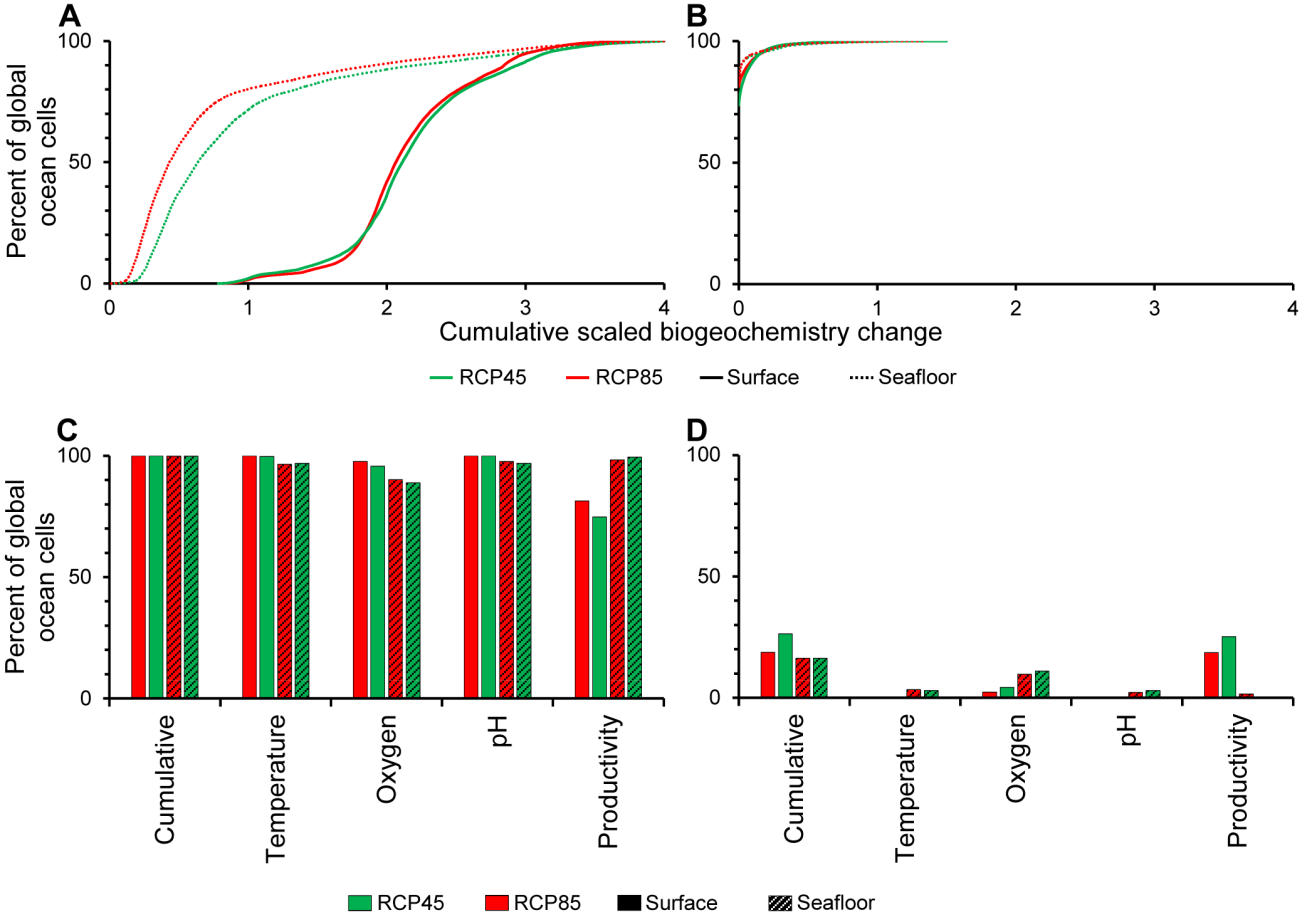
Exposure of the world's oceans to co-occurring changes in ocean biogeochemistry to the year 2100. (A–B) are the cumulative percentage of cells globally exposed to the composite score of co-occurring ocean biogeochemistry changes (see Figure 3 for details). (A) is for negative and (B) for the positive biogeochemistry changes. (C–D) is the discrimination of total ocean cells globally exposed to negative (C) and positive (D) changes in each variable and the composite score.

By 2100, projected changes in temperature, dissolved oxygen, pH, and primary food supply vary significantly among regions (Figure 2A–D). For the ocean surface, the smallest projected changes for pH are in the tropics, for temperature and productivity in temperate regions, and for oxygen in the Southern Ocean (Antarctica). At the seafloor, all variables analyzed experienced the largest changes along continental margins, with decreasing oxygen being common over larger areas of the world's seafloor, particularly at the poles (Figure S2). In general, however, with the exception of the Antarctic and small areas in the South Pacific and North Atlantic, most of the world's oceans will be simultaneously exposed to change in all parameters (Figures 3–4, Figure S2). With the exception of productivity and all parameters at the seafloor, current errors in accuracy and precision of the Earth System Models are of insufficient magnitude to offset projected changes; that is, projected changes in temperature, oxygen, and pH in the upper ocean layer were larger than their errors in accuracy and precision, meaning that trends in these three parameters are robust and are unlikely to be reversed by current sources of model errors (Figure 1, Table S2).

To identify patterns of co-occurrence in biogeochemical changes, we differentiate changes in biogeochemistry that are negative (i.e., warming, acidification, oxygen depletion, and primary food reduction) from those that are positive (i.e., cooling, basification, oxygenation, and productivity increase). Note that the terms “negative” and “positive” are used to indicate the direction of biogeochemical changes, not their potential effects upon biodiversity or social systems. The resulting values were then scaled from 0 to 1 (i.e., 0 meaning no change and 1 the upper 97.5% most extreme absolute change predicted in the world). The scaled-scores for each biogeochemical parameter were added to generate a composite global map of “negative” and “positive” changes in ocean biogeochemistry (Figure 3). The composite global scores were differentiated between “positive” and “negative” changes to avoid neutralization of biogeochemical changes (e.g., a cell with a warming score of −1 and a productivity increase score of 1 will yield a composite global score of 0, which would be confounded with no change). Additionally, separation of the global composite scores into positive and negative changes allows a better appreciation of the preponderance of the directions of biogeochemical change in the world's oceans.

The results of this analysis indicate that the entire ocean surface will be impacted by warming, acidification, or reductions in oxygen and productivity (Figure 4A,C)—over 99% by the largest negative change in at least one full parameter (Figure 4A). In contrast, only oxygen and productivity will experience positive changes at the surface over a small fraction (Figure 4D) of the polar regions (Figure 3C,E); almost no place in the world's ocean surface will face cooling or pH increase (Figure 3B,D). Co-occurring negative changes will also occur extensively over the world's ocean seafloor (Figure 4C,D), although the magnitude of such changes will be smaller: only about 20%–27% of the ocean's seafloor will be exposed to the largest negative change projected in more than one biogeochemical parameter (Figure 4A). Patterns of co-occurrence in biogeochemical parameters were very similar between the RCP45 and RCP85 (Figure 4).

### Biological Exposure to Ocean Biogeochemistry Change

**Figure 5:**
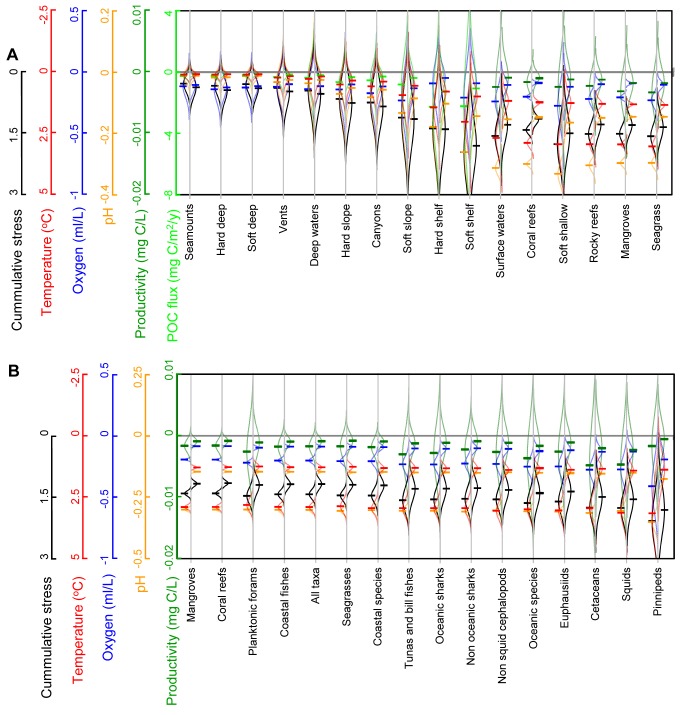
Future ocean biogeochemistry change on marine habitats and biodiversity hotspots. Here we show the mean (horizontal dashes) and standard deviation (curved lines) of the absolute change in each parameter projected to the year 2100 for each marine habitat (Plot A) and biodiversity hotspot for individual taxa (Plot B). A hotspot is defined as the top 10% most diverse (in number of species) areas on Earth where the given taxa are found [54]. In both plots, values for each parameter are color-coded according to the left-hand axes. Values to the left and right of each habitat or hotspot indicate the expected results according to RCP85 and RCP45, respectively. Data on marine habitats were obtained mainly from Halpern et al. [59]; additional sources are indicated in the Table S6; data on biodiversity hotspots were obtained from Tittensor et al. [54]. Complete results of the exposure of each habitat and hotspots to all parameters as well as the sources of error due to accuracy and precision are presented in Table S4. Particulate organic carbon flux (or simply carbon flux in the legend) applies only to seafloor habitats.

By overlaying the global distribution of marine habitats and hotspots of biodiversity for individual taxa with the projected changes in temperature, oxygen, pH, and primary food supply, we found that, to varying degrees, all projected biogeochemical changes will occur simultaneously within all habitats and biodiversity hotspots (Figure 5; Table S4 provides detailed statistics for the change in each parameter at each marine habitat and biodiversity hotspot and sources of error owing to accuracy and precision in the Earth System Models). Among marine habitats, the smallest absolute changes in biogeochemical parameters are expected to occur in deep-sea habitats (e.g., soft- and hard-bottom benthos, seamounts, and vents; Figure 5A, Table S4), whereas the largest changes will likely occur in shallow-water habitats like coral and rocky reefs, seagrass beds, and shallow soft-bottom benthos (Figure 5A, Table S4). Like the biota, biodiversity hotspots (i.e., areas with high numbers of species of a particular taxon [54]) will also be differentially stressed by ocean biogeochemistry change (Figure 5B). Among biodiversity hotspots analyzed in this study, the smallest cumulative exposure to future biogeochemical change is projected to occur in hotspots of mangrove and coral reef species, whereas the largest exposure will occur in hotspots of euphausiid (i.e., krill; a crucial component of food webs at mid and high latitudes), cetacean, squid, and pinniped species (Figure 5B, Table S4).

For the purpose of assessing the co-occurrence of biogeochemical change, we considered all absolute changes in an additive manner; however, this is not to say that biological responses will follow an additive or linear response to such changes. Realistically, empirical data are unavailable for a sufficient number of species to predict the biological responses of an entire ecosystem to the exposure of biogeochemical change in the ocean (i.e., given variations in physiological adaptations, tolerance thresholds, nonlinear responses, ecological interactions, and resulting cascade effects, etc.). Even broad generalizations could be prone to limitations. For instance, it is often argued that diverse ecosystems can be resilient to climate change as redundancy in species functions could allow the buffering of species lost by climate change. This, in itself, implies a change in community structure [55], although empirical evaluation of this idea has suggested that, perhaps due to strong niche specialization, diverse ecosystems may actually exhibit reduced functional redundancy and be particularly prone to disturbances [56].

Despite our inability to predict the type and magnitude of biological responses to ocean biogeochemistry change, existing knowledge suggests that ocean biogeochemical changes could exert a major selective pressure upon species and have the capability to reorganize patterns of body size, abundance, distribution, species richness, and ecosystem functioning (Table 1). Biological and ecological responses are likely to be magnified, especially if in interaction with other stressors [10],[28], as there will be a need for multiple physiological adaptations. The expected biological response is further highlighted by the biological changes already observed in certain monitored ecosystems in response to recent environmental change. Coral reefs, in which massive bleaching and growth reduction have been linked to relatively minor contemporary warming and acidification [3],[57], provide an excellent example of this. Even deep-sea ecosystems, for which the magnitude of biogeochemical shifts will be smaller (dotted lines in Figure 2E–G), may undergo substantial biological responses, mainly because the deep ocean is much more stable, and thus its faunas are likely adapted to narrower ranges of environmental variation than those in shallow marine habitats [14],[58]. We reemphasize that a standing challenge is to determine the preponderance of taxa from different marine habitats and ecosystems that will be sensitive to ocean biogeochemistry change.

### Vulnerability of Coastal People to Ocean Biogeochemistry Change

Here we quantified the relative vulnerability of coastal people to ocean biogeochemistry change in the traditional sense of exposure to environmental change, dependency of potentially impacted ocean goods and services, and social adaptability [34],[35],[44],[45].

We determined the level of exposure of each Exclusive Economic Zone in the world to the cumulative negative ocean biogeochemistry change, on a scale ranging between 0 (i.e., no ocean biogeochemistry change) and 4 (i.e., maximum observed biogeochemistry change in all four analyzed parameters) (data from Figure 3B; we analyzed only negative changes given their overwhelming coverage globally, and because those changes are likely to have the largest impacts on the supply of ocean goods and services). For the purpose of classification, cumulative negative biogeochemical changes were divided into three equal bins to classify countries with low, medium, and high exposure to ocean biogeochemistry change.

To quantify levels of dependency, we used three different metrics of peoples' dependence on the ocean: jobs, revenues, and food. Job dependency was measured as the fraction of the countries' work force employed by marine fishing, the marine tourism industry, mariculture, and marine mammal watching. Revenue dependency was measured as the fraction of a country's Gross Domestic Product (GDP) generated by revenues from marine tourism, fishing, mariculture, and marine mammal watching. Food dependency was the fraction of animal protein consumption supplied by seafood. All three dependencies were added and divided in three equal bins to indicate countries of low, medium, and high dependency.

Societal adaptability to environmental change was quantified as per capita GDP, assuming that richer countries will have more alternatives, higher capacity, and adaptability. For the purpose of classification, we defined low-, medium-, and high-income countries depending on whether annual per capita GDP was smaller than US$4,000, between US$4,000 and US$12,000 and larger than US$12,000, respectively (sources of data are presented in Table S6).

**Figure 6:**
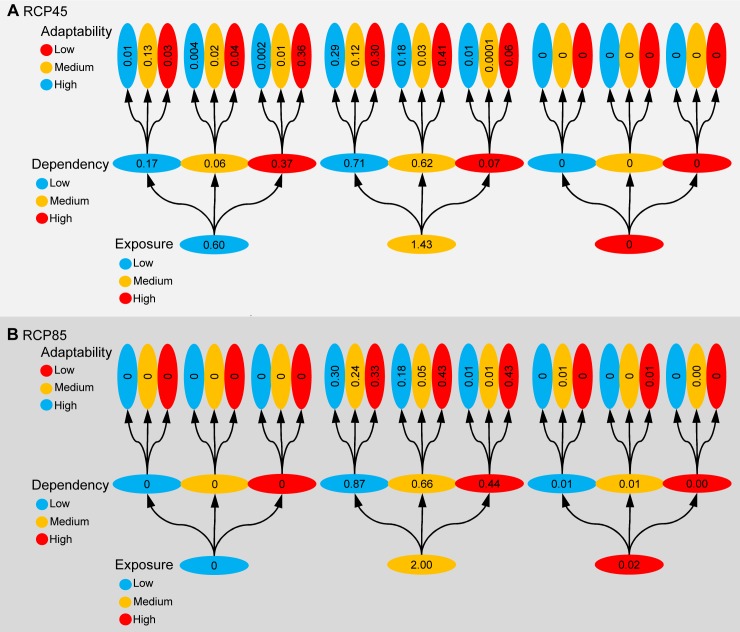
Vulnerability of humans to projected ocean biogeochemistry change. This plot illustrates the total number of people likely to be vulnerable through exposure to ocean biogeochemistry change according to RCP45 (Plot A) and RCP85 (Plot B). Numbers in the plot are in billions (summations may not be exact owing to rounding). Categorization of people according to their levels of exposure to biogeochemical changes, dependency on ocean goods and services, and social adaptability is described in the main text.

For each country, we estimated the number of coastal people (i.e., living within 50 km of the coast) within each category of exposure, dependency, and adaptability (we provide global summaries in the main text and detailed country results in the Supporting Information section). We found that approximately 1.4 billion people live in the coastal areas of countries whose Exclusive Economic Zones will experience medium to high ocean biogeochemistry change by 2100 under the RCP45. Of those, ∼690 million live in countries with a medium to high ocean dependence, and of these ∼470 million live in low-income countries (Figure 6A). The situation will be more dramatic under the RCP85, according to which 2.02 billion coastal people will live in countries with medium to high ocean biogeochemistry change; of those, 1.12 billion live in countries of medium to high ocean dependence; and of these, ∼870 million live in low-income countries (Figure 6B; detailed statistics of the change in each biochemical parameter at each Exclusive Economic Zone and sources of error owing to accuracy and precision in the Earth System Models are shown in Table S5). These results highlight the considerable challenges for human adaptability likely to emerge from ocean biogeochemistry change. Not only does a considerable fraction of the world's human population constantly use resources that will be impacted by ocean climate change, but such people are also located in developing countries with low capacity for adaptation to climate change. This limited socioeconomic capacity could also hamper the ability to benefit from “positive” ecosystem changes, if such new opportunities require costly adaptability [38].

### Concluding Remarks

Although a mechanistic model of how ocean biogeochemical changes alter biological and social systems will be difficult to develop, existing knowledge suggests that the responses to the exposure of expected ocean biogeochemical change could be considerable. First, the array of interrelated parameters affected by increasing CO_2_ emissions provide a much more worrisome picture than consideration of single stressors alone, as most of the world's oceans will be influenced by changes in multiple biogeochemical parameters, and thus adaptation will require multiple physiological adjustments from marine species. Additionally, there is the potential for synergistic responses to co-occurring stressors, and indirect ecological releases and trophic cascades. Secondly, human dependence on marine goods and services is also substantial in countries that will experience considerable ocean biogeochemistry change, particularly among low-income countries. This highlights the looming vulnerability to climate change in developing/low-income countries, and an unfortunate disparity between those who benefit economically from the processes creating climate change and those who will have to pay most of the environmental and social costs. The kind of biogeochemical stressors identified here will be further compounded by sea level rise, which has already been identified as a major potential socioeconomic consequence from climate change. These results provide a refined and synoptic numerical projection of change in key biogeochemical parameters upon marine biota and human societies, and indicate that if global CO_2_ emissions are not reduced, substantial degradation of marine ecosystems and associated human hardships are very likely to occur.

## Methods

Our analysis builds on recent ocean physical and biochemical projections developed as part of the Coupled Model Intercomparison Project Phase 5 to the Fifth Assessment Report of the Intergovernmental Panel on Climate Change [47]. As of July 2012, there were 31 Earth System Models from 18 centers in nine countries that modeled at least one of the ocean parameters analyzed here (Table S1). For analysis, all parameters were interpolated into a common 1° by 1° grid (assessment of multiple interpolation methods is provided in the Supplement S1). In total, over 27,000 years of data from the different models and variables were processed. Given the number and size of the files, we used several tools to optimize data processing, which are made available in Supplement S2. To quantify the robustness of Earth System Models, we compared projections among models (to measure model precision) and with actual data (to measure model accuracy) (data sources are indicated in Table S6). The multimodel average projections in the different biogeochemical parameters, in response to the analyzed CO_2_ scenarios, were overlapped with the distribution of different marine habitats and biodiversity hotspots to calculate how much individual and combined change will occur upon each habitat and hotspot (additional details are provide in Figure 5 and Table S4). Finally, for each Exclusive Economic Zone in the world, we calculated the projected cumulative change in all biogeochemical parameters analyzed here (Figure 3B), and quantified human vulnerability to this change by using country-level data on current social resilience (in terms of wealth and assuming that richer countries will have more alternatives, higher capacity, and adaptability) and human dependence for ocean goods and services arising from food, jobs, and revenue (Results for individual countries are shown in Table S5 and data sources in Table S6.)

## Supporting Information

Figure S1 **Representative concentration pathways.**
(DOCX)

Figure S2 **Absolute and scaled changes in temperature, oxygen, pH, and primary food availability to the year 2100 at the ocean surface and floor.**
(DOCX)

Table S1 **Models used and their individual results.**
(DOCX)

Table S2 **Accuracy and precision of multi-model predictions.**
(DOCX)

Table S3 **Ocean biogeochemistry change by available models.**
(DOCX)

Table S4 **Expected climate change on marine habitats and biodiversity hotspots.**
(DOCX)

Table S5 **Climate change by Exclusive Economic Zone and vulnerability of coastal people given their dependency of the ocean and wealth.**
(DOCX)

Table S6 **Source of data.**
(DOCX)

Supplement S1 **Standardization of grid resolutions among models.**
(DOCX)

Supplement S2 **R scripts for processing CMIP5 files.**
(DOCX)

## Abbreviations

CMIP5: the Coupled Model Intercomparison Project Phase 5
GDP: gross domestic product
POC flux: particulate organic carbon flux
RCP: representative concentration pathway
