# Supplementary material for: Biotic and Human Vulnerability to Projected Changes in Ocean Biogeochemistry over the 21st Century
Source: PLoS Biol. 2013 Oct 15;11(10):e1001682. doi: 10.1371/journal.pbio.1001682 (PMC3797030; doi:10.1371/journal.pbio.1001682)
Supplement: Figure S1 — Representative concentration pathways. (DOCX) [file pbio.1001682.s001.docx]

Fig. S1 Representative Concentration Pathways. This plot is intended to illustrate the alternative greenhouse gas concentrations for the Representative Concentration Pathways (RCPs) and their extensions to 2100. These projections include all major anthropogenic greenhouse gases. For details see Meinshausen et al. [[1](#_ENREF_1)]. Data from http://forge.ipsl.jussieu.fr/igcmg/wiki/ConcentrationsGHG


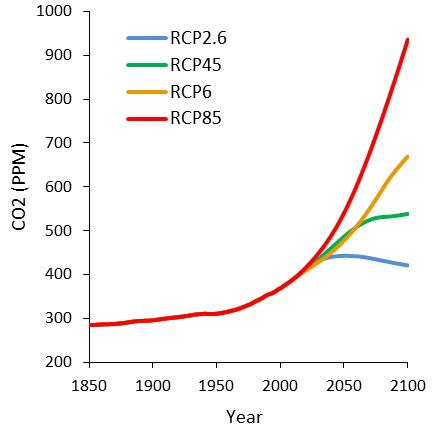


**References**

1. Meinshausen M, Smith SJ, Calvin K, Daniel JS, Kainuma MLT, et al. (2011) The RCP greenhouse gas concentrations and their extensions from 1765 to 2300. Climatic Change 109: 213-241.
