# Supplementary material for: Biotic and Human Vulnerability to Projected Changes in Ocean Biogeochemistry over the 21st Century
Source: PLoS Biol. 2013 Oct 15;11(10):e1001682. doi: 10.1371/journal.pbio.1001682 (PMC3797030; doi:10.1371/journal.pbio.1001682)

**Figure S2. Absolute and scaled changes in temperature, oxygen, pH, and primary food availability to the year 2100 at the ocean surface and floor.** This is the extended version of Fig. 1 in the main paper. Difference between projected (i.e. the average from 2091 to 2100) and contemporary (i.e. the average from years 1996 to 2005) values of temperature, oxygen, pH and primary food supply under the two RCP scenarios considered at the ocean surface and floor. The cumulative change is basically the addition of the scaled values of each parameter (a value of four indicates that the maximum predicted change in all four parameters occurred in that cell whereas a value of zero indicates that no negative change in any of the four parameters occurred in that cell (the values to the right of the scale are the absolute change whereas the numbers to the left are the scaled values.


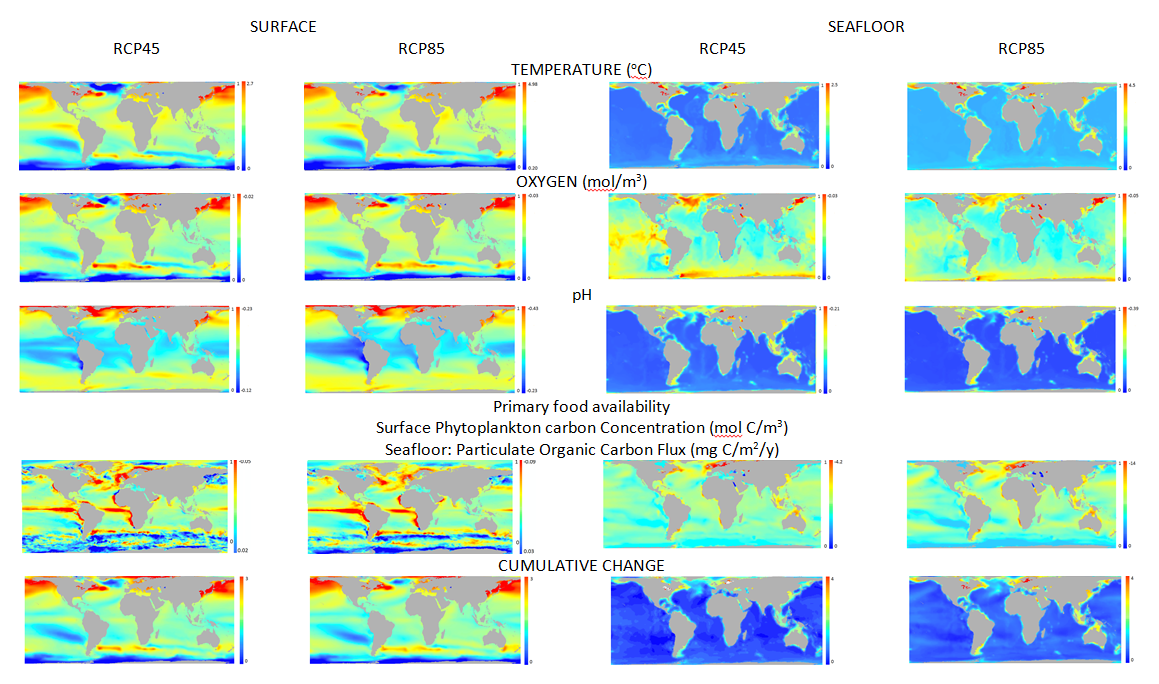

Supplement: Figure S2 — Absolute and scaled changes in temperature, oxygen, pH, and primary food availability to the year 2100 at the ocean surface and floor. (DOCX) [file pbio.1001682.s002.docx]
