# Supplementary material for: Biotic and Human Vulnerability to Projected Changes in Ocean Biogeochemistry over the 21st Century
Source: PLoS Biol. 2013 Oct 15;11(10):e1001682. doi: 10.1371/journal.pbio.1001682 (PMC3797030; doi:10.1371/journal.pbio.1001682)
Supplement: Table S1 — Models used and their individual results. (DOCX) [file pbio.1001682.s003.docx]

Table S1. Models used and their individual results. This table summarizes all models used in this study for each variable. Each tick mark contain a URL, where you will find detailed results for each model, including animations of the temporal change of each model, profiles of the projected change to the year 2100, and model skills in predicting the given parameter, summarized with Taylor diagrams.

| **Center** | **Country** | **Model** | **Contact** | **Sea Water Potential Temperature** | **Productivity/ Sinking Particulate Organic Carbon Flux***^5^* | **Dissolved Oxygen Concentration***^6^* | **pH** |
| --- | --- | --- | --- | --- | --- | --- | --- |
| Commonwealth Scientific and Industrial Research Organisation | Australia | ACCESS1-0 | Mark Collier | [√](http://www.soc.hawaii.edu/mora/CMIP5/thetao_ACCESS1-0.html) |  |  |  |
|  |  | ACCESS1-3 | *mark.collier@csiro.au* | [√](http://www.soc.hawaii.edu/mora/CMIP5/thetao_ACCESS1-3.html) |  |  |  |
| Bureau of Meteorology |  | CSIRO-Mk3-6-0 |  | [√](http://www.soc.hawaii.edu/mora/CMIP5/thetao_CSIRO-Mk3-6-0.html) |  |  |  |
| Beijing Climate Center | China | BCC-CSM1.1 | Tongwen Wu | [√](http://www.soc.hawaii.edu/mora/CMIP5/thetao_bcc-csm1-1.html) |  |  |  |
|  |  | BCC-CSM1.1.m | *twwu@cma.gov.cn* | [√](http://www.soc.hawaii.edu/mora/CMIP5/thetao_bcc-csm1-1-m.html) |  |  |  |
| Beijing Normal University | China | BNU-ESM |  | [√](http://www.soc.hawaii.edu/mora/CMIP5/thetao_BNU-ESM.html) |  |  |  |
| Centre National de Recherches Meteorologiques | France | CNRM-CM5 |  | [√](http://www.soc.hawaii.edu/mora/CMIP5/thetao_CNRM-CM5.html) |  |  |  |
| Canadian Centre for Climate Modelling and Analysis | Canada | CanESM2 | Jim Christian *Jim.Christian@ec.gc.ca* | [√](http://www.soc.hawaii.edu/mora/CMIP5/thetao_CanESM2.html) | [√](http://www.soc.hawaii.edu/mora/CMIP5/Productivity_CanESM2.html)^1^ |  | [√](http://www.soc.hawaii.edu/mora/CMIP5/ph_CanESM2.html) |
| Community Climate System Model | USA | CESM1-BGC |  | [√](http://www.soc.hawaii.edu/mora/CMIP5/thetao_CESM1-BGC.html) | [√](http://www.soc.hawaii.edu/mora/CMIP5/expc_CESM1-BGC.html) | [√](http://www.soc.hawaii.edu/mora/CMIP5/o2_CESM1-BGC.html) | [√](http://www.soc.hawaii.edu/mora/CMIP5/ph_CESM1-BGC.html) |
|  |  | CESM1-CAM5 | *cesm_data@ucar.edu* | [√](http://www.soc.hawaii.edu/mora/CMIP5/thetao_CESM1-CAM5.html) |  |  |  |
| National Center for Atmospheric Research | USA | CCSM4 |  | [√](http://www.soc.hawaii.edu/mora/CMIP5/thetao_CCSM4.html) |  |  |  |
| Chinese Academy of Sciences | China | FGOALS-s2 |  | [√](http://www.soc.hawaii.edu/mora/CMIP5/thetao_FGOALS-s2.html) |  |  |  |
|  |  | FGOALS-g2 |  | [√](http://www.soc.hawaii.edu/mora/CMIP5/thetao_FGOALS-g2.html) |  |  |  |
| First Institute of Oceanography | China | FIO-ESM |  | [√](http://www.soc.hawaii.edu/mora/CMIP5/thetao_FIO-ESM.html) |  |  |  |
| Geophysical Fluid Dynamics Laboratory | USA | GFDL-CM3 | John Dunne | [√](http://www.soc.hawaii.edu/mora/CMIP5/thetao_GFDL-CM3.html) |  |  |  |
|  |  | GFDL-ESM2G | *John.Dunne@noaa.gov* | [√](http://www.soc.hawaii.edu/mora/CMIP5/thetao_GFDL-ESM2G.html) | [√](http://www.soc.hawaii.edu/mora/CMIP5/Productivity_GFDL-ESM2G.html)^2^ | [√](http://www.soc.hawaii.edu/mora/CMIP5/o2_GFDL-ESM2G.html) | [√](http://www.soc.hawaii.edu/mora/CMIP5/ph_GFDL-ESM2G.html) |
|  |  | GFDL-ESM2M |  | [√](http://www.soc.hawaii.edu/mora/CMIP5/thetao_GFDL-ESM2M.html) | [√](http://www.soc.hawaii.edu/mora/CMIP5/Productivity_GFDL-ESM2M.html) | [√](http://www.soc.hawaii.edu/mora/CMIP5/o2_GFDL-ESM2M.html) | [√](http://www.soc.hawaii.edu/mora/CMIP5/ph_GFDL-ESM2M.html) |
| NASA Goddard Institute for Space Studies | USA | GISS-E2-R |  | [√](http://www.soc.hawaii.edu/mora/CMIP5/thetao_GISS-E2-R.html) |  |  |  |
| Met Office Hadley Centre | UK | HadGEM2-CC | Paul Halloran | [√](http://www.soc.hawaii.edu/mora/CMIP5/thetao_HadGEM2-CC.html) | [√](http://www.soc.hawaii.edu/mora/CMIP5/Productivity_HadGEM2-CC.html) | [√](http://www.soc.hawaii.edu/mora/CMIP5/o2_HadGEM2-CC.html) | [√](http://www.soc.hawaii.edu/mora/CMIP5/ph_HadGEM2-CC.html) |
|  |  | HadGEM2-ES | *paul.halloran@metoffice.gov.uk* | [√](http://www.soc.hawaii.edu/mora/CMIP5/thetao_HadGEM2-ES.html) | [√](http://www.soc.hawaii.edu/mora/CMIP5/Productivity_HadGEM2-ES.html) | [√](http://www.soc.hawaii.edu/mora/CMIP5/o2_HadGEM2-ES.html) | [√](http://www.soc.hawaii.edu/mora/CMIP5/ph_HadGEM2-ES.html) |
| Institut Pierre-Simon Laplace | France | IPSL-CM5A-LR | Laurent Bopp | [√](http://www.soc.hawaii.edu/mora/CMIP5/thetao_IPSL-CM5A-LR.html) | [√](http://www.soc.hawaii.edu/mora/CMIP5/Productivity_IPSL-CM5A-LR.html) | [√](http://www.soc.hawaii.edu/mora/CMIP5/o2_IPSL-CM5A-LR.html) | [√](http://www.soc.hawaii.edu/mora/CMIP5/ph_IPSL-CM5A-LR.html) |
|  |  | IPSL-CM5A-MR | *bopp@lsce.ipsl.fr* | [√](http://www.soc.hawaii.edu/mora/CMIP5/thetao_IPSL-CM5A-MR.html) | [√](http://www.soc.hawaii.edu/mora/CMIP5/Productivity_IPSL-CM5A-MR.html) | [√](http://www.soc.hawaii.edu/mora/CMIP5/o2_IPSL-CM5A-MR.html) | [√](http://www.soc.hawaii.edu/mora/CMIP5/ph_IPSL-CM5A-MR.html) |
|  |  | IPSL-CM5B-LR |  | [√](http://www.soc.hawaii.edu/mora/CMIP5/thetao_IPSL-CM5B-LR.html) | [√](http://www.soc.hawaii.edu/mora/CMIP5/Productivity_IPSL-CM5B-LR.html) | [√](http://www.soc.hawaii.edu/mora/CMIP5/o2_IPSL-CM5B-LR.html) | [√](http://www.soc.hawaii.edu/mora/CMIP5/ph_IPSL-CM5B-LR.html) |
| University of Tokyo | Japan | MIROC5 | Hideki Okajima | [√](http://www.soc.hawaii.edu/mora/CMIP5/thetao_MIROC5.html) |  |  |  |
|  |  | MIROC-ESM | *okajima@aori.u-tokyo.ac.jp* | [√](http://www.soc.hawaii.edu/mora/CMIP5/thetao_MIROC-ESM.html) |  |  |  |
|  |  | MIROC-ESM-CHEM |  | [√](http://www.soc.hawaii.edu/mora/CMIP5/thetao_MIROC-ESM-CHEM.html) |  |  |  |
| Max Planck Institute for Meteorology | Germany | MPI-ESM-LR | Tatiana Ilyina | [√](http://www.soc.hawaii.edu/mora/CMIP5/thetao_MPI-ESM-LR.html) | [√](http://www.soc.hawaii.edu/mora/CMIP5/Productivity_MPI-ESM-LR.html)^3^ | [√](http://www.soc.hawaii.edu/mora/CMIP5/o2_MPI-ESM-LR.html) | [√](http://www.soc.hawaii.edu/mora/CMIP5/ph_MPI-ESM-LR.html) |
|  |  | MPI-ESM-MR | *tatjana.ilyina@zmaw.de* | [√](http://www.soc.hawaii.edu/mora/CMIP5/thetao_MPI-ESM-MR.html) | [√](http://www.soc.hawaii.edu/mora/CMIP5/Productivity_MPI-ESM-MR.html)^3^ | [√](http://www.soc.hawaii.edu/mora/CMIP5/o2_MPI-ESM-MR.html) | [√](http://www.soc.hawaii.edu/mora/CMIP5/ph_MPI-ESM-MR.html) |
| Meteorological Research Institute | Japan | MRI-CGCM3 | NAKANO Hideyuki  *hnakano@mri-jma.go.jp* | [√](http://www.soc.hawaii.edu/mora/CMIP5/thetao_MRI-CGCM3.html) |  |  |  |
| Norwegian Climate Centre | Norway | NorESM1-M | Jerry Tjiputra | [√](http://www.soc.hawaii.edu/mora/CMIP5/thetao_NorESM1-M.html) |  |  |  |
|  |  | NorESM1-ME | *jtj061@bjerknes.uib.no* | [√](http://www.soc.hawaii.edu/mora/CMIP5/thetao_NorESM1-ME.html) | [√](http://www.soc.hawaii.edu/mora/CMIP5/Productivity_NorESM1-ME.html)^3^ | [√](http://www.soc.hawaii.edu/mora/CMIP5/o2_NorESM1-ME.html) | [√](http://www.soc.hawaii.edu/mora/CMIP5/ph_NorESM1-ME.html) |
| ***TOTAL NUMBER OF MODELS*** | | |  | 31 | 12 | 11 | 12 |

^1^ Sinking Particulate Organic Carbon Flux (i.e. CMIP5 variable name: expc) was not calculated directly by the model. However, Detrital Organic Carbon Concentration (i.e. CMIP5 variable name: detoc) was, which in turn, can be used to estimate expc by multiplying detoc by a flux speed of 0.000081 (m/s) or 7 (m/d). See below validation of the approach using models that calculated both detoc and Productivity.

^2^ Sinking Particulate Organic Carbon Flux (i.e. CMIP5 variable name: expc) was not specifically calculated by this model. However, the model calculated Detrital Nitrogen Concentrations (i.e. ndet_z in molN kg-1), which in turn can be converted to expc by multiply by the sinking velocity of 100 m/day, converting from mass to volume units (rho_0=1035 kg m-3), and from nitrogen to carbon (106/16). Specifically, expc=ndet_z*100/86400*1035*106/16.

^3^ The same as in ^1^ but these models used a flux speed of 0.000058 (m/s) or 5 (m/d).

^4^ As in ^2^, this model calculated sinking Nitrogen in mol N m-2 s-1. So we multiple the model’s output by a conversion factor of 7 to convert from Nitrogen to Carbon and by 12.0107 to convert from mol to grams of Carbon.

^5^ Sinking Particulate Organic Carbon Flux (i.e. CMIP5 variable name: expc) was originally measured in moles of Carbon per second but we converted to grams of Carbon per year by converting from moles to gram (*12.0107) and from seconds to year (*31536000). Phytoplankton Carbon Concentration was also converted from moles of Carbon to gram of Carbon by using the conversion factor of 12.0107.

^6^ Dissolved Oxygen Concentration was originally measured in moles but we converted to mL by using the conversion factor of moles to ml of oxygen of 22.391.

# Conversion from Detrital Organic Carbon Concentration (i.e. detoc; mol m-3) to Sinking Particulate Organic Carbon Flux (i.e. expc; mol m-2 s-1)

Data on the fluxes of different elements to the seafloor received variable attention among the different modeling groups of the CMIP5. As a result, of the eight research centers that modeled ocean biochemistry, five modeled Sinking Particulate Organic Carbon Flux (i.e. expc). The other three, however, did model Detrital Organic Carbon Concentration (i.e. detoc), which can be converted to expc by its multiplication with the sinking speed used in the model. To validate this conversion, we compared the data from detoc and expc from the models that calculated both (see plot below). This comparison reveals the difference between models that used variable sinking speeds (e.g. IPSL) and those that use a constant speed (e.g. GDFL-ESM2). Regardless of this difference, it is clear, however, the high correlation between detoc and expc and the use of the former to predict the later.

The three modeling groups that calculated detoc but not expc, assumed a constant sinking speed, which we used to calculate expc from detoc. The exact math used in those three cases was:

$$\boldsymbol{expc=detoc*a*b*c}$$

where ***a*** is the constant sinking speed used in the model [i.e. 0.000058 m/s (equivalent to 5 m/day) for the NorESM1-ME and MPI-ESM-LR models and 0.000081 (m/s) (equivalent to 7 (m/d) for the CanESM2 model]. ***b*** is the conversion factor from moles to grams of carbon (i.e. 12.0107) and ***c*** is the conversion factor from seconds to year (i.e. 31536000 sec in one year). The resulting value is ***expc*** in grams of Carbon per square meter per year.


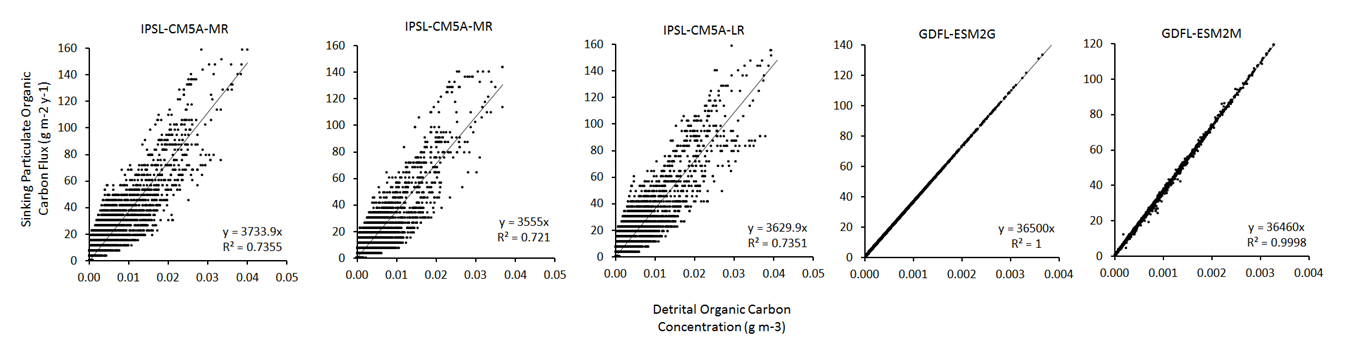
.
