# Supplementary material for: Biotic and Human Vulnerability to Projected Changes in Ocean Biogeochemistry over the 21st Century
Source: PLoS Biol. 2013 Oct 15;11(10):e1001682. doi: 10.1371/journal.pbio.1001682 (PMC3797030; doi:10.1371/journal.pbio.1001682)

**Table S2. Accuracy and precision of multi-model predictions.** Accuracy is defined as the proximity of the multimodel prediction to the actual value. To measure accuracy, we subtracted the multimodel prediction (average from 1996 to 2005) to actual data obtained from different sources (see Table S6 for data sources). Precision is defined as the standard deviation in the prediction of current conditions from all models. For the purpose of illustrating the effects of accuracy and precision on future multimodel predictions (assuming that those sources of error remain the same), we show the frequency distribution of projected changes to the year 2100 under the RCP45 and RCP85 as well as the frequency distribution of the errors due to accuracy and precision. Comparison of these frequency distributions illustrate that errors in accuracy and precision are insufficient to offset projected changes in surface temperature, oxygen and pH. However, in the case of surface primary food availability and all parameters at the seafloor, errors in accuracy and precision were larger than the projected change highlighting the need for caution in those cases. In the positive note, in all cases, models under-predicted current conditions in the seafloor, suggesting multimodels predictions are likely a conservative indication of the expected change at the seafloor. In the case of primary food availability at the surface, we also show how different products of actual data on primary productivity differ considerably among them and thus the poor model predictability of such a parameter should not be taken completely against model skills. Similar caution also needs to be considered for the case of carbon flux to the seafloor, for which actual data is based on models as well. In the case of temperature, oxygen and pH at the seafloor, poor model predictability seems to indicate some remaining limitations in the models at predicting complex three dimension processes of the oceans. To further illustrate model skills, we also provide Taylor diagrams, in which the red point indicates the multimodel average; blue point is the perfect match and black points are the predictions of all models. Given the overlap among black points we avoid naming individual models. For performance of individuals models please see Table S1.


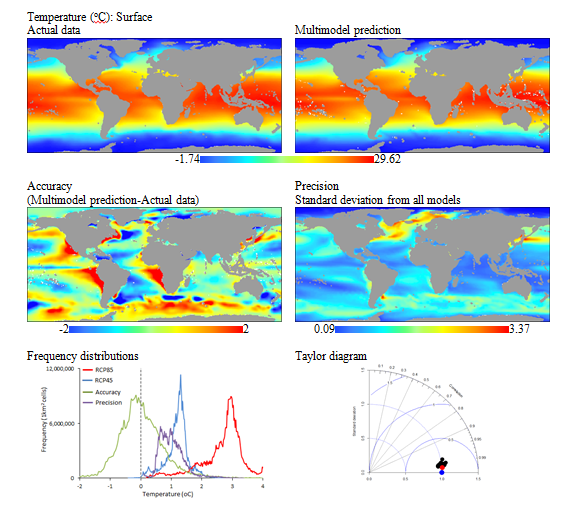


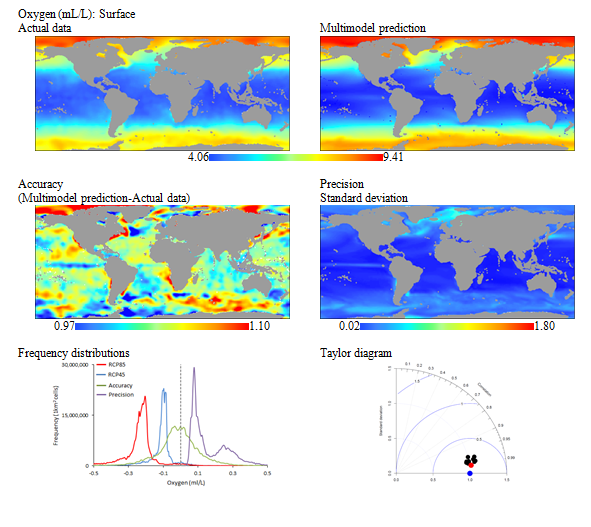


pH: Surface. Actual data on pH was estimated from indirect variables such as carbon, nutrients, temperature, salinity and pressure (see sources of data for details). Errors in each of such parameters are likely to propagate in the estimation of pH, and thus actual data on pH should be considered with caution. Remarkably and despite of this, errors in multimodel accuracy were very small (see frequency distributions plot: green line). There were, however, some places, mainly polar regions, where actual values of pH were higher than predicted by the multimodel. Excluding those places improved considerably the multimodel skills but also suggest that projected multimodel acidification in those regions is likely underestimated.


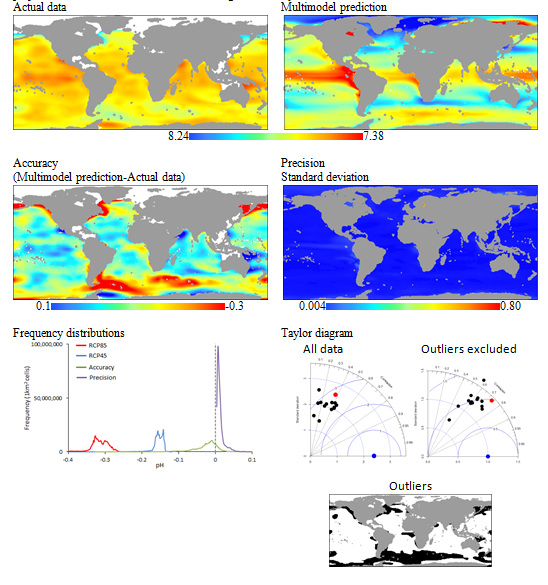


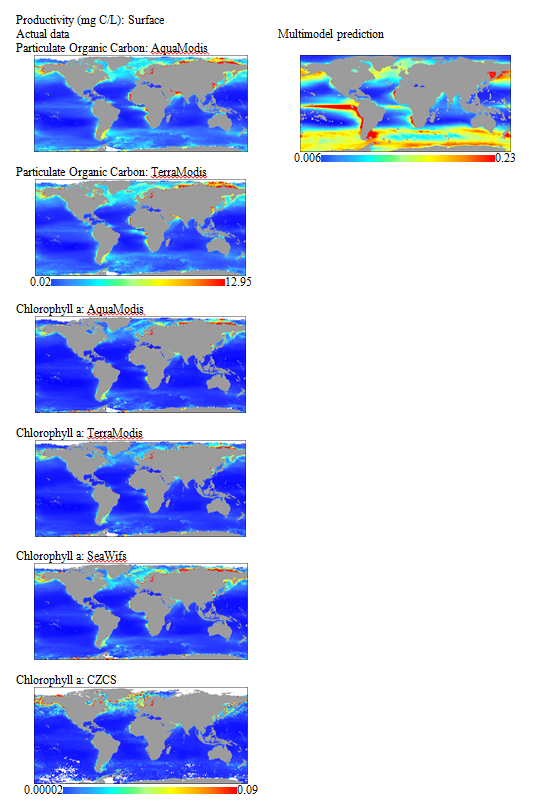


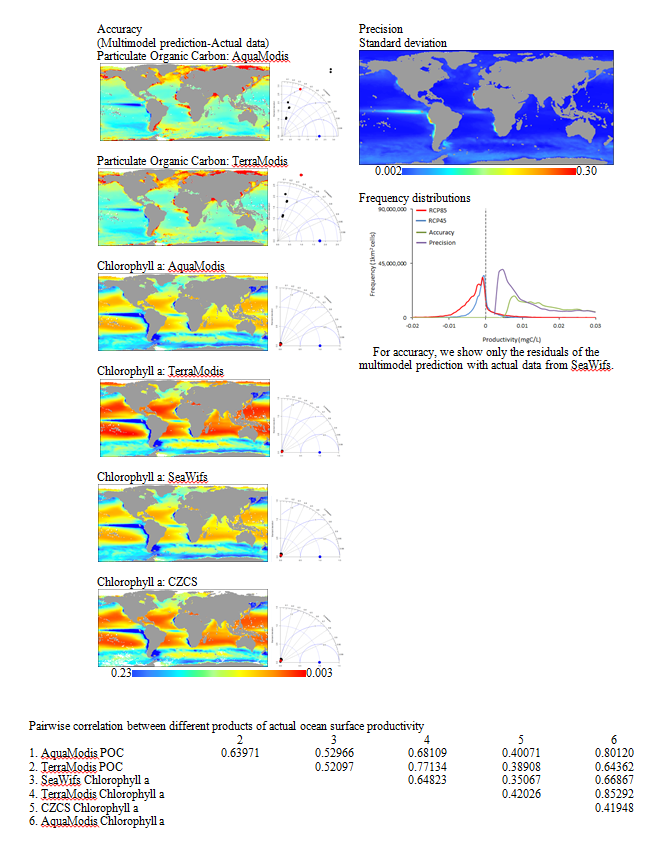


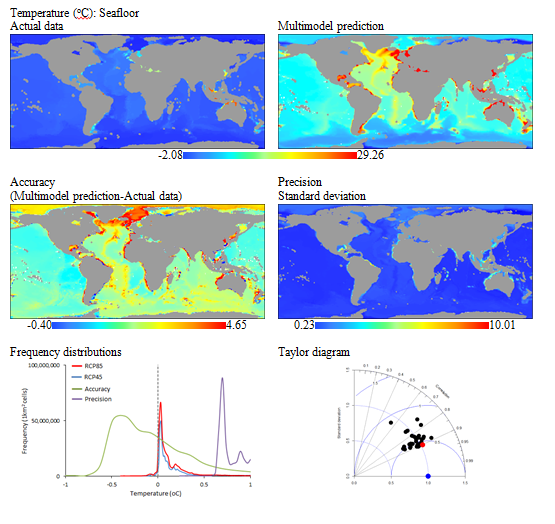


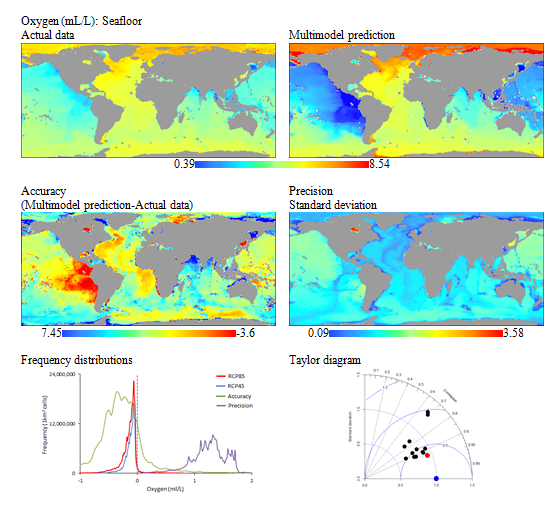


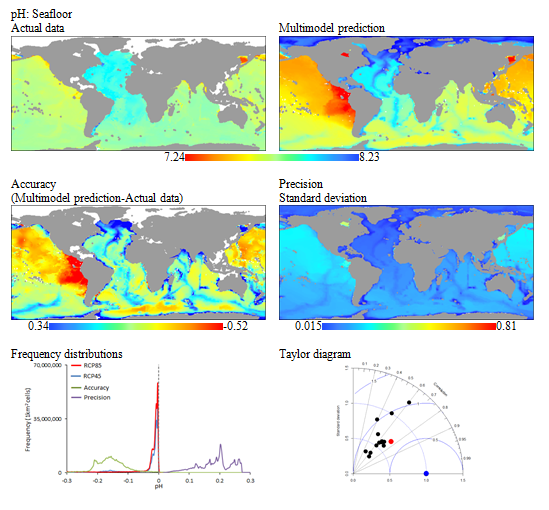


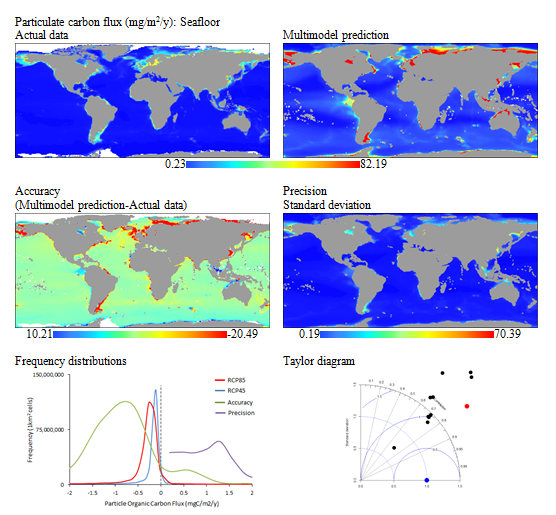

Supplement: Table S2 — Accuracy and precision of multi-model predictions. (DOCX) [file pbio.1001682.s004.docx]
