# Supplementary material for: Biotic and Human Vulnerability to Projected Changes in Ocean Biogeochemistry over the 21st Century
Source: PLoS Biol. 2013 Oct 15;11(10):e1001682. doi: 10.1371/journal.pbio.1001682 (PMC3797030; doi:10.1371/journal.pbio.1001682)
Supplement: Table S3 — Ocean biogeochemistry change by available models. (DOCX) [file pbio.1001682.s005.docx]

Table S3. Ocean biogeochemistry change by available models. This table shows the global absolute and relative change expected in the different parameters under the RCP45 an RCP85. Absolute change was calculated as the average (2091-2100) minus the average (1996-2005). Between brackets, we provide the relative change (as percentage), which is simply the absolute change divided by the average (1996-2005) times 100.

| **SURFACE** | | | | | | | | |
| --- | --- | --- | --- | --- | --- | --- | --- | --- |
|  | **RCP45** | | | | **RCP 85** | | | |
| **Model** | **Temperature**  **(^o^C)** | **Oxygen**  **(ml/L)** | **pH** | **Productivity**  **(mg C/L)** | **Temperature**  **(^o^C)** | **Oxygen**  **(ml/L)** | **pH** | **Productivity**  **(mg C/L)** |
| MultiModel | 1.181 (6.5) | -0.106 (-1.9) | -0.153 (-1.9) | -0.001 (-3.7) | 2.645 (14.5) | -0.236 (-4.3) | -0.307 (-3.9) | -0.003 (-9.9) |
| ACCESS1-0 | 1.411 (7.7) |  |  |  | 2.795 (15.2) |  |  |  |
| ACCESS1-3 | 1.422 (7.7) |  |  |  | 2.846 (15.5) |  |  |  |
| bcc-csm1-1 | 0.976 (5.4) |  |  |  | 2.396 (13.2) |  |  |  |
| bcc-csm1-1-m | 0.874 (4.7) |  |  |  | 2.294 (12.3) |  |  |  |
| BNU-ESM | 1.324 (7.1) |  |  |  | 2.979 (16.1) |  |  |  |
| CanESM2 | 1.402 (7.7) |  | -0.138 (-1.7) | 0 (1.5) | 3.054 (16.7) |  | -0.321 (-4) | 0 (0.5) |
| CCSM4 | 1.026 (5.5) |  |  |  | 2.47 (13.2) |  |  |  |
| CESM1-BGC | 0.953 (5.1) | -0.092 (-1.7) | -0.138 (-1.7) |  | 2.384 (12.7) | -0.223 (-4.1) | -0.323 (-4.1) |  |
| CESM1-CAM5 | 1.587 (8.7) |  |  |  | 2.853 (15.7) |  |  |  |
| CNRM-CM5 | 1.263 (6.9) |  |  |  | 2.511 (13.7) |  |  |  |
| CSIRO-Mk3-6-0 | 1.51 (8.8) |  |  |  | 2.863 (16.8) |  |  |  |
| FGOALS-g2 | 0.807 (4.5) |  |  |  | 2.044 (11.5) |  |  |  |
| FGOALS-s2 | 0.878 (4.7) |  |  |  | 3.318 (17.9) |  |  |  |
| FIO-ESM | 0.78 (4.3) |  |  |  | 2.515 (13.7) |  |  |  |
| GFDL-CM3 | 1.766 (9.7) |  |  |  | 3.31 (18.2) |  |  |  |
| GFDL-ESM2G | 0.48 (2.6) | -0.037 (-0.7) | -0.139 (-1.7) | 0 (-1.7) | 1.824 (10) | -0.157 (-2.9) | -0.318 (-4) | -0.001 (-6.6) |
| GFDL-ESM2M | 0.714 (3.8) | -0.06 (-1.1) | -0.139 (-1.7) | 0 (-2) | 1.833 (9.9) | -0.159 (-2.9) | -0.316 (-4) | -0.001 (-6) |
| GISS-E2-R | 0.78 (4.1) |  |  |  | 1.771 (9.4) |  |  |  |
| HadGEM2-CC | 1.433 (8) | -0.142 (-2.5) | -0.327 (-4) | -0.002 (-7.1) | 3.188 (17.8) | -0.308 (-5.6) | -0.141 (-1.8) | -0.005 (-14.8) |
| HadGEM2-ES | 1.626 (9) | -0.157 (-2.8) | -0.14 (-1.7) | -0.003 (-8.1) | 3.226 (17.8) | -0.311 (-5.7) | -0.326 (-4.1) | -0.005 (-15.1) |
| IPSL-CM5A-LR | 1.452 (8.3) | -0.134 (-2.3) | -0.139 (-1.7) | -0.001 (-6) | 3.309 (18.9) | -0.298 (-5.3) | -0.319 (-4) | -0.002 (-14.8) |
| IPSL-CM5A-MR | 1.58 (8.8) | -0.15 (-2.6) | -0.138 (-1.7) | -0.001 (-7.4) | 3.32 (18.5) | -0.289 (-5.2) | -0.318 (-4) | -0.002 (-15.7) |
| IPSL-CM5B-LR | 1.13 (6) | -0.105 (-1.9) | -0.138 (-1.7) | -0.001 (-5.7) | 2.364 (12.7) | -0.208 (-3.8) | -0.319 (-4) | -0.002 (-14.1) |
| MIROC5 | 1.064 (5.8) |  |  |  | 2.365 (12.8) |  |  |  |
| MIROC-ESM | 1.635 (9.1) |  |  |  | 3.407 (19) |  |  |  |
| MIROC-ESM-CHEM | 1.7 (9.5) |  |  |  | 3.692 (20.7) |  |  |  |
| MPI-ESM-LR | 1.012 (5.6) | -0.097 (-1.7) | -0.138 (-1.7) | -0.001 (-1.6) | 2.484 (13.7) | -0.231 (-4.2) | -0.322 (-4.1) | -0.005 (-10.5) |
| MPI-ESM-MR | 1.026 (5.6) | -0.098 (-1.8) | -0.138 (-1.7) | -0.001 (-2.7) | 2.399 (13.1) | -0.224 (-4.1) | -0.322 (-4.1) | -0.004 (-8.6) |
| MRI-CGCM3 | 1.126 (6.1) |  |  |  | 2.326 (12.7) |  |  |  |
| NorESM1-M | 0.994 (5.5) |  |  |  | 2.086 (11.5) |  |  |  |
| NorESM1-ME | 0.968 (5.4) | -0.096 (-1.7) | -0.139 (-1.7) | 0 (-0.6) | 2.157 (12.1) | -0.213 (-3.8) | -0.325 (-4.1) | -0.001 (-3.3) |
| UVic-ESCM-2-9-TOEP | 1.094 (6) | -0.103 (-1.9) | -0.14 (-1.7) |  | 2.27 (12.4) | -0.21 (-3.9) | -0.322 (-4.1) |  |

| **SEAFLOOR** | | | | | | | | |
| --- | --- | --- | --- | --- | --- | --- | --- | --- |
|  | **rcp45** | | | | **rcp85** | | | |
| **Model** | **Temperature**  **(^o^C)** | **Oxygen**  **(ml/L)** | **pH** | **Carbon Flux**  **(mg C/m2/y)** | **Temperature**  **(^o^C)** | **Oxygen**  **(ml/L)** | **pH** | **Carbon Flux**  **(mg C/m2/y)** |
| MultiModel | 0.197 (8.6) | -0.112 (-2.6) | -0.026 (-0.3) | -0.177 (-6.3) | 0.309 (13.4) | -0.136 (-3.2) | -0.038 (-0.5) | -0.362 (-12.9) |
| ACCESS1-0 | 0.203 (8.8) |  |  |  | 0.364 (15.7) |  |  |  |
| ACCESS1-3 | 0.186 (9.5) |  |  |  | 0.338 (17.2) |  |  |  |
| bcc-csm1-1 | 0.124 (4.2) |  |  |  | 0.241 (8.1) |  |  |  |
| bcc-csm1-1-m | 0.121 (4.1) |  |  |  | 0.234 (7.8) |  |  |  |
| BNU-ESM | 0.285 (7.6) |  |  |  | 0.374 (10) |  |  |  |
| CanESM2 | 0.137 (12.7) |  | -0.014 (-0.2) | -0.262 (-8.5) | 0.226 (20.9) |  | -0.025 (-0.3) | -0.613 (-19.7) |
| CCSM4 | 0.145 (8.2) |  |  |  | 0.243 (13.7) |  |  |  |
| CESM1-BGC | 0.152 (7.4) | -0.122 (-2.5) | -0.021 (-0.3) | -0.193 (-6.4) | 0.262 (12.8) | -0.144 (-3) | -0.035 (-0.4) | -0.367 (-12.2) |
| CESM1-CAM5 | 0.185 (9.6) |  |  |  | 0.269 (14) |  |  |  |
| CNRM-CM5 | 0.188 (5.9) |  |  |  | 0.295 (9.3) |  |  |  |
| CSIRO-Mk3-6-0 | 0.161 (9.3) |  |  |  | 0.254 (14.7) |  |  |  |
| FGOALS-g2 | 0.137 (6.8) |  |  |  | 0.252 (12.6) |  |  |  |
| FGOALS-s2 | 0.075 (4.4) |  |  |  | 0.232 (13.6) |  |  |  |
| FIO-ESM | 0.151 (10) |  |  |  | 0.27 (18) |  |  |  |
| GFDL-CM3 | 0.278 (9.5) |  |  |  | 0.424 (14.5) |  |  |  |
| GFDL-ESM2G | 0.228 (10.7) | -0.137 (-2.7) | -0.032 (-0.4) | -0.256 (-8.2) | 0.326 (15.3) | -0.156 (-3) | -0.047 (-0.6) | -0.49 (-15.7) |
| GFDL-ESM2M | 0.159 (5.1) | -0.124 (-2.8) | -0.029 (-0.4) | -0.252 (-7.7) | 0.255 (8.2) | -0.15 (-3.4) | -0.042 (-0.5) | -0.468 (-14.2) |
| GISS-E2-R | 0.181 (7.4) |  |  |  | 0.265 (10.8) |  |  |  |
| HadGEM2-CC | 0.237 (9.2) | -0.154 (-3.5) | -0.043 (-0.6) | -0.068 (-2.5) | 0.373 (14.6) | -0.174 (-4) | -0.029 (-0.4) | -0.148 (-5.4) |
| HadGEM2-ES | 0.271 (10.4) | -0.173 (-4) | -0.03 (-0.4) | -0.097 (-3.6) | 0.401 (15.4) | -0.2 (-4.6) | -0.044 (-0.6) | -0.161 (-5.9) |
| IPSL-CM5A-LR | 0.146 (9.4) | -0.109 (-3.5) | -0.033 (-0.4) | -0.39 (-7.3) | 0.247 (15.9) | -0.116 (-3.7) | -0.046 (-0.6) | -0.811 (-15.2) |
| IPSL-CM5A-MR | 0.142 (8) | -0.063 (-2.2) | -0.03 (-0.4) | -0.444 (-7.9) | 0.256 (14.4) | -0.11 (-3.8) | -0.045 (-0.6) | -0.866 (-15.4) |
| IPSL-CM5B-LR | 0.164 (7.9) | -0.109 (-5) | -0.021 (-0.3) | -0.241 (-5.9) | 0.244 (11.8) | -0.133 (-6.2) | -0.033 (-0.4) | -0.544 (-13.2) |
| MIROC5 | 0.215 (8.9) |  |  |  | 0.348 (14.5) |  |  |  |
| MIROC-ESM | 0.41 (18.7) |  |  |  | 0.546 (24.8) |  |  |  |
| MIROC-ESM-CHEM | 0.425 (20.5) |  |  |  | 0.57 (27.5) |  |  |  |
| MPI-ESM-LR | 0.197 (6.8) | -0.114 (-2.6) | -0.025 (-0.3) | -0.046 (-5.1) | 0.309 (10.7) | -0.141 (-3.2) | -0.039 (-0.5) | -0.112 (-12.4) |
| MPI-ESM-MR | 0.192 (6.4) | -0.101 (-2.3) | -0.025 (-0.3) | -0.043 (-4.3) | 0.316 (10.6) | -0.126 (-2.8) | -0.042 (-0.5) | -0.087 (-8.6) |
| MRI-CGCM3 | 0.14 (5.2) |  |  |  | 0.22 (8.1) |  |  |  |
| NorESM1-M | 0.278 (13) |  |  |  | 0.377 (17.6) |  |  |  |
| NorESM1-ME | 0.24 (10.1) | -0.044 (-0.7) | -0.024 (-0.3) | -0.005 (-0.4) | 0.339 (14.3) | -0.066 (-1) | -0.042 (-0.5) | -0.04 (-3.2) |
| UVic-ESCM-2-9-TOEP | 0.152 (8.6) | -0.096 (-2.2) | -0.017 (-0.2) | 0 (-0.2) | 0.222 (12.6) | -0.114 (-2.6) | -0.028 (-0.4) | 0.001 (0.3) |
