# Supplementary material for: Biotic and Human Vulnerability to Projected Changes in Ocean Biogeochemistry over the 21st Century
Source: PLoS Biol. 2013 Oct 15;11(10):e1001682. doi: 10.1371/journal.pbio.1001682 (PMC3797030; doi:10.1371/journal.pbio.1001682)
Supplement: Table S4 — Expected climate change on marine habitats and biodiversity hotspots. (DOCX) [file pbio.1001682.s006.docx]

| **HABITATS** | RCP45 | | RCP85 | | Accuracy | | Precision | |
| --- | --- | --- | --- | --- | --- | --- | --- | --- |
|  | Mean | SD | Mean | SD | Mean | SD | Mean | SD |
| **TEMPERATURE** (^o^C) | | | | | | | | |
| Canyons | 0.40 | 0.30 | 0.59 | 0.52 | 0.97 | 2.49 | 1.72 | 1.28 |
| Coral reefs | 1.26 | 0.11 | 2.90 | 0.21 | -0.32 | 0.45 | 0.68 | 0.25 |
| Deep water | 0.19 | 0.30 | 0.29 | 0.60 | 0.09 | 1.10 | 1.01 | 0.69 |
| Hard deep | 0.10 | 0.10 | 0.11 | 0.14 | 0.23 | 0.64 | 0.92 | 0.47 |
| Hard shelf | 0.81 | 0.42 | 1.45 | 0.84 | 0.46 | 4.54 | 3.10 | 1.97 |
| Hard slope | 0.35 | 0.34 | 0.51 | 0.58 | 0.99 | 2.30 | 1.83 | 1.45 |
| Mangroves | 1.32 | 0.08 | 2.96 | 0.13 | 0.25 | 0.00 | 0.77 | 0.19 |
| Rocky reefs | 1.31 | 0.18 | 2.96 | 0.31 | -0.19 | 0.61 | 0.76 | 0.27 |
| Seagrasses | 1.35 | 0.15 | 3.05 | 0.31 | -0.02 | 0.33 | 0.81 | 0.35 |
| Seamounts | 0.10 | 0.12 | 0.11 | 0.16 | 0.05 | 0.61 | 0.91 | 0.50 |
| Shoft shallow | 1.27 | 0.46 | 2.96 | 0.92 | -0.07 | 0.87 | 0.95 | 0.50 |
| Soft deep | 0.10 | 0.11 | 0.11 | 0.14 | 0.03 | 0.62 | 0.89 | 0.38 |
| Soft shelf | 1.00 | 0.46 | 2.04 | 0.96 | -0.30 | 3.66 | 2.16 | 1.43 |
| Soft slope | 0.57 | 0.44 | 0.95 | 0.86 | 0.92 | 2.60 | 1.74 | 1.21 |
| Surface water | 1.19 | 0.38 | 2.70 | 0.74 | 0.04 | 0.80 | 1.03 | 0.42 |
| Vents | 0.17 | 0.23 | 0.24 | 0.39 | 0.19 | 0.68 | 1.16 | 0.77 |
| **OXYGEN** (ml/L) | | | | | | | | |
| Canyons | -0.12 | 0.11 | -0.17 | 0.17 | -0.12 | 0.66 | 1.04 | 0.30 |
| Coral reefs | -0.09 | 0.01 | -0.20 | 0.02 | 0.07 | 0.07 | 0.09 | 0.03 |
| Deep water | -0.12 | 0.09 | -0.14 | 0.13 | -0.29 | 0.43 | 1.27 | 0.30 |
| Hard deep | -0.13 | 0.09 | -0.14 | 0.10 | -0.32 | 0.37 | 1.21 | 0.23 |
| Hard shelf | -0.05 | 0.10 | -0.09 | 0.18 | 0.12 | 0.67 | 0.91 | 0.35 |
| Hard slope | -0.10 | 0.10 | -0.14 | 0.16 | -0.24 | 0.70 | 1.01 | 0.31 |
| Mangroves | -0.09 | 0.01 | -0.21 | 0.02 | 0.12 | 0.00 | 0.11 | 0.03 |
| Rocky reefs | -0.10 | 0.03 | -0.22 | 0.06 | 0.09 | 0.14 | 0.11 | 0.05 |
| Seagrasses | -0.10 | 0.03 | -0.23 | 0.06 | -0.01 | 0.04 | 0.11 | 0.07 |
| Seamounts | -0.11 | 0.07 | -0.12 | 0.09 | -0.34 | 0.34 | 1.30 | 0.26 |
| Shoft shallow | -0.12 | 0.09 | -0.28 | 0.17 | -0.01 | 0.24 | 0.23 | 0.13 |
| Soft deep | -0.12 | 0.08 | -0.13 | 0.10 | -0.33 | 0.35 | 1.32 | 0.23 |
| Soft shelf | -0.09 | 0.15 | -0.21 | 0.25 | -0.07 | 1.04 | 0.96 | 0.64 |
| Soft slope | -0.13 | 0.13 | -0.23 | 0.22 | -0.01 | 0.75 | 0.97 | 0.35 |
| Surface water | -0.11 | 0.06 | -0.24 | 0.11 | -0.01 | 0.14 | 0.17 | 0.11 |
| Vents | -0.10 | 0.08 | -0.12 | 0.11 | -0.33 | 0.51 | 1.06 | 0.22 |
| **pH** | | | | | | | | |
| Canyons | -0.06 | 0.05 | -0.08 | 0.08 | -0.07 | 0.08 | 0.13 | 0.06 |
| Coral reefs | -0.15 | 0.01 | -0.30 | 0.01 | 0.03 | 0.04 | 0.01 | 0.01 |
| Deep water | -0.03 | 0.04 | -0.04 | 0.08 | -0.15 | 0.06 | 0.19 | 0.06 |
| Hard deep | -0.02 | 0.02 | -0.02 | 0.02 | -0.14 | 0.05 | 0.18 | 0.05 |
| Hard shelf | -0.10 | 0.06 | -0.18 | 0.11 | -0.07 | 0.08 | 0.11 | 0.06 |
| Hard slope | -0.05 | 0.05 | -0.07 | 0.08 | -0.08 | 0.09 | 0.14 | 0.06 |
| Mangroves | -0.15 | 0.01 | -0.30 | 0.01 | 0.02 | 0.00 | 0.02 | 0.01 |
| Rocky reefs | -0.15 | 0.01 | -0.31 | 0.02 | 0.02 | 0.05 | 0.02 | 0.01 |
| Seagrasses | -0.15 | 0.01 | -0.30 | 0.02 | 0.00 | 0.01 | 0.02 | 0.03 |
| Seamounts | -0.01 | 0.02 | -0.01 | 0.02 | -0.15 | 0.05 | 0.20 | 0.05 |
| Shoft shallow | -0.17 | 0.02 | -0.33 | 0.04 | -0.09 | 0.10 | 0.05 | 0.10 |
| Soft deep | -0.01 | 0.02 | -0.01 | 0.02 | -0.16 | 0.05 | 0.20 | 0.05 |
| Soft shelf | -0.14 | 0.05 | -0.26 | 0.09 | -0.09 | 0.10 | 0.13 | 0.11 |
| Soft slope | -0.09 | 0.06 | -0.13 | 0.11 | -0.06 | 0.08 | 0.12 | 0.07 |
| Surface water | -0.15 | 0.01 | -0.31 | 0.03 | -0.03 | 0.04 | 0.01 | 0.02 |
| Vents | -0.03 | 0.03 | -0.03 | 0.05 | -0.13 | 0.07 | 0.17 | 0.05 |
| **CARBON FLUX** (mg C/m^2^/y) | | | | | | | | |
| Canyons | -0.26 | 0.53 | -0.50 | 1.01 | -5.23 | 11.16 | 4.01 | 4.12 |
| Deep water | -0.17 | 0.64 | -0.35 | 1.09 | -1.41 | 5.66 | 2.25 | 4.41 |
| Hard deep | -0.10 | 0.11 | -0.21 | 0.22 | -0.81 | 1.61 | 1.30 | 1.26 |
| Hard shelf | -1.22 | 1.50 | -2.24 | 2.44 | -5.66 | 20.30 | 9.76 | 7.48 |
| Hard slope | -0.30 | 0.43 | -0.63 | 0.80 | -4.56 | 7.82 | 2.93 | 2.93 |
| Seamounts | -0.10 | 0.11 | -0.20 | 0.21 | -0.79 | 1.45 | 1.18 | 1.37 |
| Soft deep | -0.10 | 0.12 | -0.19 | 0.22 | -0.65 | 1.51 | 1.31 | 1.28 |
| Soft shelf | -1.03 | 2.17 | -2.20 | 3.55 | -15.49 | 22.19 | 15.94 | 10.70 |
| Soft slope | -0.35 | 0.98 | -0.78 | 1.77 | -6.48 | 11.73 | 5.65 | 6.38 |
| Vents | -0.13 | 0.19 | -0.29 | 0.41 | -1.70 | 4.02 | 1.64 | 2.13 |
| **PHYTOPLANKTON CONCENTRATION** (mg C/L) | | | | | | | | |
| Coral reefs | -0.0009 | 0.0009 | -0.0017 | 0.0018 | 0.0113 | 0.0025 | 0.0068 | 0.0052 |
| Mangroves | -0.0018 | 0.0021 | -0.0032 | 0.0036 | 0.0165 | 0.0000 | 0.0088 | 0.0076 |
| Rocky reefs | -0.0013 | 0.0017 | -0.0023 | 0.0027 | 0.0137 | 0.0052 | 0.0079 | 0.0055 |
| Seagrasses | -0.0019 | 0.0025 | -0.0034 | 0.0039 | 0.0147 | 0.0059 | 0.0103 | 0.0095 |
| Shoft shallow | -0.0013 | 0.0029 | -0.0025 | 0.0053 | 0.0262 | 0.0152 | 0.0149 | 0.0148 |
| Surface water | -0.0010 | 0.0028 | -0.0025 | 0.0052 | 0.0265 | 0.0186 | 0.0174 | 0.0182 |

Table S4. Expected climate change on marine habitats and biodiversity hotspots. Here we present the absolute change in the different parameters according to the RCP45 and RCP85 between current (i.e. average 1996-2005) and future (i.e. average 2091-2100) conditions. As potential sources of error, we provide the measures of accuracy and precision. Accuracy is the residual between the multi-model output for current conditions and actual data. Precision is the standard deviation among models in predicting current conditions.

**BIODIVERSITY HOTSPOTS**

| **TEMPERATURE** (^o^C) | | | | | | | | |
| --- | --- | --- | --- | --- | --- | --- | --- | --- |
| All coastal species | 1.31 | 0.14 | 2.95 | 0.24 | 0.01 | 0.45 | 0.70 | 0.30 |
| All taxa | 1.28 | 0.10 | 2.91 | 0.16 | -0.02 | 0.41 | 0.67 | 0.21 |
| Cetaceans | 1.34 | 0.26 | 2.96 | 0.50 | 0.08 | 1.24 | 1.20 | 0.52 |
| Coastal fishes | 1.28 | 0.10 | 2.90 | 0.14 | -0.09 | 0.46 | 0.68 | 0.20 |
| Coral reefs | 1.27 | 0.06 | 2.91 | 0.09 | 0.12 | 0.26 | 0.62 | 0.09 |
| Euphausiids | 1.38 | 0.22 | 3.05 | 0.42 | 0.10 | 0.95 | 1.08 | 0.51 |
| Forams | 1.25 | 0.13 | 2.82 | 0.26 | -0.21 | 0.57 | 0.86 | 0.22 |
| Mangroves | 1.28 | 0.06 | 2.91 | 0.08 | 0.02 | 0.31 | 0.61 | 0.09 |
| Non-oceanic sharks | 1.32 | 0.19 | 2.95 | 0.36 | 0.03 | 0.59 | 0.89 | 0.40 |
| Non-squid cephalopod | 1.39 | 0.19 | 3.06 | 0.35 | 0.00 | 0.56 | 0.82 | 0.39 |
| Oceanic sharks | 1.32 | 0.17 | 2.95 | 0.31 | 0.02 | 0.96 | 0.95 | 0.38 |
| Oceanic species | 1.36 | 0.20 | 3.00 | 0.38 | 0.16 | 1.12 | 1.07 | 0.46 |
| Pinnipeds | 1.38 | 0.71 | 3.17 | 1.32 | 0.18 | 0.85 | 1.49 | 0.66 |
| Seagrasses | 1.26 | 0.14 | 2.88 | 0.25 | 0.02 | 0.41 | 0.75 | 0.29 |
| Squids | 1.42 | 0.31 | 3.14 | 0.57 | 0.39 | 1.20 | 1.19 | 0.49 |
| Tunas and Bill fishes | 1.30 | 0.17 | 2.95 | 0.34 | -0.05 | 0.76 | 0.91 | 0.39 |
| **OXYGEN** (ml/L) | | | | | | | | |
| All coastal species | -0.09 | 0.02 | -0.21 | 0.04 | 0.02 | 0.09 | 0.09 | 0.04 |
| All taxa | -0.09 | 0.01 | -0.20 | 0.02 | 0.02 | 0.09 | 0.09 | 0.03 |
| Cetaceans | -0.13 | 0.05 | -0.28 | 0.08 | -0.03 | 0.18 | 0.18 | 0.10 |
| Coastal fishes | -0.09 | 0.01 | -0.20 | 0.02 | 0.01 | 0.09 | 0.09 | 0.03 |
| Coral reefs | -0.08 | 0.00 | -0.19 | 0.01 | 0.06 | 0.08 | 0.10 | 0.02 |
| Euphausiids | -0.12 | 0.05 | -0.25 | 0.08 | -0.05 | 0.14 | 0.14 | 0.11 |
| Forams | -0.10 | 0.01 | -0.22 | 0.02 | 0.01 | 0.06 | 0.10 | 0.04 |
| Mangroves | -0.08 | 0.00 | -0.19 | 0.01 | 0.06 | 0.08 | 0.10 | 0.03 |
| Non-oceanic sharks | -0.10 | 0.03 | -0.23 | 0.06 | 0.01 | 0.11 | 0.12 | 0.07 |
| Non-squid cephalopod | -0.11 | 0.04 | -0.23 | 0.07 | 0.02 | 0.10 | 0.11 | 0.07 |
| Oceanic sharks | -0.11 | 0.03 | -0.23 | 0.05 | -0.02 | 0.15 | 0.12 | 0.10 |
| Oceanic species | -0.12 | 0.04 | -0.25 | 0.07 | -0.04 | 0.15 | 0.14 | 0.08 |
| Pinnipeds | -0.19 | 0.11 | -0.41 | 0.21 | -0.08 | 0.20 | 0.33 | 0.12 |
| Seagrasses | -0.09 | 0.02 | -0.21 | 0.03 | 0.06 | 0.09 | 0.12 | 0.05 |
| Squids | -0.13 | 0.06 | -0.28 | 0.11 | -0.06 | 0.16 | 0.16 | 0.09 |
| Tunas and Bill fishes | -0.10 | 0.03 | -0.23 | 0.06 | 0.03 | 0.12 | 0.12 | 0.08 |
| **pH** | | | | | | | | |
| All coastal species | -0.15 | 0.01 | -0.31 | 0.01 | 0.00 | 0.04 | 0.01 | 0.01 |
| All taxa | -0.15 | 0.01 | -0.30 | 0.01 | 0.01 | 0.04 | 0.01 | 0.01 |
| Cetaceans | -0.16 | 0.01 | -0.32 | 0.02 | -0.03 | 0.04 | 0.01 | 0.01 |
| Coastal fishes | -0.15 | 0.01 | -0.30 | 0.01 | 0.02 | 0.04 | 0.01 | 0.01 |
| Coral reefs | -0.15 | 0.00 | -0.30 | 0.01 | 0.01 | 0.04 | 0.01 | 0.01 |
| Euphausiids | -0.15 | 0.01 | -0.31 | 0.01 | -0.02 | 0.03 | 0.01 | 0.00 |
| Forams | -0.15 | 0.01 | -0.30 | 0.01 | -0.01 | 0.02 | 0.01 | 0.01 |
| Mangroves | -0.15 | 0.00 | -0.30 | 0.01 | 0.00 | 0.05 | 0.02 | 0.01 |
| Non-oceanic sharks | -0.15 | 0.01 | -0.31 | 0.01 | 0.00 | 0.03 | 0.01 | 0.01 |
| Non-squid cephalopod | -0.15 | 0.01 | -0.31 | 0.02 | 0.00 | 0.04 | 0.02 | 0.04 |
| Oceanic sharks | -0.15 | 0.01 | -0.31 | 0.01 | -0.01 | 0.04 | 0.01 | 0.01 |
| Oceanic species | -0.15 | 0.01 | -0.31 | 0.02 | -0.02 | 0.04 | 0.01 | 0.01 |
| Pinnipeds | -0.18 | 0.02 | -0.35 | 0.03 | -0.10 | 0.08 | 0.02 | 0.04 |
| Seagrasses | -0.15 | 0.01 | -0.31 | 0.01 | 0.01 | 0.04 | 0.01 | 0.01 |
| Squids | -0.15 | 0.01 | -0.31 | 0.02 | -0.04 | 0.05 | 0.02 | 0.01 |
| Tunas and Bill fishes | -0.15 | 0.01 | -0.31 | 0.02 | 0.00 | 0.03 | 0.01 | 0.01 |
| **PHYTOPLANKTON CONCENTRATION** (mg C/L) | | | | | | | | |
| All coastal species | -0.0010 | 0.0008 | -0.0019 | 0.0014 | 0.0137 | 0.0045 | 0.0069 | 0.0035 |
| All taxa | -0.0009 | 0.0008 | -0.0017 | 0.0013 | 0.0132 | 0.0050 | 0.0067 | 0.0038 |
| Cetaceans | -0.0021 | 0.0038 | -0.0049 | 0.0075 | 0.0302 | 0.0264 | 0.0190 | 0.0315 |
| Coastal fishes | -0.0010 | 0.0009 | -0.0018 | 0.0015 | 0.0129 | 0.0057 | 0.0067 | 0.0040 |
| Coral reefs | -0.0009 | 0.0006 | -0.0016 | 0.0010 | 0.0141 | 0.0041 | 0.0067 | 0.0029 |
| Euphausiids | -0.0011 | 0.0024 | -0.0026 | 0.0045 | 0.0174 | 0.0140 | 0.0091 | 0.0145 |
| Forams | -0.0011 | 0.0024 | -0.0026 | 0.0046 | 0.0173 | 0.0177 | 0.0114 | 0.0199 |
| Mangroves | -0.0009 | 0.0006 | -0.0017 | 0.0010 | 0.0153 | 0.0041 | 0.0074 | 0.0033 |
| Non-oceanic sharks | -0.0009 | 0.0020 | -0.0023 | 0.0029 | 0.0167 | 0.0123 | 0.0091 | 0.0117 |
| Non-squid cephalopod | -0.0012 | 0.0027 | -0.0027 | 0.0047 | 0.0179 | 0.0173 | 0.0113 | 0.0214 |
| Oceanic sharks | -0.0012 | 0.0025 | -0.0029 | 0.0047 | 0.0173 | 0.0160 | 0.0104 | 0.0193 |
| Oceanic species | -0.0017 | 0.0031 | -0.0037 | 0.0059 | 0.0205 | 0.0186 | 0.0126 | 0.0219 |
| Pinnipeds | -0.0006 | 0.0030 | -0.0017 | 0.0049 | 0.0410 | 0.0138 | 0.0230 | 0.0110 |
| Seagrasses | -0.0008 | 0.0012 | -0.0017 | 0.0020 | 0.0196 | 0.0125 | 0.0097 | 0.0078 |
| Squids | -0.0024 | 0.0037 | -0.0048 | 0.0059 | 0.0249 | 0.0180 | 0.0164 | 0.0203 |
| Tunas and Bill fishes | -0.0013 | 0.0018 | -0.0031 | 0.0037 | 0.0221 | 0.0192 | 0.0123 | 0.0173 |
