# Supplementary material for: Biotic and Human Vulnerability to Projected Changes in Ocean Biogeochemistry over the 21st Century
Source: PLoS Biol. 2013 Oct 15;11(10):e1001682. doi: 10.1371/journal.pbio.1001682 (PMC3797030; doi:10.1371/journal.pbio.1001682)
Supplement: Table S5 — Climate change by Exclusive Economic Zone and vulnerability of coastal people given their dependency of the ocean and wealth. (DOCX) [file pbio.1001682.s007.docx]

Table S5. Climate change by Exclusive Economic Zone and vulnerability of coastal people given their dependency of the ocean and wealth. For each Exclusive Economic Zone, we calculate the cumulative and individual stress of all four parameters analyzed here (we focused only on parameters at the ocean surface). The number of coastal people was calculated as the number of people within 50km from the coast of each country; data on population was obtained from the Gridded human population database (<http://sedac.ciesin.columbia.edu/data/set/gpw-v3-population-count/data-download>). We quantify three different metric of peoples’ dependency on the ocean: jobs, revenues and food. Job dependency was measured as the fraction of the national work force employed by marine fishing, the marine tourism industry, mariculture and marine mammal watching; revenue dependency as the fraction of national Gross Domestic Product generated by revenues from marine tourism, fishing, mariculture and marine mammal watching; and, food dependency as the fraction of animal protein consumption supplied by seafood; all three dependencies were added and divided in three equal bins to indicate countries of Low (L), Medium (M) and High (H) dependency. Data on dependency were obtained from Halpern [[1](#_ENREF_1)] and complemented with data from World Bank <http://faostat3.fao.org/home/index.html#DOWNLOAD> and FAO <http://faostat3.fao.org/home/index.html#DOWNLOAD>. Wealth or people’s capacity to cope with the impact of environmental change was quantified as per capita GDP. For purpose of classification, we divided Low (L), Medium (M) and High (H) income countries depending on whether annual per capita GDP was smaller than US$4,000, between US$4,000 and US$12,000 and larger than US$12,000, respectively. Data on per capita GDP was obtained from the World Bank < <http://databank.worldbank.org/databank/download/WDIandGDF_excel.zip>>. Some countries may be missing from this table given the lack of complete social-economic data in the available source [[1](#_ENREF_1)].

|  | *Cumulative stress score* | | | |  |  | | | |
| --- | --- | --- | --- | --- | --- | --- | --- | --- | --- |
|  | *RCP85* | | *RCP45* | |  | *Categorized* | | | |
| *Country* | *Mean* | *SD* | *Mean* | *SD* | *Number of Coastal People* | *RCP85* | *RCP45* | *Dependency* | *Wealth* |
| Albania | 1.77 | 0.04 | 1.61 | 0.04 | 2,319,473 | M | M | L | L |
| Algeria | 1.65 | 0.04 | 1.44 | 0.07 | 16,673,221 | M | M | L | L |
| American Samoa | 1.48 | 0.22 | 1.22 | 0.22 | 518,784 | M | L | H | M |
| Amsterdam Island and Saint Paul Island | 1.56 | 0.08 | 1.26 | 0.12 | 0 | M | L |  |  |
| Angola | 2.07 | 0.24 | 1.95 | 0.24 | 5,059,029 | M | M | M | L |
| Anguilla | 1.41 | 0.10 | 1.19 | 0.13 | 216,002 | M | L | H | H |
| Antarctica | 0.71 | 0.23 | 0.51 | 0.18 | 0 | L | L |  |  |
| Antigua and Barbuda | 1.28 | 0.01 | 1.01 | 0.01 | 85,857 | L | L | H | H |
| Argentina | 1.90 | 0.50 | 1.52 | 0.44 | 17,657,580 | M | M | L | M |
| Ascension | 1.45 | 0.29 | 1.04 | 0.36 | 9,587 | M | L |  |  |
| Australia | 1.63 | 0.30 | 1.28 | 0.21 | 18,864,931 | M | L | L | H |
| Azores | 1.77 | 0.22 | 1.53 | 0.20 | 8,291,718 | M | M | M | H |
| Bahamas | 1.34 | 0.04 | 1.11 | 0.05 | 490,850 | M | L | H | H |
| Bahrain | 1.55 | 0.01 | 1.25 | 0.01 | 950,212 | M | L | H | H |
| Bangladesh | 1.50 | 0.05 | 1.35 | 0.06 | 41,843,795 | M | M | H | L |
| Barbados | 1.34 | 0.05 | 1.09 | 0.05 | 354,452 | M | L | H | M |
| Belgium | 2.35 | 0.02 | 2.26 | 0.02 | 2,146,247 | M | M | L | H |
| Belize | 1.42 | 0.02 | 1.22 | 0.02 | 287,434 | M | L | H | L |
| Benin | 1.79 | 0.09 | 1.65 | 0.13 | 3,720,956 | M | M | L | L |
| Bosnia and Herzegovina | 1.90 | 0.00 | 1.71 | 0.00 | 643,874 | M | M | L | L |
| Bouvet Island | 2.34 | 0.57 | 2.21 | 0.72 | 2,126,297 | M | M | M | H |
| Brazil | 1.53 | 0.28 | 1.28 | 0.32 | 79,397,939 | M | L | L | M |
| British Indian Ocean Territory | 1.47 | 0.01 | 1.17 | 0.03 | 0 | M | L |  |  |
| Cambodia | 1.39 | 0.02 | 1.14 | 0.02 | 1,819,182 | M | L | M | L |
| Cameroon | 1.59 | 0.02 | 1.37 | 0.02 | 2,297,252 | M | M | M | L |
| Canada | 2.10 | 0.46 | 1.78 | 0.51 | 14,910,276 | M | M | L | H |
| Canary Islands | 1.57 | 0.21 | 1.31 | 0.27 | 23,090,998 | M | M | L | H |
| Cape Verde | 1.67 | 0.13 | 1.44 | 0.15 | 714,489 | M | M | H | L |
| Chile | 1.27 | 0.24 | 1.08 | 0.27 | 5,899,596 | L | L | M | M |
| China | 1.94 | 0.48 | 1.76 | 0.50 | 219,540,736 | M | M | L | L |
| Christmas Island | 1.49 | 0.09 | 1.16 | 0.09 | 4,136 | M | L |  |  |
| Clipperton Island | 1.51 | 0.03 | 1.25 | 0.04 | 0 | M | L |  |  |
| Colombia | 1.62 | 0.14 | 1.42 | 0.21 | 9,356,811 | M | M | L | M |
| Comoros | 1.42 | 0.01 | 1.15 | 0.02 | 1,260,064 | M | L | M | L |
| Cook Islands | 1.49 | 0.33 | 1.18 | 0.31 | 4,047,923 | M | L | M | H |
| Costa Rica | 1.58 | 0.09 | 1.38 | 0.10 | 4,970,508 | M | M | L | M |
| Croatia | 1.95 | 0.10 | 1.75 | 0.05 | 1,556,494 | M | M | L | H |
| Cuba | 1.38 | 0.02 | 1.16 | 0.02 | 11,913,188 | M | L | L | M |
| Cyprus | 1.64 | 0.02 | 1.41 | 0.03 | 854,756 | M | M | M | H |
| Democratic Republic of the Congo | 1.94 | 0.11 | 1.78 | 0.14 | 340,744 | M | M | M | L |
| Denmark | 1.93 | 0.32 | 1.65 | 0.48 | 22,576,499 | M | M | L | H |
| Djibouti | 1.52 | 0.01 | 1.17 | 0.01 | 869,141 | M | L | L | L |
| Dominica | 1.32 | 0.03 | 1.07 | 0.04 | 95,281 | M | L | H | M |
| Dominican Republic | 1.35 | 0.04 | 1.11 | 0.05 | 9,959,779 | M | L | M | M |
| East Timor | 1.49 | 0.05 | 1.22 | 0.05 | 894,425 | M | L | L | L |
| Ecuador | 2.09 | 0.27 | 1.79 | 0.32 | 7,419,768 | M | M | L | L |
| Egypt | 1.51 | 0.07 | 1.25 | 0.08 | 24,044,054 | M | L | M | L |
| El Salvador | 1.66 | 0.10 | 1.50 | 0.06 | 6,324,486 | M | M | L | L |
| Equatorial Guinea | 1.83 | 0.13 | 1.69 | 0.18 | 441,875 | M | M | H | H |
| Eritrea | 1.46 | 0.03 | 1.15 | 0.02 | 1,255,268 | M | L | L | L |
| Estonia | 2.82 | 0.05 | 2.54 | 0.06 | 572,695 | H | M | L | H |
| Fiji | 1.34 | 0.05 | 1.05 | 0.04 | 1,129,555 | M | L | H | L |
| Finland | 2.84 | 0.07 | 2.54 | 0.07 | 1,957,730 | H | M | L | H |
| France | 1.76 | 0.12 | 1.59 | 0.18 | 15,271,951 | M | M | L | H |
| French Guiana | 1.57 | 0.06 | 1.30 | 0.07 | 186,611 | M | M | H | H |
| French Polynesia | 1.20 | 0.10 | 0.92 | 0.11 | 364,090 | L | L | L | H |
| Gabon | 1.92 | 0.11 | 1.79 | 0.14 | 1,012,110 | M | M | L | M |
| Gambia | 2.07 | 0.24 | 1.94 | 0.24 | 1,303,423 | M | M | H | L |
| Germany | 2.52 | 0.15 | 2.42 | 0.08 | 4,360,372 | M | M | L | H |
| Ghana | 1.80 | 0.16 | 1.60 | 0.19 | 10,050,707 | M | M | H | L |
| Gibraltar | 1.63 | 0.02 | 1.43 | 0.01 | 33,484 | M | M |  | H |
| Greece | 1.68 | 0.10 | 1.43 | 0.10 | 9,917,740 | M | M | M | H |
| Grenada | 1.63 | 0.11 | 1.45 | 0.17 | 134,285 | M | M | H | M |
| Guadeloupe | 1.30 | 0.03 | 1.04 | 0.04 | 1,081,208 | L | L | H | H |
| Guatemala | 1.62 | 0.04 | 1.42 | 0.05 | 2,483,037 | M | M | L | L |
| Guernsey | 2.08 | 0.27 | 2.00 | 0.31 | 111,946 | M | M | L | H |
| Guinea | 1.71 | 0.10 | 1.65 | 0.18 | 2,238,899 | M | M | M | L |
| Guinea-Bissau | 1.83 | 0.14 | 1.74 | 0.24 | 918,602 | M | M | L | L |
| Guyana | 1.66 | 0.11 | 1.45 | 0.10 | 625,614 | M | M | M | L |
| Haiti | 1.37 | 0.04 | 1.13 | 0.06 | 11,441,962 | M | L | L | L |
| Heard Island and McDonald Islands | 1.45 | 0.17 | 1.16 | 0.14 | 0 | M | L |  |  |
| Honduras | 1.45 | 0.03 | 1.24 | 0.04 | 3,763,203 | M | L | L | L |
| Iceland | 1.85 | 0.41 | 1.51 | 0.55 | 177,661 | M | M | H | H |
| India | 1.48 | 0.12 | 1.26 | 0.10 | 198,133,393 | M | L | M | L |
| Indonesia | 1.39 | 0.07 | 1.15 | 0.07 | 224,472,420 | M | L | H | L |
| Iran | 1.57 | 0.05 | 1.25 | 0.04 | 9,313,223 | M | L | L | L |
| Iraq | 1.48 | 0.00 | 1.19 | 0.00 | 446,899 | M | L | L | L |
| Ireland | 1.89 | 0.12 | 1.87 | 0.18 | 2,360,409 | M | M | L | H |
| Israel | 1.59 | 0.02 | 1.34 | 0.03 | 8,394,444 | M | M | L | H |
| Italy | 1.69 | 0.09 | 1.50 | 0.11 | 34,643,019 | M | M | L | H |
| Ivory Coast | 1.83 | 0.14 | 1.63 | 0.16 | 7,560,627 | M | M | M | L |
| Jamaica | 1.43 | 0.07 | 1.21 | 0.10 | 3,613,994 | M | L | H | M |
| Japan | 1.68 | 0.35 | 1.47 | 0.34 | 116,991,112 | M | M | M | H |
| Jarvis Island | 1.86 | 0.20 | 1.57 | 0.18 | 0 | M | M |  |  |
| Johnston Atoll | 1.49 | 0.09 | 1.21 | 0.08 | 0 | M | L |  |  |
| Jordan | 1.51 | 0.00 | 1.25 | 0.00 | 67,363 | M | L | L | L |
| Kenya | 1.54 | 0.03 | 1.30 | 0.05 | 3,480,576 | M | L | L | L |
| Kiribati | 1.52 | 0.28 | 1.27 | 0.28 | 124,368 | M | L | H | L |
| Kuwait | 1.49 | 0.01 | 1.19 | 0.01 | 2,439,086 | M | L | L | H |
| Latvia | 2.83 | 0.06 | 2.60 | 0.10 | 1,015,651 | H | M | L | M |
| Lebanon | 1.63 | 0.01 | 1.39 | 0.02 | 4,365,760 | M | M | M | M |
| Liberia | 1.72 | 0.09 | 1.52 | 0.10 | 2,776,090 | M | M | M | L |
| Libya | 1.55 | 0.03 | 1.29 | 0.03 | 4,589,621 | M | L | L | M |
| Lithuania | 2.83 | 0.03 | 2.64 | 0.03 | 268,618 | H | H | L | M |
| Madagascar | 1.42 | 0.04 | 1.18 | 0.07 | 8,317,494 | M | L | M | L |
| Malaysia | 1.41 | 0.05 | 1.21 | 0.05 | 28,078,948 | M | L | M | M |
| Maldives | 1.46 | 0.06 | 1.19 | 0.05 | 554,614 | M | L | H | M |
| Malta | 1.60 | 0.02 | 1.34 | 0.01 | 604,331 | M | M | H | H |
| Marshall Islands | 1.43 | 0.06 | 1.15 | 0.08 | 77,607 | M | L |  | L |
| Mauritania | 2.35 | 0.10 | 2.18 | 0.08 | 1,322,669 | M | M | H | L |
| Mauritius | 1.39 | 0.03 | 1.08 | 0.03 | 1,953,866 | M | L | H | M |
| Mexico | 1.55 | 0.14 | 1.36 | 0.16 | 27,747,544 | M | M | L | M |
| Micronesia | 1.40 | 0.06 | 1.15 | 0.09 | 217,472 | M | L | H | L |
| Monaco | 1.75 | 0.00 | 1.67 | 0.00 | 45,663 | M | M |  | H |
| Morocco | 1.48 | 0.15 | 1.27 | 0.15 | 19,567,189 | M | L | M | L |
| Mozambique | 1.42 | 0.03 | 1.16 | 0.04 | 11,424,908 | M | L | M | L |
| Myanmar | 1.34 | 0.05 | 1.17 | 0.06 | 17,921,319 | M | L | M | L |
| Namibia | 2.30 | 0.23 | 2.08 | 0.25 | 238,249 | M | M | M | L |
| Nauru | 1.65 | 0.14 | 1.44 | 0.12 | 17,958 | M | M | H | M |
| Netherlands | 2.37 | 0.03 | 2.32 | 0.05 | 7,095,690 | M | M | L | H |
| Netherlands Antilles | 1.71 | 0.27 | 1.59 | 0.36 | 458,053 | M | M | H | H |
| New Caledonia | 1.44 | 0.04 | 1.12 | 0.03 | 335,332 | M | L | L | M |
| Nicaragua | 1.55 | 0.11 | 1.39 | 0.12 | 4,863,367 | M | M | L | L |
| Nigeria | 1.68 | 0.06 | 1.49 | 0.08 | 25,400,720 | M | M | M | L |
| North Korea | 2.52 | 0.08 | 2.38 | 0.08 | 14,972,005 | M | M | M | L |
| Oman | 1.96 | 0.25 | 1.60 | 0.21 | 3,145,910 | M | M | H | H |
| Pakistan | 1.59 | 0.06 | 1.27 | 0.07 | 17,532,936 | M | L | L | L |
| Palau | 1.42 | 0.01 | 1.16 | 0.02 | 41,411 | M | L | H | M |
| Panama | 1.47 | 0.08 | 1.27 | 0.10 | 4,485,223 | M | L | M | M |
| Papua New Guinea | 1.41 | 0.05 | 1.16 | 0.09 | 3,533,912 | M | L | H | L |
| Peru | 2.21 | 0.12 | 1.92 | 0.28 | 18,221,101 | M | M | H | L |
| Philippines | 1.41 | 0.03 | 1.16 | 0.03 | 91,478,135 | M | L | H | L |
| Pitcairn | 1.13 | 0.08 | 0.89 | 0.07 | 68 | L | L |  |  |
| Poland | 2.81 | 0.04 | 2.59 | 0.05 | 2,619,724 | H | M | L | M |
| Prince Edward Islands | 1.50 | 0.16 | 1.29 | 0.13 | 14,192,484 | M | L | L | M |
| Puerto Rico | 1.33 | 0.03 | 1.07 | 0.03 | 5,889,217 | M | L | L | H |
| Qatar | 1.57 | 0.04 | 1.27 | 0.03 | 741,937 | M | L | H | H |
| Russia | 2.27 | 0.43 | 2.00 | 0.53 | 8,330,324 | M | M | L | M |
| Saint Kitts and Nevis | 1.30 | 0.01 | 1.03 | 0.01 | 46,340 | L | L | H | H |
| Saint Lucia | 1.39 | 0.03 | 1.14 | 0.04 | 220,462 | M | L | H | M |
| Saint Pierre and Miquelon | 2.70 | 0.21 | 2.64 | 0.14 | 7,340 | H | H | H | M |
| Saint Vincent and the Grenadines | 1.46 | 0.05 | 1.23 | 0.06 | 138,837 | M | L | H | M |
| Samoa | 1.26 | 0.01 | 1.00 | 0.01 | 211,723 | L | L | M | L |
| Sao Tome and Principe | 1.66 | 0.05 | 1.43 | 0.07 | 216,780 | M | M | H | L |
| Saudi Arabia | 1.45 | 0.04 | 1.16 | 0.05 | 4,602,252 | M | L | L | H |
| Senegal | 2.06 | 0.25 | 1.93 | 0.27 | 7,950,893 | M | M | H | L |
| Serbia and Montenegro | 1.84 | 0.07 | 1.66 | 0.03 | 260,022 | M | M | L | M |
| Seychelles | 1.47 | 0.02 | 1.20 | 0.04 | 123,837 | M | L | H | M |
| Sierra Leone | 1.63 | 0.08 | 1.48 | 0.12 | 3,175,642 | M | M | H | L |
| Singapore | 1.40 | 0.00 | 1.18 | 0.00 | 6,174,908 | M | L | M | H |
| Slovenia | 2.18 | 0.00 | 1.88 | 0.00 | 347,970 | M | M | L | H |
| Solomon Islands | 1.33 | 0.03 | 1.07 | 0.01 | 879,806 | M | L | H | L |
| Somalia | 1.68 | 0.08 | 1.38 | 0.06 | 7,926,014 | M | M | L | L |
| South Korea | 2.23 | 0.22 | 2.05 | 0.23 | 38,119,966 | M | M | M | H |
| Sri Lanka | 1.63 | 0.06 | 1.37 | 0.08 | 19,538,921 | M | M | H | L |
| Sudan | 1.40 | 0.01 | 1.10 | 0.02 | 964,075 | M | L | L | L |
| Suriname | 1.69 | 0.11 | 1.45 | 0.11 | 480,783 | M | M | L | M |
| Sweden | 2.87 | 0.09 | 2.62 | 0.11 | 3,976,660 | H | H | L | H |
| Syria | 1.65 | 0.01 | 1.43 | 0.01 | 4,154,032 | M | M | L | L |
| Taiwan | 1.44 | 0.05 | 1.24 | 0.07 | 27,421,100 | M | L |  |  |
| Tanzania | 1.46 | 0.03 | 1.20 | 0.03 | 9,022,265 | M | L | M | L |
| Thailand | 1.35 | 0.02 | 1.15 | 0.06 | 32,224,400 | M | L | M | L |
| Togo | 1.82 | 0.12 | 1.66 | 0.17 | 2,767,901 | M | M | M | L |
| Tonga | 1.34 | 0.05 | 1.05 | 0.06 | 133,259 | M | L | H | L |
| Trinidad and Tobago | 1.58 | 0.14 | 1.39 | 0.18 | 1,720,359 | M | M | L | H |
| Tunisia | 1.62 | 0.02 | 1.37 | 0.05 | 7,202,610 | M | M | M | L |
| Turkey | 1.69 | 0.05 | 1.47 | 0.04 | 34,610,765 | M | M | L | M |
| Tuvalu | 1.24 | 0.04 | 0.99 | 0.05 | 18,006 | L | L | H | L |
| United Arab Emirates | 1.68 | 0.05 | 1.35 | 0.05 | 2,442,252 | M | M | L | H |
| Uruguay | 2.10 | 0.20 | 1.98 | 0.22 | 2,330,661 | M | M | L | M |
| USA | 2.00 | 0.47 | 1.76 | 0.49 | 124,387,013 | M | M | L | H |
| Vanuatu | 1.36 | 0.02 | 1.07 | 0.02 | 323,457 | M | L | H | L |
| Venezuela | 1.77 | 0.30 | 1.63 | 0.36 | 20,602,213 | M | M | L | M |
| Vietnam | 1.42 | 0.03 | 1.16 | 0.05 | 51,903,256 | M | L | M | L |
| Yemen | 1.81 | 0.20 | 1.46 | 0.21 | 7,890,628 | M | M | L | L |

| **Temperature (^o^C)** | **rcp85** | | **rcp45** | | **Accuracy** | | **Precision** | |
| --- | --- | --- | --- | --- | --- | --- | --- | --- |
|  | **MEAN** | **STD** | **MEAN** | **STD** | **MEAN** | **STD** | **MEAN** | **STD** |
| Albania | 3.87 | 0.07 | 1.81 | 0.04 | -0.30 | 0.00 | 1.05 | 0.04 |
| Algeria | 3.32 | 0.09 | 1.49 | 0.05 | -0.16 | 0.11 | 0.85 | 0.08 |
| American Samoa | 2.96 | 0.21 | 1.30 | 0.11 | -0.49 | 0.18 | 0.81 | 0.18 |
| Angola | 3.21 | 0.07 | 1.43 | 0.03 | 3.12 | 1.10 | 1.20 | 0.11 |
| Anguilla | 2.96 | 0.11 | 1.35 | 0.07 | -0.30 | 0.43 | 0.81 | 0.08 |
| Antigua and Barbuda | 2.77 | 0.02 | 1.23 | 0.01 | -1.08 | 0.08 | 0.75 | 0.01 |
| Argentina | 2.58 | 0.56 | 1.14 | 0.22 | 0.24 | 1.04 | 1.49 | 0.63 |
| Australia | 2.91 | 0.34 | 1.24 | 0.12 | -0.23 | 0.45 | 0.83 | 0.39 |
| Bahamas | 2.92 | 0.08 | 1.32 | 0.04 | -0.19 | 0.18 | 0.75 | 0.06 |
| Bahrain | 3.65 | 0.01 | 1.60 | 0.01 | -1.97 | 0.00 | 1.74 | 0.01 |
| Bangladesh | 2.98 | 0.03 | 1.41 | 0.02 | 0.06 | 0.09 | 0.82 | 0.06 |
| Barbados | 2.80 | 0.03 | 1.22 | 0.01 | -1.01 | 0.09 | 0.74 | 0.05 |
| Belgium | 3.23 | 0.00 | 1.39 | 0.01 |  |  |  |  |
| Belize | 2.92 | 0.01 | 1.32 | 0.01 | -1.02 | 0.01 | 0.76 | 0.03 |
| Benin | 3.14 | 0.01 | 1.41 | 0.01 | 0.52 | 0.04 | 0.78 | 0.02 |
| Bosnia and Herzegovina | 4.03 | 0.00 | 1.93 | 0.00 |  |  |  |  |
| Bouvet Island | 3.09 | 0.89 | 1.43 | 0.52 | -0.37 | 0.92 | 1.88 | 0.69 |
| Brazil | 2.87 | 0.25 | 1.26 | 0.11 | -0.57 | 0.43 | 0.92 | 0.14 |
| Cambodia | 2.89 | 0.02 | 1.30 | 0.01 | -0.28 | 0.03 | 0.66 | 0.03 |
| Cameroon | 3.10 | 0.01 | 1.41 | 0.00 |  |  |  |  |
| Canada | 2.96 | 1.00 | 1.09 | 0.60 | 0.43 | 1.43 | 1.20 | 0.65 |
| Canary Islands | 2.76 | 0.41 | 1.21 | 0.20 | 0.05 | 0.38 | 1.03 | 0.16 |
| Cape Verde | 3.07 | 0.09 | 1.41 | 0.04 | 0.16 | 0.33 | 0.91 | 0.12 |
| Chile | 2.15 | 0.43 | 1.00 | 0.21 | 0.40 | 0.91 | 1.07 | 0.38 |
| China | 3.48 | 0.58 | 1.66 | 0.28 | 0.67 | 0.59 | 1.37 | 0.58 |
| Colombia | 3.02 | 0.10 | 1.36 | 0.04 | -0.59 | 0.92 | 0.99 | 0.24 |
| Comoros | 2.98 | 0.02 | 1.29 | 0.01 | -0.05 | 0.09 | 0.75 | 0.05 |
| Cook Islands | 2.60 | 0.41 | 1.13 | 0.18 | -0.31 | 0.53 | 0.91 | 0.35 |
| Costa Rica | 3.09 | 0.06 | 1.38 | 0.03 | 0.13 | 0.32 | 0.75 | 0.08 |
| Croatia | 4.03 | 0.03 | 1.89 | 0.03 | -0.94 | 0.25 | 1.15 | 0.05 |
| Cuba | 2.93 | 0.03 | 1.32 | 0.01 | -0.52 | 0.22 | 0.73 | 0.04 |
| Cyprus | 3.67 | 0.05 | 1.66 | 0.03 | -0.47 | 0.17 | 0.95 | 0.21 |
| Democratic Republic of the Congo | 3.14 | 0.02 | 1.40 | 0.01 | 1.81 | 0.00 | 1.20 | 0.00 |
| Denmark | 2.01 | 0.67 | 0.62 | 0.48 | -0.09 | 0.88 | 1.54 | 0.93 |
| Djibouti | 3.38 | 0.02 | 1.44 | 0.01 | -1.44 | 0.00 | 1.69 | 0.06 |
| Dominica | 2.75 | 0.01 | 1.20 | 0.01 | -1.14 | 0.06 | 0.77 | 0.03 |
| Dominican Republic | 2.82 | 0.03 | 1.25 | 0.03 | -0.94 | 0.22 | 0.76 | 0.04 |
| East Timor | 2.98 | 0.03 | 1.28 | 0.01 | 0.06 | 0.09 | 0.57 | 0.05 |
| Ecuador | 3.37 | 0.16 | 1.51 | 0.07 | 1.63 | 1.03 | 0.95 | 0.16 |
| Egypt | 3.49 | 0.07 | 1.54 | 0.06 | -0.28 | 0.45 | 1.04 | 0.43 |
| El Salvador | 3.11 | 0.02 | 1.41 | 0.01 | -0.01 | 0.17 | 0.98 | 0.07 |
| Equatorial Guinea | 3.22 | 0.05 | 1.42 | 0.01 | 1.56 | 0.43 | 1.09 | 0.10 |
| Eritrea | 3.42 | 0.02 | 1.45 | 0.01 | -2.15 | 0.02 | 2.08 | 0.10 |
| Estonia | 4.22 | 0.03 | 1.96 | 0.02 | 0.42 | 0.06 | 1.57 | 0.07 |
| Fiji | 2.68 | 0.07 | 1.13 | 0.03 | -0.35 | 0.14 | 0.55 | 0.04 |
| Finland | 4.15 | 0.07 | 1.93 | 0.06 | 0.05 | 0.29 | 1.54 | 0.07 |
| France | 2.92 | 0.42 | 1.29 | 0.26 | 0.51 | 0.25 | 0.91 | 0.06 |
| French Guiana | 2.94 | 0.05 | 1.27 | 0.03 | -0.55 | 0.11 | 0.74 | 0.09 |
| French Polynesia | 2.40 | 0.19 | 1.03 | 0.09 | -0.45 | 0.36 | 0.68 | 0.22 |
| Gabon | 3.21 | 0.03 | 1.42 | 0.01 | 2.10 | 0.28 | 1.19 | 0.10 |
| Gambia | 3.12 | 0.02 | 1.45 | 0.01 | 1.18 | 0.35 | 1.05 | 0.12 |
| Germany | 3.39 | 0.24 | 1.48 | 0.14 | 0.07 | 0.09 | 1.36 | 0.04 |
| Ghana | 3.11 | 0.03 | 1.39 | 0.01 | 0.55 | 0.11 | 0.81 | 0.04 |
| Gibraltar | 3.02 | 0.02 | 1.31 | 0.01 |  |  |  |  |
| Greece | 3.63 | 0.14 | 1.63 | 0.08 | -0.22 | 0.16 | 0.83 | 0.13 |
| Grenada | 2.84 | 0.04 | 1.26 | 0.03 | -1.17 | 0.12 | 0.86 | 0.11 |
| Guadeloupe | 2.75 | 0.01 | 1.21 | 0.01 | -1.18 | 0.06 | 0.77 | 0.02 |
| Guatemala | 3.15 | 0.02 | 1.41 | 0.01 | -0.23 | 0.21 | 0.96 | 0.06 |
| Guernsey | 2.40 | 0.52 | 0.94 | 0.29 | 0.32 | 0.39 | 1.47 | 0.29 |
| Guinea | 3.02 | 0.03 | 1.36 | 0.01 | 0.63 | 0.12 | 0.80 | 0.09 |
| Guinea-Bissau | 3.07 | 0.02 | 1.39 | 0.01 | 0.96 | 0.23 | 0.83 | 0.13 |
| Guyana | 2.92 | 0.03 | 1.28 | 0.02 | -1.00 | 0.11 | 0.67 | 0.04 |
| Haiti | 2.86 | 0.03 | 1.27 | 0.02 | -1.03 | 0.11 | 0.80 | 0.02 |
| Honduras | 2.91 | 0.02 | 1.31 | 0.01 | -1.05 | 0.16 | 0.77 | 0.03 |
| Iceland | 1.88 | 0.68 | 0.50 | 0.40 | 0.25 | 0.85 | 2.39 | 0.40 |
| India | 3.04 | 0.13 | 1.38 | 0.05 | -0.36 | 0.28 | 0.61 | 0.09 |
| Indonesia | 2.89 | 0.08 | 1.26 | 0.04 | 0.27 | 0.22 | 0.63 | 0.08 |
| Iran | 3.49 | 0.19 | 1.54 | 0.08 | -1.37 | 0.32 | 1.45 | 0.47 |
| Iraq | 3.77 | 0.01 | 1.65 | 0.01 |  |  | 1.86 | 0.00 |
| Ireland | 2.06 | 0.25 | 0.74 | 0.14 | 0.34 | 0.15 | 1.24 | 0.15 |
| Israel | 3.62 | 0.03 | 1.61 | 0.02 | -0.66 | 0.17 | 0.98 | 0.05 |
| Italy | 3.62 | 0.12 | 1.65 | 0.08 | -0.30 | 0.31 | 0.84 | 0.11 |
| Ivory Coast | 3.11 | 0.05 | 1.39 | 0.02 | 0.60 | 0.20 | 0.86 | 0.06 |
| Jamaica | 2.87 | 0.03 | 1.28 | 0.02 | -1.20 | 0.16 | 0.77 | 0.02 |
| Japan | 3.28 | 0.46 | 1.55 | 0.20 | 0.05 | 0.94 | 1.18 | 0.63 |
| Jordan | 3.54 | 0.00 | 1.54 | 0.00 |  |  |  |  |
| Kenya | 3.13 | 0.05 | 1.37 | 0.02 | 0.07 | 0.07 | 0.60 | 0.01 |
| Kiribati | 2.94 | 0.25 | 1.31 | 0.13 | -0.14 | 0.32 | 0.97 | 0.12 |
| Kuwait | 3.76 | 0.02 | 1.64 | 0.01 | -1.52 | 0.00 | 1.87 | 0.03 |
| Latvia | 4.16 | 0.05 | 1.94 | 0.02 | 0.77 | 0.05 | 1.51 | 0.07 |
| Lebanon | 3.71 | 0.03 | 1.67 | 0.02 | -0.92 | 0.03 | 1.09 | 0.05 |
| Liberia | 3.10 | 0.07 | 1.38 | 0.03 | 0.50 | 0.27 | 0.82 | 0.08 |
| Libya | 3.50 | 0.05 | 1.56 | 0.02 | -0.10 | 0.18 | 0.81 | 0.13 |
| Lithuania | 4.11 | 0.03 | 1.93 | 0.01 | 0.46 | 0.00 | 1.44 | 0.04 |
| Madagascar | 2.90 | 0.10 | 1.28 | 0.06 | -0.28 | 0.38 | 0.75 | 0.10 |
| Malaysia | 2.91 | 0.03 | 1.32 | 0.02 | 0.26 | 0.23 | 0.74 | 0.13 |
| Maldives | 3.01 | 0.09 | 1.35 | 0.05 | 0.05 | 0.07 | 0.52 | 0.02 |
| Malta | 3.56 | 0.02 | 1.58 | 0.01 | -0.12 | 0.12 | 0.76 | 0.03 |
| Marshall Islands | 2.97 | 0.08 | 1.27 | 0.04 | -0.04 | 0.27 | 0.77 | 0.06 |
| Mauritania | 3.19 | 0.04 | 1.49 | 0.01 | 0.93 | 0.52 | 1.45 | 0.22 |
| Mauritius | 2.82 | 0.09 | 1.20 | 0.05 | -0.14 | 0.11 | 0.76 | 0.07 |
| Mexico | 3.00 | 0.10 | 1.38 | 0.07 | 0.97 | 1.19 | 1.01 | 0.27 |
| Micronesia | 2.94 | 0.05 | 1.26 | 0.04 | 0.06 | 0.23 | 0.69 | 0.06 |
| Monaco | 3.57 | 0.01 | 1.70 | 0.00 | 0.38 | 0.00 | 0.88 | 0.00 |
| Morocco | 2.72 | 0.20 | 1.22 | 0.08 | 0.30 | 0.48 | 1.19 | 0.14 |
| Mozambique | 2.96 | 0.05 | 1.29 | 0.02 | 0.30 | 0.25 | 0.69 | 0.05 |
| Myanmar | 2.88 | 0.05 | 1.34 | 0.03 | -0.37 | 0.17 | 0.66 | 0.06 |
| Namibia | 3.11 | 0.25 | 1.37 | 0.13 | 3.31 | 1.67 | 1.42 | 0.15 |
| Nauru | 3.07 | 0.07 | 1.40 | 0.04 | -0.44 | 0.24 | 0.99 | 0.07 |
| Netherlands | 3.15 | 0.07 | 1.34 | 0.04 | 0.28 | 0.16 | 1.32 | 0.04 |
| Netherlands Antilles | 2.89 | 0.08 | 1.30 | 0.06 | -1.37 | 0.18 | 0.85 | 0.10 |
| New Caledonia | 2.86 | 0.07 | 1.18 | 0.03 | -0.27 | 0.12 | 0.54 | 0.04 |
| Nicaragua | 2.96 | 0.08 | 1.34 | 0.04 | -0.41 | 0.78 | 0.85 | 0.08 |
| Nigeria | 3.15 | 0.01 | 1.42 | 0.01 | 0.46 | 0.11 | 0.79 | 0.04 |
| North Korea | 4.39 | 0.08 | 2.18 | 0.06 | 1.84 | 0.56 | 2.45 | 0.22 |
| Oman | 3.29 | 0.10 | 1.43 | 0.04 | -0.76 | 0.20 | 0.72 | 0.13 |
| Pakistan | 3.21 | 0.03 | 1.44 | 0.02 | -0.91 | 0.13 | 0.79 | 0.04 |
| Palau | 2.94 | 0.05 | 1.27 | 0.03 | 0.34 | 0.20 | 0.62 | 0.02 |
| Panama | 3.06 | 0.10 | 1.37 | 0.04 | -0.27 | 0.58 | 0.94 | 0.18 |
| Papua New Guinea | 2.84 | 0.07 | 1.22 | 0.06 | -0.11 | 0.15 | 0.62 | 0.10 |
| Peru | 3.34 | 0.11 | 1.52 | 0.05 | 3.14 | 0.80 | 1.48 | 0.13 |
| Philippines | 2.95 | 0.06 | 1.32 | 0.02 | -0.03 | 0.29 | 0.68 | 0.07 |
| Poland | 3.95 | 0.07 | 1.83 | 0.05 | 0.77 | 0.13 | 1.51 | 0.04 |
| Prince Edward Islands | 2.62 | 0.25 | 1.21 | 0.14 | 0.48 | 0.52 | 1.17 | 0.21 |
| Puerto Rico | 2.80 | 0.03 | 1.24 | 0.02 | -1.00 | 0.20 | 0.75 | 0.04 |
| Qatar | 3.61 | 0.02 | 1.59 | 0.01 | -1.76 | 0.01 | 1.77 | 0.03 |
| Russia | 3.60 | 1.03 | 1.57 | 0.61 | -0.41 | 0.84 | 1.07 | 0.66 |
| Saint Kitts and Nevis | 2.75 | 0.01 | 1.22 | 0.01 | -1.15 | 0.00 | 0.76 | 0.01 |
| Saint Lucia | 2.77 | 0.01 | 1.19 | 0.01 | -1.02 | 0.01 | 0.71 | 0.03 |
| Saint Pierre and Miquelon | 4.15 | 0.18 | 1.93 | 0.08 | 2.93 | 0.24 | 2.17 | 0.20 |
| Saint Vincent and the Grenadines | 2.79 | 0.02 | 1.21 | 0.02 | -0.99 | 0.05 | 0.71 | 0.04 |
| Samoa | 2.60 | 0.01 | 1.13 | 0.01 | -0.30 | 0.08 | 0.56 | 0.04 |
| Sao Tome and Principe | 3.18 | 0.03 | 1.41 | 0.01 | 1.07 | 0.28 | 0.92 | 0.08 |
| Saudi Arabia | 3.47 | 0.11 | 1.48 | 0.07 | -1.79 | 0.42 | 1.97 | 0.17 |
| Senegal | 3.13 | 0.05 | 1.45 | 0.03 | 0.97 | 0.36 | 1.05 | 0.23 |
| Serbia and Montenegro | 3.96 | 0.05 | 1.88 | 0.03 | -0.30 | 0.00 | 1.07 | 0.00 |
| Seychelles | 3.08 | 0.07 | 1.35 | 0.04 | 0.24 | 0.14 | 0.61 | 0.04 |
| Sierra Leone | 3.03 | 0.03 | 1.35 | 0.01 | 0.37 | 0.15 | 0.75 | 0.05 |
| Singapore | 2.91 | 0.01 | 1.35 | 0.01 |  |  |  |  |
| Slovenia | 4.02 | 0.00 | 1.84 | 0.00 |  |  |  |  |
| Solomon Islands | 2.72 | 0.05 | 1.17 | 0.01 | -0.09 | 0.13 | 0.57 | 0.05 |
| Somalia | 3.30 | 0.07 | 1.43 | 0.02 | 0.11 | 0.64 | 0.65 | 0.14 |
| South Korea | 3.94 | 0.26 | 1.90 | 0.14 | 1.04 | 0.34 | 2.07 | 0.22 |
| Sri Lanka | 3.03 | 0.03 | 1.37 | 0.01 | -0.11 | 0.24 | 0.51 | 0.02 |
| Sudan | 3.37 | 0.01 | 1.42 | 0.01 | -2.02 | 0.13 | 1.97 | 0.10 |
| Suriname | 2.93 | 0.04 | 1.28 | 0.03 | -0.81 | 0.13 | 0.77 | 0.12 |
| Sweden | 4.02 | 0.16 | 1.86 | 0.09 | 0.47 | 0.56 | 1.50 | 0.05 |
| Syria | 3.76 | 0.01 | 1.71 | 0.01 |  |  | 1.34 | 0.02 |
| Taiwan | 2.88 | 0.06 | 1.36 | 0.04 | 0.35 | 0.29 | 0.75 | 0.09 |
| Tanzania | 3.03 | 0.05 | 1.33 | 0.02 | -0.02 | 0.09 | 0.64 | 0.03 |
| Thailand | 2.88 | 0.02 | 1.31 | 0.01 | -0.17 | 0.11 | 0.65 | 0.06 |
| Togo | 3.12 | 0.02 | 1.40 | 0.01 | 0.53 | 0.05 | 0.77 | 0.01 |
| Tonga | 2.66 | 0.08 | 1.12 | 0.04 | -0.40 | 0.12 | 0.54 | 0.07 |
| Trinidad and Tobago | 2.86 | 0.03 | 1.25 | 0.03 | -0.96 | 0.07 | 0.69 | 0.05 |
| Tunisia | 3.56 | 0.04 | 1.59 | 0.01 | 0.01 | 0.03 | 0.80 | 0.04 |
| Turkey | 3.74 | 0.05 | 1.69 | 0.03 | -0.48 | 0.19 | 1.29 | 0.31 |
| Tuvalu | 2.67 | 0.06 | 1.17 | 0.03 | 0.05 | 0.11 | 0.71 | 0.08 |
| United Arab Emirates | 3.54 | 0.04 | 1.56 | 0.01 | -1.66 | 0.00 | 1.73 | 0.09 |
| Uruguay | 3.01 | 0.14 | 1.47 | 0.10 | 0.92 | 0.63 | 1.47 | 0.32 |
| USA | 3.54 | 0.54 | 1.64 | 0.32 | 0.19 | 1.14 | 1.23 | 0.37 |
| Vanuatu | 2.73 | 0.03 | 1.14 | 0.02 | -0.39 | 0.13 | 0.54 | 0.02 |
| Venezuela | 2.89 | 0.10 | 1.30 | 0.07 | -1.31 | 0.25 | 0.88 | 0.15 |
| Vietnam | 2.87 | 0.07 | 1.30 | 0.05 | 0.02 | 0.17 | 0.66 | 0.11 |
| Yemen | 3.42 | 0.05 | 1.47 | 0.03 | -0.45 | 0.48 | 0.72 | 0.32 |

| **Oxygen (ml/L)** | **rcp85** | | **Rcp45** | | **Accuracy** | | **Precision** | |
| --- | --- | --- | --- | --- | --- | --- | --- | --- |
|  | **MEAN** | **STD** | **MEAN** | **STD** | **MEAN** | **STD** | **MEAN** | **STD** |
| Albania | -0.36 | 0.01 | -0.18 | 0.01 | 0.00 | 0.00 | 0.14 | 0.01 |
| Algeria | -0.29 | 0.01 | -0.14 | 0.01 | -0.02 | 0.06 | 0.16 | 0.02 |
| American Samoa | -0.21 | 0.02 | -0.09 | 0.01 | 0.02 | 0.02 | 0.09 | 0.02 |
| Angola | -0.23 | 0.01 | -0.11 | 0.01 | -0.23 | 0.05 | 0.11 | 0.02 |
| Anguilla | -0.23 | 0.01 | -0.11 | 0.01 | -0.01 | 0.04 | 0.09 | 0.01 |
| Antigua and Barbuda | -0.21 | 0.00 | -0.09 | 0.00 | 0.03 | 0.01 | 0.12 | 0.00 |
| Argentina | -0.32 | 0.07 | -0.14 | 0.03 | -0.05 | 0.20 | 0.32 | 0.09 |
| Australia | -0.24 | 0.06 | -0.10 | 0.02 | 0.06 | 0.07 | 0.13 | 0.08 |
| Bahamas | -0.23 | 0.01 | -0.10 | 0.01 | -0.02 | 0.03 | 0.08 | 0.01 |
| Bahrain | -0.27 | 0.00 | -0.12 | 0.00 | -0.12 | 0.00 | 0.36 | 0.01 |
| Bangladesh | -0.20 | 0.01 | -0.10 | 0.00 | -0.13 | 0.02 | 0.10 | 0.01 |
| Barbados | -0.21 | 0.00 | -0.09 | 0.00 | 0.05 | 0.01 | 0.11 | 0.01 |
| Belgium | -0.36 | 0.01 | -0.20 | 0.00 | 0.00 | 0.00 | 0.00 | 0.00 |
| Belize | -0.22 | 0.00 | -0.10 | 0.00 | 0.21 | 0.01 | 0.11 | 0.00 |
| Benin | -0.24 | 0.00 | -0.11 | 0.00 | -0.31 | 0.09 | 0.06 | 0.00 |
| Bosnia and Herzegovina | -0.38 | 0.00 | -0.20 | 0.00 | 0.00 | 0.00 | 0.00 | 0.00 |
| Bouvet Island | -0.46 | 0.17 | -0.26 | 0.13 | 0.02 | 0.15 | 0.47 | 0.12 |
| Brazil | -0.23 | 0.02 | -0.10 | 0.01 | 0.04 | 0.11 | 0.10 | 0.02 |
| Cambodia | -0.18 | 0.00 | -0.08 | 0.00 | 0.11 | 0.02 | 0.17 | 0.03 |
| Cameroon | -0.22 | 0.00 | -0.10 | 0.00 | 0.00 | 0.00 | 0.00 | 0.00 |
| Canada | -0.34 | 0.16 | -0.13 | 0.10 | -0.20 | 0.32 | 0.34 | 0.09 |
| Canary Islands | -0.24 | 0.04 | -0.11 | 0.02 | 0.00 | 0.06 | 0.19 | 0.04 |
| Cape Verde | -0.24 | 0.01 | -0.11 | 0.00 | -0.08 | 0.02 | 0.11 | 0.02 |
| Chile | -0.21 | 0.04 | -0.10 | 0.03 | -0.03 | 0.13 | 0.21 | 0.12 |
| China | -0.31 | 0.12 | -0.15 | 0.06 | -0.12 | 0.11 | 0.20 | 0.10 |
| Colombia | -0.22 | 0.01 | -0.10 | 0.01 | 0.13 | 0.05 | 0.11 | 0.01 |
| Comoros | -0.21 | 0.00 | -0.09 | 0.00 | -0.04 | 0.02 | 0.06 | 0.01 |
| Cook Islands | -0.24 | 0.06 | -0.10 | 0.03 | 0.01 | 0.10 | 0.16 | 0.09 |
| Costa Rica | -0.20 | 0.01 | -0.09 | 0.00 | 0.00 | 0.05 | 0.10 | 0.01 |
| Croatia | -0.40 | 0.02 | -0.21 | 0.01 | 0.07 | 0.03 | 0.17 | 0.02 |
| Cuba | -0.22 | 0.00 | -0.10 | 0.00 | 0.04 | 0.04 | 0.09 | 0.01 |
| Cyprus | -0.31 | 0.01 | -0.16 | 0.01 | 0.03 | 0.04 | 0.13 | 0.01 |
| Democratic Republic of the Congo | -0.23 | 0.01 | -0.11 | 0.00 | -0.31 | 0.00 | 0.15 | 0.01 |
| Denmark | -0.25 | 0.10 | -0.12 | 0.07 | -0.06 | 0.22 | 0.39 | 0.11 |
| Djibouti | -0.24 | 0.00 | -0.10 | 0.00 | 0.05 | 0.00 | 0.14 | 0.00 |
| Dominica | -0.21 | 0.00 | -0.09 | 0.00 | 0.05 | 0.01 | 0.12 | 0.00 |
| Dominican Republic | -0.22 | 0.00 | -0.09 | 0.00 | 0.08 | 0.06 | 0.11 | 0.01 |
| East Timor | -0.20 | 0.00 | -0.09 | 0.00 | 0.04 | 0.02 | 0.11 | 0.03 |
| Ecuador | -0.23 | 0.01 | -0.10 | 0.01 | 0.07 | 0.06 | 0.13 | 0.03 |
| Egypt | -0.27 | 0.02 | -0.13 | 0.01 | 0.03 | 0.07 | 0.15 | 0.02 |
| El Salvador | -0.21 | 0.00 | -0.10 | 0.00 | -0.06 | 0.07 | 0.09 | 0.01 |
| Equatorial Guinea | -0.23 | 0.01 | -0.11 | 0.00 | -0.07 | 0.04 | 0.10 | 0.02 |
| Eritrea | -0.24 | 0.00 | -0.10 | 0.00 | 0.11 | 0.01 | 0.19 | 0.02 |
| Estonia | -0.65 | 0.04 | -0.34 | 0.02 | -0.28 | 0.03 | 0.65 | 0.06 |
| Fiji | -0.20 | 0.01 | -0.08 | 0.01 | 0.01 | 0.03 | 0.09 | 0.00 |
| Finland | -0.67 | 0.04 | -0.35 | 0.03 | -0.31 | 0.07 | 0.70 | 0.04 |
| France | -0.27 | 0.04 | -0.14 | 0.03 | 0.00 | 0.10 | 0.19 | 0.03 |
| French Guiana | -0.22 | 0.00 | -0.10 | 0.00 | -0.03 | 0.01 | 0.08 | 0.00 |
| French Polynesia | -0.18 | 0.02 | -0.08 | 0.01 | 0.00 | 0.04 | 0.08 | 0.01 |
| Gabon | -0.23 | 0.00 | -0.11 | 0.00 | -0.13 | 0.07 | 0.16 | 0.04 |
| Gambia | -0.25 | 0.00 | -0.11 | 0.00 | -0.11 | 0.01 | 0.10 | 0.01 |
| Germany | -0.44 | 0.08 | -0.24 | 0.03 | 0.14 | 0.03 | 0.46 | 0.07 |
| Ghana | -0.24 | 0.00 | -0.10 | 0.00 | -0.10 | 0.08 | 0.06 | 0.01 |
| Gibraltar | -0.25 | 0.00 | -0.11 | 0.00 | 0.00 | 0.00 | 0.00 | 0.00 |
| Greece | -0.34 | 0.03 | -0.17 | 0.02 | -0.01 | 0.04 | 0.14 | 0.03 |
| Grenada | -0.21 | 0.00 | -0.09 | 0.00 | 0.15 | 0.03 | 0.09 | 0.01 |
| Guadeloupe | -0.21 | 0.00 | -0.09 | 0.00 | 0.05 | 0.01 | 0.12 | 0.00 |
| Guatemala | -0.22 | 0.00 | -0.10 | 0.00 | 0.00 | 0.01 | 0.07 | 0.00 |
| Guernsey | -0.27 | 0.07 | -0.15 | 0.04 | 0.06 | 0.14 | 0.35 | 0.08 |
| Guinea | -0.24 | 0.00 | -0.11 | 0.00 | -0.07 | 0.01 | 0.07 | 0.01 |
| Guinea-Bissau | -0.24 | 0.00 | -0.11 | 0.00 | -0.10 | 0.02 | 0.08 | 0.01 |
| Guyana | -0.22 | 0.00 | -0.09 | 0.00 | 0.12 | 0.04 | 0.08 | 0.01 |
| Haiti | -0.22 | 0.00 | -0.09 | 0.00 | 0.12 | 0.02 | 0.11 | 0.01 |
| Honduras | -0.22 | 0.00 | -0.10 | 0.00 | 0.10 | 0.03 | 0.11 | 0.01 |
| Iceland | -0.28 | 0.12 | -0.14 | 0.07 | -0.05 | 0.12 | 0.50 | 0.07 |
| India | -0.21 | 0.01 | -0.09 | 0.01 | 0.03 | 0.05 | 0.07 | 0.01 |
| Indonesia | -0.19 | 0.01 | -0.08 | 0.00 | 0.08 | 0.09 | 0.11 | 0.03 |
| Iran | -0.25 | 0.02 | -0.11 | 0.01 | -0.04 | 0.10 | 0.26 | 0.14 |
| Iraq | -0.28 | 0.00 | -0.12 | 0.00 | 0.00 | 0.00 | 0.40 | 0.00 |
| Ireland | -0.18 | 0.03 | -0.08 | 0.02 | -0.04 | 0.08 | 0.26 | 0.03 |
| Israel | -0.29 | 0.01 | -0.14 | 0.01 | 0.13 | 0.03 | 0.14 | 0.01 |
| Italy | -0.33 | 0.02 | -0.16 | 0.02 | 0.05 | 0.07 | 0.13 | 0.01 |
| Ivory Coast | -0.24 | 0.00 | -0.10 | 0.00 | -0.02 | 0.04 | 0.07 | 0.00 |
| Jamaica | -0.22 | 0.00 | -0.10 | 0.00 | 0.13 | 0.03 | 0.11 | 0.01 |
| Japan | -0.26 | 0.09 | -0.13 | 0.04 | -0.05 | 0.16 | 0.14 | 0.11 |
| Jordan | -0.27 | 0.00 | -0.12 | 0.00 | 0.00 | 0.00 | 0.00 | 0.00 |
| Kenya | -0.22 | 0.00 | -0.10 | 0.00 | -0.14 | 0.03 | 0.06 | 0.00 |
| Kiribati | -0.20 | 0.02 | -0.09 | 0.01 | 0.00 | 0.07 | 0.09 | 0.04 |
| Kuwait | -0.28 | 0.00 | -0.12 | 0.00 | -0.42 | 0.00 | 0.40 | 0.01 |
| Latvia | -0.66 | 0.02 | -0.35 | 0.01 | -0.42 | 0.04 | 0.69 | 0.07 |
| Lebanon | -0.31 | 0.00 | -0.15 | 0.00 | 0.18 | 0.01 | 0.13 | 0.00 |
| Liberia | -0.23 | 0.00 | -0.10 | 0.00 | -0.04 | 0.03 | 0.07 | 0.00 |
| Libya | -0.30 | 0.01 | -0.14 | 0.00 | -0.02 | 0.04 | 0.13 | 0.01 |
| Lithuania | -0.66 | 0.01 | -0.36 | 0.00 | -0.34 | 0.00 | 0.69 | 0.01 |
| Madagascar | -0.21 | 0.01 | -0.10 | 0.01 | -0.06 | 0.07 | 0.08 | 0.01 |
| Malaysia | -0.19 | 0.00 | -0.09 | 0.00 | 0.12 | 0.05 | 0.13 | 0.02 |
| Maldives | -0.21 | 0.00 | -0.09 | 0.00 | 0.05 | 0.04 | 0.06 | 0.00 |
| Malta | -0.31 | 0.00 | -0.14 | 0.00 | 0.00 | 0.01 | 0.13 | 0.00 |
| Marshall Islands | -0.20 | 0.01 | -0.09 | 0.00 | -0.02 | 0.05 | 0.08 | 0.01 |
| Mauritania | -0.27 | 0.00 | -0.12 | 0.00 | -0.04 | 0.05 | 0.17 | 0.02 |
| Mauritius | -0.20 | 0.00 | -0.08 | 0.00 | 0.02 | 0.02 | 0.05 | 0.01 |
| Mexico | -0.22 | 0.02 | -0.10 | 0.01 | -0.07 | 0.13 | 0.10 | 0.03 |
| Micronesia | -0.20 | 0.01 | -0.08 | 0.01 | -0.02 | 0.04 | 0.08 | 0.00 |
| Monaco | -0.33 | 0.00 | -0.18 | 0.00 | 0.18 | 0.00 | 0.14 | 0.00 |
| Morocco | -0.24 | 0.02 | -0.12 | 0.01 | -0.05 | 0.08 | 0.26 | 0.03 |
| Mozambique | -0.21 | 0.01 | -0.09 | 0.00 | -0.09 | 0.06 | 0.06 | 0.01 |
| Myanmar | -0.19 | 0.01 | -0.09 | 0.00 | -0.04 | 0.04 | 0.09 | 0.02 |
| Namibia | -0.27 | 0.02 | -0.13 | 0.01 | -0.40 | 0.17 | 0.18 | 0.04 |
| Nauru | -0.22 | 0.01 | -0.10 | 0.00 | 0.03 | 0.03 | 0.09 | 0.01 |
| Netherlands | -0.37 | 0.01 | -0.20 | 0.01 | 0.18 | 0.03 | 0.39 | 0.02 |
| Netherlands Antilles | -0.22 | 0.01 | -0.10 | 0.01 | 0.10 | 0.02 | 0.11 | 0.01 |
| New Caledonia | -0.21 | 0.01 | -0.09 | 0.01 | 0.02 | 0.03 | 0.08 | 0.00 |
| Nicaragua | -0.22 | 0.01 | -0.10 | 0.00 | -0.04 | 0.17 | 0.11 | 0.00 |
| Nigeria | -0.23 | 0.00 | -0.10 | 0.00 | -0.15 | 0.08 | 0.07 | 0.01 |
| North Korea | -0.49 | 0.02 | -0.26 | 0.01 | -0.36 | 0.09 | 0.29 | 0.02 |
| Oman | -0.24 | 0.01 | -0.11 | 0.00 | 0.03 | 0.05 | 0.07 | 0.02 |
| Pakistan | -0.23 | 0.00 | -0.10 | 0.00 | 0.04 | 0.04 | 0.09 | 0.01 |
| Palau | -0.20 | 0.00 | -0.08 | 0.00 | -0.07 | 0.02 | 0.08 | 0.00 |
| Panama | -0.21 | 0.01 | -0.09 | 0.01 | 0.06 | 0.05 | 0.10 | 0.01 |
| Papua New Guinea | -0.20 | 0.01 | -0.09 | 0.00 | -0.03 | 0.04 | 0.08 | 0.01 |
| Peru | -0.24 | 0.01 | -0.11 | 0.01 | -0.11 | 0.06 | 0.20 | 0.08 |
| Philippines | -0.20 | 0.00 | -0.09 | 0.00 | 0.05 | 0.07 | 0.09 | 0.01 |
| Poland | -0.62 | 0.02 | -0.33 | 0.01 | -0.39 | 0.01 | 0.69 | 0.04 |
| Prince Edward Islands | -0.26 | 0.08 | -0.12 | 0.04 | -0.16 | 0.10 | 0.18 | 0.07 |
| Puerto Rico | -0.22 | 0.00 | -0.09 | 0.00 | 0.02 | 0.03 | 0.12 | 0.01 |
| Qatar | -0.26 | 0.00 | -0.12 | 0.00 | 0.01 | 0.00 | 0.36 | 0.01 |
| Russia | -0.49 | 0.18 | -0.24 | 0.11 | -0.06 | 0.24 | 0.27 | 0.06 |
| Saint Kitts and Nevis | -0.21 | 0.00 | -0.09 | 0.00 | 0.03 | 0.00 | 0.12 | 0.00 |
| Saint Lucia | -0.21 | 0.00 | -0.09 | 0.00 | 0.07 | 0.02 | 0.11 | 0.00 |
| Saint Pierre and Miquelon | -0.56 | 0.06 | -0.29 | 0.02 | -0.39 | 0.06 | 0.46 | 0.03 |
| Saint Vincent and the Grenadines | -0.21 | 0.00 | -0.09 | 0.00 | 0.09 | 0.02 | 0.11 | 0.00 |
| Samoa | -0.18 | 0.00 | -0.08 | 0.00 | 0.02 | 0.01 | 0.08 | 0.00 |
| Sao Tome and Principe | -0.23 | 0.00 | -0.10 | 0.00 | -0.05 | 0.03 | 0.09 | 0.03 |
| Saudi Arabia | -0.25 | 0.01 | -0.11 | 0.01 | 0.08 | 0.21 | 0.22 | 0.07 |
| Senegal | -0.25 | 0.01 | -0.12 | 0.01 | -0.11 | 0.04 | 0.10 | 0.02 |
| Serbia and Montenegro | -0.37 | 0.01 | -0.19 | 0.01 | 0.00 | 0.00 | 0.14 | 0.00 |
| Seychelles | -0.22 | 0.00 | -0.10 | 0.00 | -0.04 | 0.03 | 0.07 | 0.01 |
| Sierra Leone | -0.23 | 0.00 | -0.10 | 0.00 | -0.05 | 0.01 | 0.07 | 0.00 |
| Singapore | -0.18 | 0.00 | -0.08 | 0.00 | 0.00 | 0.00 | 0.00 | 0.00 |
| Slovenia | -0.44 | 0.00 | -0.22 | 0.00 | 0.00 | 0.00 | 0.00 | 0.00 |
| Solomon Islands | -0.18 | 0.01 | -0.08 | 0.00 | -0.05 | 0.04 | 0.09 | 0.00 |
| Somalia | -0.23 | 0.01 | -0.10 | 0.00 | -0.01 | 0.05 | 0.07 | 0.01 |
| South Korea | -0.38 | 0.06 | -0.19 | 0.03 | -0.19 | 0.06 | 0.26 | 0.06 |
| Sri Lanka | -0.21 | 0.00 | -0.09 | 0.00 | 0.06 | 0.03 | 0.06 | 0.00 |
| Sudan | -0.24 | 0.00 | -0.10 | 0.00 | 0.20 | 0.08 | 0.20 | 0.01 |
| Suriname | -0.22 | 0.00 | -0.10 | 0.00 | 0.04 | 0.04 | 0.08 | 0.01 |
| Sweden | -0.66 | 0.05 | -0.35 | 0.03 | -0.33 | 0.06 | 0.72 | 0.04 |
| Syria | -0.32 | 0.00 | -0.16 | 0.00 | 0.00 | 0.00 | 0.14 | 0.00 |
| Taiwan | -0.20 | 0.00 | -0.09 | 0.00 | -0.01 | 0.07 | 0.10 | 0.01 |
| Tanzania | -0.21 | 0.00 | -0.09 | 0.00 | -0.15 | 0.06 | 0.07 | 0.00 |
| Thailand | -0.18 | 0.01 | -0.08 | 0.00 | 0.09 | 0.05 | 0.11 | 0.03 |
| Togo | -0.24 | 0.00 | -0.11 | 0.00 | -0.23 | 0.06 | 0.06 | 0.00 |
| Tonga | -0.20 | 0.01 | -0.09 | 0.01 | 0.02 | 0.02 | 0.09 | 0.00 |
| Trinidad and Tobago | -0.21 | 0.00 | -0.09 | 0.00 | 0.08 | 0.01 | 0.09 | 0.01 |
| Tunisia | -0.30 | 0.00 | -0.14 | 0.00 | 0.03 | 0.02 | 0.13 | 0.01 |
| Turkey | -0.33 | 0.02 | -0.17 | 0.01 | 0.01 | 0.02 | 0.28 | 0.07 |
| Tuvalu | -0.18 | 0.01 | -0.08 | 0.00 | -0.01 | 0.02 | 0.08 | 0.01 |
| United Arab Emirates | -0.25 | 0.00 | -0.11 | 0.00 | -0.05 | 0.00 | 0.29 | 0.05 |
| Uruguay | -0.34 | 0.03 | -0.18 | 0.03 | -0.24 | 0.08 | 0.23 | 0.08 |
| USA | -0.39 | 0.16 | -0.18 | 0.08 | -0.05 | 0.23 | 0.20 | 0.13 |
| Vanuatu | -0.19 | 0.01 | -0.08 | 0.00 | -0.03 | 0.01 | 0.08 | 0.00 |
| Venezuela | -0.22 | 0.01 | -0.10 | 0.01 | 0.13 | 0.08 | 0.11 | 0.01 |
| Vietnam | -0.19 | 0.01 | -0.08 | 0.00 | 0.08 | 0.04 | 0.11 | 0.02 |
| Yemen | -0.24 | 0.01 | -0.10 | 0.00 | 0.02 | 0.04 | 0.07 | 0.03 |

| pH | rcp85 | | rcp45 | | **Accuracy** | | **Precision** | |
| --- | --- | --- | --- | --- | --- | --- | --- | --- |
|  | MEAN | STD | MEAN | STD | MEAN | STD | MEAN | STD |
| Albania | -0.30 | 0.00 | -0.15 | 0.00 |  |  | 0.02 | 0.00 |
| Algeria | -0.31 | 0.00 | -0.15 | 0.00 |  |  | 0.02 | 0.00 |
| American Samoa | -0.30 | 0.01 | -0.15 | 0.00 | -0.03 | 0.02 | 0.01 | 0.00 |
| Angola | -0.28 | 0.01 | -0.14 | 0.00 | -0.04 | 0.01 | 0.03 | 0.02 |
| Anguilla | -0.30 | 0.01 | -0.15 | 0.01 | -0.02 | 0.01 | 0.01 | 0.00 |
| Antigua and Barbuda | -0.30 | 0.00 | -0.14 | 0.00 | -0.01 | 0.00 | 0.01 | 0.00 |
| Argentina | -0.33 | 0.01 | -0.16 | 0.00 | -0.11 | 0.02 | 0.01 | 0.00 |
| Australia | -0.32 | 0.01 | -0.15 | 0.01 | 0.02 | 0.04 | 0.01 | 0.00 |
| Bahamas | -0.29 | 0.00 | -0.14 | 0.00 | 0.00 | 0.01 | 0.01 | 0.00 |
| Bahrain | -0.27 | 0.00 | -0.14 | 0.00 |  |  | 0.10 | 0.00 |
| Bangladesh | -0.32 | 0.00 | -0.15 | 0.00 | -0.07 | 0.01 | 0.02 | 0.01 |
| Barbados | -0.30 | 0.00 | -0.14 | 0.00 | 0.01 | 0.00 | 0.01 | 0.00 |
| Belgium | -0.35 | 0.00 | -0.17 | 0.00 |  |  |  |  |
| Belize | -0.29 | 0.00 | -0.14 | 0.00 |  |  | 0.01 | 0.00 |
| Benin | -0.29 | 0.00 | -0.14 | 0.00 | 0.00 | 0.01 | 0.03 | 0.01 |
| Bosnia and Herzegovina | -0.31 | 0.00 | -0.15 | 0.00 |  |  |  |  |
| Bouvet Island | -0.37 | 0.02 | -0.19 | 0.02 | -0.06 | 0.00 | 0.03 | 0.02 |
| Brazil | -0.29 | 0.01 | -0.14 | 0.00 | -0.02 | 0.03 | 0.01 | 0.00 |
| Cambodia | -0.31 | 0.00 | -0.15 | 0.00 |  |  | 0.04 | 0.00 |
| Cameroon | -0.30 | 0.00 | -0.15 | 0.00 |  |  |  |  |
| Canada | -0.38 | 0.04 | -0.20 | 0.03 | -0.16 | 0.07 | 0.04 | 0.02 |
| Canary Islands | -0.31 | 0.01 | -0.16 | 0.01 | -0.03 | 0.02 | 0.02 | 0.00 |
| Cape Verde | -0.29 | 0.00 | -0.14 | 0.00 | 0.00 | 0.01 | 0.01 | 0.00 |
| Chile | -0.31 | 0.02 | -0.15 | 0.01 | -0.04 | 0.03 | 0.02 | 0.02 |
| China | -0.34 | 0.02 | -0.17 | 0.01 |  |  | 0.02 | 0.02 |
| Colombia | -0.28 | 0.01 | -0.14 | 0.00 | -0.01 | 0.02 | 0.03 | 0.02 |
| Comoros | -0.30 | 0.00 | -0.15 | 0.00 | 0.00 | 0.00 | 0.01 | 0.00 |
| Cook Islands | -0.32 | 0.01 | -0.16 | 0.01 | -0.03 | 0.04 | 0.01 | 0.00 |
| Costa Rica | -0.27 | 0.00 | -0.14 | 0.00 | -0.02 | 0.02 | 0.06 | 0.01 |
| Croatia | -0.31 | 0.00 | -0.16 | 0.00 |  |  | 0.04 | 0.02 |
| Cuba | -0.29 | 0.00 | -0.14 | 0.00 | 0.00 | 0.00 | 0.01 | 0.00 |
| Cyprus | -0.30 | 0.00 | -0.15 | 0.00 |  |  | 0.02 | 0.00 |
| Democratic Republic of the Congo | -0.31 | 0.00 | -0.15 | 0.00 | -0.06 | 0.00 | 0.07 | 0.00 |
| Denmark | -0.39 | 0.02 | -0.21 | 0.02 | -0.10 | 0.07 | 0.03 | 0.04 |
| Djibouti | -0.29 | 0.00 | -0.14 | 0.00 | 0.00 | 0.00 | 0.02 | 0.00 |
| Dominica | -0.30 | 0.00 | -0.14 | 0.00 | 0.01 | 0.01 | 0.01 | 0.00 |
| Dominican Republic | -0.29 | 0.00 | -0.14 | 0.00 | -0.01 | 0.00 | 0.01 | 0.00 |
| East Timor | -0.30 | 0.00 | -0.15 | 0.00 |  |  | 0.01 | 0.00 |
| Ecuador | -0.27 | 0.00 | -0.14 | 0.00 | -0.02 | 0.01 | 0.05 | 0.01 |
| Egypt | -0.29 | 0.01 | -0.14 | 0.00 |  |  | 0.03 | 0.00 |
| El Salvador | -0.27 | 0.00 | -0.14 | 0.00 | -0.01 | 0.00 | 0.06 | 0.00 |
| Equatorial Guinea | -0.29 | 0.01 | -0.14 | 0.00 | -0.07 | 0.01 | 0.04 | 0.01 |
| Eritrea | -0.28 | 0.00 | -0.14 | 0.00 |  |  | 0.04 | 0.00 |
| Estonia | -0.40 | 0.01 | -0.19 | 0.01 |  |  | 0.59 | 0.04 |
| Fiji | -0.31 | 0.01 | -0.15 | 0.00 | 0.00 | 0.02 | 0.01 | 0.00 |
| Finland | -0.41 | 0.01 | -0.20 | 0.01 |  |  | 0.63 | 0.03 |
| France | -0.33 | 0.01 | -0.17 | 0.00 | -0.06 | 0.01 | 0.02 | 0.01 |
| French Guiana | -0.29 | 0.00 | -0.14 | 0.00 | -0.01 | 0.01 | 0.01 | 0.00 |
| French Polynesia | -0.30 | 0.01 | -0.15 | 0.01 | -0.01 | 0.01 | 0.01 | 0.00 |
| Gabon | -0.29 | 0.00 | -0.15 | 0.00 | -0.06 | 0.01 | 0.06 | 0.01 |
| Gambia | -0.28 | 0.00 | -0.14 | 0.00 | 0.01 | 0.00 | 0.01 | 0.00 |
| Germany | -0.37 | 0.01 | -0.18 | 0.00 |  |  | 0.08 | 0.15 |
| Ghana | -0.28 | 0.00 | -0.14 | 0.00 | -0.03 | 0.01 | 0.01 | 0.00 |
| Gibraltar | -0.31 | 0.00 | -0.16 | 0.00 |  |  |  |  |
| Greece | -0.30 | 0.00 | -0.15 | 0.00 |  |  | 0.02 | 0.00 |
| Grenada | -0.29 | 0.00 | -0.14 | 0.00 | 0.02 | 0.00 | 0.01 | 0.00 |
| Guadeloupe | -0.30 | 0.00 | -0.14 | 0.00 | 0.00 | 0.01 | 0.01 | 0.00 |
| Guatemala | -0.27 | 0.00 | -0.14 | 0.00 | -0.01 | 0.01 | 0.06 | 0.00 |
| Guernsey | -0.35 | 0.01 | -0.17 | 0.01 | -0.04 | 0.01 | 0.03 | 0.01 |
| Guinea | -0.28 | 0.00 | -0.14 | 0.00 | 0.01 | 0.00 | 0.01 | 0.00 |
| Guinea-Bissau | -0.28 | 0.00 | -0.14 | 0.00 | 0.01 | 0.00 | 0.01 | 0.00 |
| Guyana | -0.29 | 0.00 | -0.14 | 0.00 | 0.02 | 0.00 | 0.01 | 0.00 |
| Haiti | -0.29 | 0.00 | -0.14 | 0.00 |  |  | 0.01 | 0.00 |
| Honduras | -0.29 | 0.00 | -0.14 | 0.00 |  |  | 0.01 | 0.00 |
| Iceland | -0.36 | 0.02 | -0.19 | 0.01 | -0.07 | 0.04 | 0.02 | 0.00 |
| India | -0.29 | 0.01 | -0.14 | 0.00 | -0.03 | 0.02 | 0.02 | 0.01 |
| Indonesia | -0.30 | 0.01 | -0.15 | 0.00 | -0.02 | 0.01 | 0.02 | 0.01 |
| Iran | -0.27 | 0.01 | -0.14 | 0.00 | -0.02 | 0.00 | 0.08 | 0.04 |
| Iraq | -0.26 | 0.00 | -0.13 | 0.00 |  |  | 0.13 | 0.00 |
| Ireland | -0.35 | 0.00 | -0.18 | 0.00 | -0.03 | 0.01 | 0.02 | 0.00 |
| Israel | -0.30 | 0.00 | -0.15 | 0.00 |  |  | 0.03 | 0.00 |
| Italy | -0.31 | 0.00 | -0.15 | 0.00 |  |  | 0.02 | 0.01 |
| Ivory Coast | -0.28 | 0.00 | -0.14 | 0.00 | -0.02 | 0.01 | 0.01 | 0.00 |
| Jamaica | -0.29 | 0.00 | -0.14 | 0.00 |  |  | 0.01 | 0.00 |
| Japan | -0.32 | 0.01 | -0.16 | 0.01 | -0.03 | 0.02 | 0.01 | 0.01 |
| Jordan | -0.29 | 0.00 | -0.14 | 0.00 |  |  |  |  |
| Kenya | -0.30 | 0.00 | -0.15 | 0.00 | -0.01 | 0.00 | 0.01 | 0.00 |
| Kiribati | -0.28 | 0.00 | -0.14 | 0.00 | -0.02 | 0.03 | 0.01 | 0.01 |
| Kuwait | -0.26 | 0.00 | -0.13 | 0.00 |  |  | 0.13 | 0.00 |
| Latvia | -0.39 | 0.00 | -0.19 | 0.00 |  |  | 0.60 | 0.04 |
| Lebanon | -0.30 | 0.00 | -0.15 | 0.00 |  |  | 0.03 | 0.00 |
| Liberia | -0.28 | 0.00 | -0.14 | 0.00 | -0.02 | 0.02 | 0.01 | 0.00 |
| Libya | -0.29 | 0.00 | -0.14 | 0.00 |  |  | 0.02 | 0.00 |
| Lithuania | -0.38 | 0.00 | -0.18 | 0.00 |  |  | 0.61 | 0.00 |
| Madagascar | -0.31 | 0.00 | -0.15 | 0.00 | 0.00 | 0.01 | 0.01 | 0.00 |
| Malaysia | -0.31 | 0.01 | -0.15 | 0.00 | -0.07 | 0.00 | 0.02 | 0.01 |
| Maldives | -0.29 | 0.00 | -0.14 | 0.00 | -0.01 | 0.01 | 0.01 | 0.00 |
| Malta | -0.30 | 0.00 | -0.15 | 0.00 |  |  | 0.02 | 0.00 |
| Marshall Islands | -0.30 | 0.01 | -0.14 | 0.00 | -0.02 | 0.01 | 0.01 | 0.00 |
| Mauritania | -0.28 | 0.00 | -0.14 | 0.00 | 0.00 | 0.01 | 0.01 | 0.00 |
| Mauritius | -0.31 | 0.01 | -0.15 | 0.00 | -0.01 | 0.01 | 0.01 | 0.00 |
| Mexico | -0.30 | 0.01 | -0.15 | 0.00 | 0.02 | 0.03 | 0.01 | 0.01 |
| Micronesia | -0.29 | 0.01 | -0.14 | 0.00 | -0.02 | 0.01 | 0.01 | 0.00 |
| Monaco | -0.31 | 0.00 | -0.16 | 0.00 |  |  | 0.01 | 0.00 |
| Morocco | -0.31 | 0.01 | -0.15 | 0.00 | -0.03 | 0.01 | 0.02 | 0.00 |
| Mozambique | -0.31 | 0.00 | -0.15 | 0.00 | 0.02 | 0.01 | 0.01 | 0.00 |
| Myanmar | -0.30 | 0.00 | -0.15 | 0.00 | -0.04 | 0.02 | 0.02 | 0.01 |
| Namibia | -0.28 | 0.01 | -0.14 | 0.01 | -0.06 | 0.02 | 0.02 | 0.01 |
| Nauru | -0.28 | 0.00 | -0.14 | 0.00 | -0.04 | 0.00 | 0.01 | 0.00 |
| Netherlands | -0.35 | 0.00 | -0.17 | 0.00 |  |  | 0.03 | 0.00 |
| Netherlands Antilles | -0.29 | 0.00 | -0.14 | 0.00 |  |  | 0.01 | 0.00 |
| New Caledonia | -0.32 | 0.00 | -0.16 | 0.00 | 0.01 | 0.02 | 0.01 | 0.00 |
| Nicaragua | -0.28 | 0.01 | -0.14 | 0.00 | -0.01 | 0.03 | 0.03 | 0.03 |
| Nigeria | -0.29 | 0.00 | -0.14 | 0.00 | -0.02 | 0.02 | 0.03 | 0.02 |
| North Korea | -0.35 | 0.00 | -0.18 | 0.00 |  |  | 0.03 | 0.00 |
| Oman | -0.29 | 0.00 | -0.14 | 0.00 | 0.06 | 0.03 | 0.02 | 0.01 |
| Pakistan | -0.29 | 0.00 | -0.15 | 0.00 | -0.01 | 0.01 | 0.02 | 0.00 |
| Palau | -0.30 | 0.00 | -0.14 | 0.00 | -0.03 | 0.01 | 0.01 | 0.00 |
| Panama | -0.27 | 0.01 | -0.14 | 0.00 | -0.02 | 0.03 | 0.04 | 0.02 |
| Papua New Guinea | -0.29 | 0.01 | -0.14 | 0.00 | 0.02 | 0.05 | 0.01 | 0.00 |
| Peru | -0.26 | 0.01 | -0.13 | 0.01 | -0.05 | 0.02 | 0.06 | 0.02 |
| Philippines | -0.31 | 0.00 | -0.15 | 0.00 | 0.02 | 0.02 | 0.01 | 0.00 |
| Poland | -0.39 | 0.00 | -0.18 | 0.00 |  |  | 0.61 | 0.01 |
| Prince Edward Islands | -0.32 | 0.00 | -0.16 | 0.00 | -0.05 | 0.05 | 0.01 | 0.00 |
| Puerto Rico | -0.29 | 0.00 | -0.14 | 0.00 | -0.01 | 0.00 | 0.01 | 0.00 |
| Qatar | -0.27 | 0.00 | -0.14 | 0.00 |  |  | 0.10 | 0.00 |
| Russia | -0.36 | 0.03 | -0.18 | 0.02 | -0.21 | 0.06 | 0.04 | 0.06 |
| Saint Kitts and Nevis | -0.30 | 0.00 | -0.14 | 0.00 |  |  | 0.01 | 0.00 |
| Saint Lucia | -0.29 | 0.00 | -0.14 | 0.00 | 0.02 | 0.00 | 0.01 | 0.00 |
| Saint Pierre and Miquelon | -0.34 | 0.01 | -0.17 | 0.01 | -0.14 | 0.03 | 0.02 | 0.00 |
| Saint Vincent and the Grenadines | -0.29 | 0.00 | -0.14 | 0.00 | 0.02 | 0.00 | 0.01 | 0.00 |
| Samoa | -0.31 | 0.00 | -0.15 | 0.00 | 0.00 | 0.00 | 0.02 | 0.00 |
| Sao Tome and Principe | -0.29 | 0.00 | -0.14 | 0.00 | -0.05 | 0.00 | 0.03 | 0.01 |
| Saudi Arabia | -0.28 | 0.00 | -0.14 | 0.00 |  |  | 0.04 | 0.03 |
| Senegal | -0.28 | 0.00 | -0.14 | 0.00 | 0.01 | 0.00 | 0.01 | 0.00 |
| Serbia and Montenegro | -0.31 | 0.00 | -0.15 | 0.00 |  |  | 0.02 | 0.00 |
| Seychelles | -0.30 | 0.00 | -0.14 | 0.00 | 0.01 | 0.00 | 0.01 | 0.00 |
| Sierra Leone | -0.28 | 0.00 | -0.14 | 0.00 | 0.01 | 0.01 | 0.01 | 0.00 |
| Singapore | -0.31 | 0.00 | -0.15 | 0.00 |  |  |  |  |
| Slovenia | -0.32 | 0.00 | -0.16 | 0.00 |  |  |  |  |
| Solomon Islands | -0.30 | 0.01 | -0.14 | 0.00 | 0.04 | 0.03 | 0.01 | 0.00 |
| Somalia | -0.30 | 0.00 | -0.15 | 0.00 | 0.00 | 0.02 | 0.01 | 0.00 |
| South Korea | -0.35 | 0.01 | -0.17 | 0.01 |  |  | 0.02 | 0.01 |
| Sri Lanka | -0.29 | 0.00 | -0.14 | 0.00 | -0.01 | 0.01 | 0.01 | 0.00 |
| Sudan | -0.28 | 0.00 | -0.14 | 0.00 |  |  | 0.03 | 0.00 |
| Suriname | -0.29 | 0.00 | -0.14 | 0.00 | 0.00 | 0.01 | 0.01 | 0.00 |
| Sweden | -0.40 | 0.01 | -0.19 | 0.01 |  |  | 0.61 | 0.08 |
| Syria | -0.29 | 0.00 | -0.15 | 0.00 |  |  | 0.03 | 0.00 |
| Taiwan | -0.31 | 0.00 | -0.15 | 0.00 | 0.03 | 0.00 | 0.01 | 0.00 |
| Tanzania | -0.30 | 0.00 | -0.15 | 0.00 | 0.00 | 0.00 | 0.01 | 0.00 |
| Thailand | -0.30 | 0.01 | -0.15 | 0.00 | -0.06 | 0.01 | 0.04 | 0.01 |
| Togo | -0.29 | 0.00 | -0.14 | 0.00 | -0.02 | 0.01 | 0.02 | 0.01 |
| Tonga | -0.32 | 0.00 | -0.15 | 0.00 | -0.03 | 0.02 | 0.01 | 0.00 |
| Trinidad and Tobago | -0.29 | 0.00 | -0.14 | 0.00 | 0.01 | 0.00 | 0.01 | 0.00 |
| Tunisia | -0.30 | 0.00 | -0.15 | 0.00 |  |  | 0.02 | 0.00 |
| Turkey | -0.30 | 0.00 | -0.15 | 0.00 |  |  | 0.18 | 0.06 |
| Tuvalu | -0.29 | 0.01 | -0.14 | 0.00 | -0.01 | 0.02 | 0.01 | 0.00 |
| United Arab Emirates | -0.27 | 0.00 | -0.14 | 0.00 |  |  | 0.08 | 0.01 |
| Uruguay | -0.32 | 0.01 | -0.15 | 0.00 | -0.10 | 0.03 | 0.01 | 0.00 |
| USA | -0.33 | 0.02 | -0.16 | 0.02 | -0.07 | 0.07 | 0.01 | 0.01 |
| Vanuatu | -0.31 | 0.00 | -0.15 | 0.00 | 0.02 | 0.02 | 0.01 | 0.00 |
| Venezuela | -0.29 | 0.00 | -0.14 | 0.00 | 0.02 | 0.00 | 0.01 | 0.00 |
| Vietnam | -0.31 | 0.00 | -0.15 | 0.00 |  |  | 0.01 | 0.01 |
| Yemen | -0.29 | 0.00 | -0.14 | 0.00 | 0.05 | 0.01 | 0.01 | 0.01 |

| **Phytoplankton Carbon Concentration** | **rcp85** | | **rcp45** | | **Accuracy** | | **Precision** | |
| --- | --- | --- | --- | --- | --- | --- | --- | --- |
| **(mg C/L)** | **MEAN** | **STD** | **MEAN** | **STD** | **MEAN** | **STD** | **MEAN** | **STD** |
| Albania | 0.0002 | 0.0002 | 0.0012 | 0.0002 | 0.0101 | 0.0000 | 0.0070 | 0.0003 |
| Algeria | -0.0016 | 0.0011 | -0.0009 | 0.0006 | 0.0181 | 0.0026 | 0.0131 | 0.0018 |
| American Samoa | -0.0025 | 0.0038 | -0.0013 | 0.0018 | 0.0149 | 0.0128 | 0.0095 | 0.0105 |
| Angola | -0.0148 | 0.0076 | -0.0092 | 0.0049 | 0.0342 | 0.0108 | 0.0310 | 0.0137 |
| Anguilla | -0.0009 | 0.0008 | -0.0004 | 0.0006 | 0.0114 | 0.0034 | 0.0050 | 0.0008 |
| Antigua and Barbuda | -0.0002 | 0.0001 | -0.0001 | 0.0001 | 0.0073 | 0.0001 | 0.0047 | 0.0002 |
| Argentina | -0.0062 | 0.0065 | -0.0017 | 0.0036 | 0.0673 | 0.0172 | 0.0337 | 0.0127 |
| Australia | -0.0033 | 0.0029 | -0.0012 | 0.0011 | 0.0209 | 0.0122 | 0.0106 | 0.0068 |
| Bahamas | -0.0006 | 0.0007 | -0.0002 | 0.0006 | 0.0098 | 0.0013 | 0.0050 | 0.0004 |
| Bahrain | -0.0019 | 0.0001 | -0.0005 | 0.0000 | 0.0125 | 0.0000 | 0.0081 | 0.0001 |
| Bangladesh | -0.0018 | 0.0006 | -0.0012 | 0.0003 | 0.0200 | 0.0012 | 0.0090 | 0.0012 |
| Barbados | -0.0014 | 0.0008 | -0.0009 | 0.0005 | 0.0112 | 0.0022 | 0.0049 | 0.0007 |
| Belgium | -0.0091 | 0.0001 | -0.0060 | 0.0001 |  |  |  |  |
| Belize | -0.0025 | 0.0002 | -0.0016 | 0.0002 | 0.0132 | 0.0000 | 0.0052 | 0.0004 |
| Benin | -0.0078 | 0.0015 | -0.0047 | 0.0011 | 0.0253 | 0.0024 | 0.0200 | 0.0036 |
| Bosnia and Herzegovina | -0.0009 | 0.0000 | 0.0008 | 0.0000 |  |  |  |  |
| Bouvet Island | -0.0052 | 0.0040 | -0.0027 | 0.0030 | 0.0299 | 0.0047 | 0.0152 | 0.0063 |
| Brazil | -0.0038 | 0.0039 | -0.0020 | 0.0023 | 0.0178 | 0.0089 | 0.0119 | 0.0085 |
| Cambodia | -0.0015 | 0.0004 | -0.0007 | 0.0002 | 0.0173 | 0.0010 | 0.0171 | 0.0072 |
| Cameroon | -0.0039 | 0.0003 | -0.0021 | 0.0002 |  |  |  |  |
| Canada | -0.0039 | 0.0043 | -0.0022 | 0.0025 | 0.0297 | 0.0074 | 0.0145 | 0.0069 |
| Canary Islands | -0.0029 | 0.0034 | -0.0001 | 0.0045 | 0.0258 | 0.0186 | 0.0230 | 0.0230 |
| Cape Verde | -0.0053 | 0.0022 | -0.0026 | 0.0011 | 0.0227 | 0.0053 | 0.0131 | 0.0036 |
| Chile | 0.0006 | 0.0059 | 0.0004 | 0.0027 | 0.0444 | 0.0363 | 0.0347 | 0.0381 |
| China | -0.0033 | 0.0016 | -0.0018 | 0.0009 | 0.0194 | 0.0077 | 0.0150 | 0.0112 |
| Colombia | -0.0063 | 0.0020 | -0.0033 | 0.0017 | 0.0294 | 0.0122 | 0.0193 | 0.0101 |
| Comoros | -0.0013 | 0.0003 | -0.0006 | 0.0001 | 0.0115 | 0.0012 | 0.0059 | 0.0005 |
| Cook Islands | -0.0017 | 0.0032 | -0.0001 | 0.0022 | 0.0246 | 0.0188 | 0.0150 | 0.0155 |
| Costa Rica | -0.0066 | 0.0016 | -0.0030 | 0.0009 | 0.0431 | 0.0060 | 0.0311 | 0.0059 |
| Croatia | -0.0010 | 0.0009 | 0.0002 | 0.0006 | 0.0132 | 0.0012 | 0.0122 | 0.0040 |
| Cuba | -0.0016 | 0.0002 | -0.0010 | 0.0001 | 0.0114 | 0.0009 | 0.0046 | 0.0006 |
| Cyprus | 0.0000 | 0.0002 | 0.0003 | 0.0001 | 0.0079 | 0.0003 | 0.0059 | 0.0004 |
| Democratic Republic of the Congo | -0.0087 | 0.0019 | -0.0052 | 0.0013 | 0.0352 | 0.0000 | 0.0414 | 0.0045 |
| Denmark | -0.0059 | 0.0025 | -0.0029 | 0.0016 | 0.0331 | 0.0073 | 0.0174 | 0.0057 |
| Djibouti | -0.0019 | 0.0002 | -0.0004 | 0.0001 | 0.0182 | 0.0000 | 0.0106 | 0.0004 |
| Dominica | -0.0010 | 0.0007 | -0.0007 | 0.0004 | 0.0097 | 0.0015 | 0.0055 | 0.0007 |
| Dominican Republic | -0.0013 | 0.0008 | -0.0008 | 0.0006 | 0.0099 | 0.0018 | 0.0054 | 0.0008 |
| East Timor | -0.0030 | 0.0006 | -0.0016 | 0.0003 | 0.0157 | 0.0005 | 0.0082 | 0.0018 |
| Ecuador | -0.0168 | 0.0080 | -0.0064 | 0.0034 | 0.0753 | 0.0242 | 0.0616 | 0.0228 |
| Egypt | 0.0000 | 0.0002 | 0.0003 | 0.0002 | 0.0079 | 0.0009 | 0.0057 | 0.0008 |
| El Salvador | -0.0073 | 0.0019 | -0.0041 | 0.0005 | 0.0352 | 0.0042 | 0.0254 | 0.0048 |
| Equatorial Guinea | -0.0083 | 0.0023 | -0.0049 | 0.0016 | 0.0277 | 0.0032 | 0.0226 | 0.0060 |
| Eritrea | -0.0013 | 0.0004 | -0.0003 | 0.0001 | 0.0114 | 0.0004 | 0.0094 | 0.0023 |
| Estonia | -0.0025 | 0.0003 | -0.0011 | 0.0003 | 0.0245 | 0.0001 | 0.0324 | 0.0029 |
| Fiji | -0.0004 | 0.0003 | -0.0002 | 0.0002 | 0.0082 | 0.0009 | 0.0039 | 0.0002 |
| Finland | -0.0015 | 0.0015 | 0.0000 | 0.0013 | 0.0272 | 0.0021 | 0.0349 | 0.0032 |
| France | -0.0037 | 0.0024 | -0.0022 | 0.0020 | 0.0221 | 0.0038 | 0.0180 | 0.0060 |
| French Guiana | -0.0044 | 0.0010 | -0.0024 | 0.0005 | 0.0236 | 0.0032 | 0.0094 | 0.0010 |
| French Polynesia | -0.0002 | 0.0006 | 0.0000 | 0.0003 | 0.0091 | 0.0039 | 0.0052 | 0.0018 |
| Gabon | -0.0094 | 0.0020 | -0.0056 | 0.0013 | 0.0316 | 0.0033 | 0.0226 | 0.0060 |
| Gambia | -0.0141 | 0.0056 | -0.0082 | 0.0035 | 0.0401 | 0.0107 | 0.0304 | 0.0105 |
| Germany | -0.0078 | 0.0013 | -0.0054 | 0.0011 | 0.0268 | 0.0029 | 0.0113 | 0.0045 |
| Ghana | -0.0083 | 0.0028 | -0.0044 | 0.0017 | 0.0239 | 0.0038 | 0.0187 | 0.0059 |
| Gibraltar | -0.0031 | 0.0002 | -0.0020 | 0.0001 |  |  |  |  |
| Greece | -0.0002 | 0.0006 | 0.0005 | 0.0003 | 0.0086 | 0.0005 | 0.0069 | 0.0017 |
| Grenada | -0.0065 | 0.0017 | -0.0038 | 0.0014 | 0.0240 | 0.0049 | 0.0145 | 0.0056 |
| Guadeloupe | -0.0006 | 0.0006 | -0.0005 | 0.0004 | 0.0086 | 0.0013 | 0.0051 | 0.0007 |
| Guatemala | -0.0062 | 0.0009 | -0.0032 | 0.0004 | 0.0297 | 0.0029 | 0.0188 | 0.0028 |
| Guernsey | -0.0099 | 0.0034 | -0.0064 | 0.0021 | 0.0340 | 0.0072 | 0.0186 | 0.0066 |
| Guinea | -0.0074 | 0.0020 | -0.0050 | 0.0016 | 0.0240 | 0.0043 | 0.0190 | 0.0063 |
| Guinea-Bissau | -0.0092 | 0.0028 | -0.0056 | 0.0021 | 0.0279 | 0.0079 | 0.0213 | 0.0074 |
| Guyana | -0.0066 | 0.0018 | -0.0037 | 0.0008 | 0.0248 | 0.0066 | 0.0132 | 0.0045 |
| Haiti | -0.0015 | 0.0008 | -0.0010 | 0.0005 | 0.0107 | 0.0015 | 0.0051 | 0.0010 |
| Honduras | -0.0030 | 0.0005 | -0.0019 | 0.0003 | 0.0132 | 0.0010 | 0.0062 | 0.0009 |
| Iceland | -0.0067 | 0.0012 | -0.0030 | 0.0014 | 0.0345 | 0.0031 | 0.0171 | 0.0022 |
| India | -0.0028 | 0.0021 | -0.0014 | 0.0009 | 0.0199 | 0.0038 | 0.0113 | 0.0041 |
| Indonesia | -0.0017 | 0.0014 | -0.0010 | 0.0008 | 0.0164 | 0.0044 | 0.0075 | 0.0034 |
| Iran | -0.0031 | 0.0015 | -0.0008 | 0.0005 | 0.0239 | 0.0071 | 0.0124 | 0.0043 |
| Iraq | -0.0005 | 0.0001 | 0.0001 | 0.0000 |  |  | 0.0082 | 0.0000 |
| Ireland | -0.0101 | 0.0020 | -0.0076 | 0.0016 | 0.0310 | 0.0031 | 0.0242 | 0.0048 |
| Israel | 0.0003 | 0.0001 | 0.0004 | 0.0000 | 0.0070 | 0.0000 | 0.0052 | 0.0001 |
| Italy | 0.0000 | 0.0009 | 0.0002 | 0.0006 | 0.0131 | 0.0029 | 0.0096 | 0.0021 |
| Ivory Coast | -0.0090 | 0.0026 | -0.0047 | 0.0014 | 0.0257 | 0.0049 | 0.0203 | 0.0062 |
| Jamaica | -0.0025 | 0.0012 | -0.0016 | 0.0008 | 0.0121 | 0.0020 | 0.0060 | 0.0015 |
| Japan | -0.0018 | 0.0017 | -0.0008 | 0.0008 | 0.0111 | 0.0060 | 0.0052 | 0.0024 |
| Jordan | 0.0001 | 0.0000 | 0.0003 | 0.0000 |  |  |  |  |
| Kenya | -0.0028 | 0.0004 | -0.0015 | 0.0003 | 0.0149 | 0.0010 | 0.0070 | 0.0007 |
| Kiribati | -0.0048 | 0.0037 | -0.0023 | 0.0018 | 0.0251 | 0.0133 | 0.0168 | 0.0122 |
| Kuwait | -0.0007 | 0.0001 | 0.0000 | 0.0001 | 0.0115 | 0.0000 | 0.0083 | 0.0001 |
| Latvia | -0.0032 | 0.0008 | -0.0019 | 0.0008 | 0.0247 | 0.0005 | 0.0321 | 0.0042 |
| Lebanon | 0.0002 | 0.0000 | 0.0003 | 0.0000 | 0.0070 | 0.0000 | 0.0052 | 0.0001 |
| Liberia | -0.0070 | 0.0015 | -0.0038 | 0.0009 | 0.0220 | 0.0030 | 0.0155 | 0.0036 |
| Libya | 0.0003 | 0.0003 | 0.0004 | 0.0002 | 0.0081 | 0.0007 | 0.0064 | 0.0014 |
| Lithuania | -0.0037 | 0.0004 | -0.0025 | 0.0003 | 0.0225 | 0.0000 | 0.0315 | 0.0038 |
| Madagascar | -0.0010 | 0.0003 | -0.0005 | 0.0002 | 0.0091 | 0.0013 | 0.0048 | 0.0008 |
| Malaysia | -0.0014 | 0.0005 | -0.0009 | 0.0004 | 0.0149 | 0.0025 | 0.0076 | 0.0030 |
| Maldives | -0.0029 | 0.0008 | -0.0012 | 0.0004 | 0.0165 | 0.0010 | 0.0082 | 0.0008 |
| Malta | 0.0004 | 0.0003 | 0.0006 | 0.0002 | 0.0093 | 0.0010 | 0.0077 | 0.0008 |
| Marshall Islands | -0.0021 | 0.0007 | -0.0011 | 0.0006 | 0.0121 | 0.0033 | 0.0054 | 0.0020 |
| Mauritania | -0.0275 | 0.0091 | -0.0141 | 0.0044 | 0.0709 | 0.0279 | 0.0718 | 0.0360 |
| Mauritius | -0.0011 | 0.0005 | -0.0004 | 0.0002 | 0.0099 | 0.0021 | 0.0052 | 0.0005 |
| Mexico | -0.0039 | 0.0017 | -0.0021 | 0.0009 | 0.0168 | 0.0044 | 0.0106 | 0.0055 |
| Micronesia | -0.0022 | 0.0010 | -0.0013 | 0.0006 | 0.0125 | 0.0037 | 0.0062 | 0.0025 |
| Monaco | -0.0007 | 0.0000 | -0.0005 | 0.0000 | 0.0146 | 0.0000 | 0.0102 | 0.0000 |
| Morocco | -0.0021 | 0.0024 | 0.0033 | 0.0062 | 0.0392 | 0.0177 | 0.0332 | 0.0232 |
| Mozambique | -0.0011 | 0.0003 | -0.0006 | 0.0002 | 0.0104 | 0.0008 | 0.0052 | 0.0004 |
| Myanmar | -0.0007 | 0.0005 | -0.0006 | 0.0004 | 0.0202 | 0.0032 | 0.0108 | 0.0023 |
| Namibia | -0.0399 | 0.0217 | -0.0213 | 0.0125 | 0.0987 | 0.0424 | 0.1067 | 0.0602 |
| Nauru | -0.0063 | 0.0021 | -0.0031 | 0.0008 | 0.0244 | 0.0056 | 0.0168 | 0.0063 |
| Netherlands | -0.0095 | 0.0007 | -0.0065 | 0.0005 | 0.0340 | 0.0024 | 0.0137 | 0.0029 |
| Netherlands Antilles | -0.0073 | 0.0043 | -0.0048 | 0.0028 | 0.0180 | 0.0039 | 0.0131 | 0.0058 |
| New Caledonia | -0.0007 | 0.0003 | -0.0002 | 0.0001 | 0.0083 | 0.0005 | 0.0042 | 0.0005 |
| Nicaragua | -0.0052 | 0.0022 | -0.0032 | 0.0010 | 0.0274 | 0.0124 | 0.0162 | 0.0117 |
| Nigeria | -0.0058 | 0.0010 | -0.0033 | 0.0007 | 0.0218 | 0.0021 | 0.0138 | 0.0024 |
| North Korea | -0.0040 | 0.0011 | -0.0019 | 0.0007 | 0.0219 | 0.0014 | 0.0109 | 0.0067 |
| Oman | -0.0100 | 0.0045 | -0.0041 | 0.0019 | 0.0365 | 0.0081 | 0.0240 | 0.0149 |
| Pakistan | -0.0037 | 0.0016 | -0.0009 | 0.0010 | 0.0278 | 0.0025 | 0.0130 | 0.0025 |
| Palau | -0.0023 | 0.0006 | -0.0013 | 0.0003 | 0.0111 | 0.0014 | 0.0055 | 0.0006 |
| Panama | -0.0044 | 0.0012 | -0.0022 | 0.0008 | 0.0333 | 0.0144 | 0.0212 | 0.0109 |
| Papua New Guinea | -0.0026 | 0.0010 | -0.0015 | 0.0007 | 0.0146 | 0.0029 | 0.0070 | 0.0026 |
| Peru | -0.0257 | 0.0086 | -0.0091 | 0.0045 | 0.1282 | 0.0394 | 0.1443 | 0.0582 |
| Philippines | -0.0011 | 0.0005 | -0.0006 | 0.0003 | 0.0096 | 0.0015 | 0.0057 | 0.0013 |
| Poland | -0.0047 | 0.0003 | -0.0029 | 0.0002 | 0.0234 | 0.0014 | 0.0246 | 0.0018 |
| Prince Edward Islands | 0.0008 | 0.0059 | 0.0000 | 0.0025 | 0.0293 | 0.0168 | 0.0153 | 0.0197 |
| Puerto Rico | -0.0009 | 0.0006 | -0.0006 | 0.0004 | 0.0094 | 0.0017 | 0.0053 | 0.0006 |
| Qatar | -0.0026 | 0.0007 | -0.0007 | 0.0003 | 0.0151 | 0.0003 | 0.0081 | 0.0002 |
| Russia | -0.0005 | 0.0041 | 0.0002 | 0.0033 | 0.0387 | 0.0200 | 0.0192 | 0.0117 |
| Saint Kitts and Nevis | -0.0005 | 0.0002 | -0.0003 | 0.0001 | 0.0095 | 0.0000 | 0.0050 | 0.0003 |
| Saint Lucia | -0.0023 | 0.0006 | -0.0015 | 0.0003 | 0.0140 | 0.0019 | 0.0069 | 0.0006 |
| Saint Pierre and Miquelon | -0.0074 | 0.0009 | -0.0048 | 0.0005 | 0.0245 | 0.0007 | 0.0142 | 0.0006 |
| Saint Vincent and the Grenadines | -0.0037 | 0.0008 | -0.0022 | 0.0005 | 0.0163 | 0.0016 | 0.0087 | 0.0014 |
| Samoa | -0.0004 | 0.0001 | -0.0002 | 0.0001 | 0.0090 | 0.0003 | 0.0039 | 0.0001 |
| Sao Tome and Principe | -0.0052 | 0.0008 | -0.0027 | 0.0005 | 0.0215 | 0.0025 | 0.0129 | 0.0025 |
| Saudi Arabia | -0.0007 | 0.0006 | -0.0001 | 0.0002 | 0.0102 | 0.0016 | 0.0061 | 0.0012 |
| Senegal | -0.0151 | 0.0081 | -0.0085 | 0.0047 | 0.0368 | 0.0109 | 0.0317 | 0.0173 |
| Serbia and Montenegro | -0.0002 | 0.0008 | 0.0012 | 0.0004 | 0.0101 | 0.0000 | 0.0072 | 0.0000 |
| Seychelles | -0.0021 | 0.0002 | -0.0010 | 0.0002 | 0.0137 | 0.0010 | 0.0063 | 0.0008 |
| Sierra Leone | -0.0057 | 0.0016 | -0.0035 | 0.0011 | 0.0201 | 0.0032 | 0.0133 | 0.0032 |
| Singapore | -0.0010 | 0.0001 | -0.0004 | 0.0001 |  |  |  |  |
| Slovenia | -0.0031 | 0.0000 | -0.0008 | 0.0000 |  |  |  |  |
| Solomon Islands | -0.0017 | 0.0004 | -0.0010 | 0.0003 | 0.0110 | 0.0012 | 0.0045 | 0.0007 |
| Somalia | -0.0044 | 0.0012 | -0.0020 | 0.0005 | 0.0216 | 0.0037 | 0.0120 | 0.0032 |
| South Korea | -0.0038 | 0.0009 | -0.0020 | 0.0005 | 0.0223 | 0.0034 | 0.0145 | 0.0063 |
| Sri Lanka | -0.0057 | 0.0010 | -0.0027 | 0.0007 | 0.0233 | 0.0026 | 0.0129 | 0.0031 |
| Sudan | -0.0006 | 0.0001 | -0.0001 | 0.0001 | 0.0098 | 0.0006 | 0.0057 | 0.0003 |
| Suriname | -0.0069 | 0.0019 | -0.0037 | 0.0008 | 0.0273 | 0.0068 | 0.0152 | 0.0056 |
| Sweden | -0.0034 | 0.0025 | -0.0017 | 0.0022 | 0.0253 | 0.0022 | 0.0296 | 0.0055 |
| Syria | 0.0004 | 0.0001 | 0.0003 | 0.0000 |  |  | 0.0065 | 0.0001 |
| Taiwan | -0.0014 | 0.0005 | -0.0008 | 0.0003 | 0.0105 | 0.0012 | 0.0050 | 0.0005 |
| Tanzania | -0.0020 | 0.0003 | -0.0010 | 0.0002 | 0.0130 | 0.0007 | 0.0058 | 0.0005 |
| Thailand | -0.0012 | 0.0005 | -0.0008 | 0.0006 | 0.0177 | 0.0026 | 0.0077 | 0.0025 |
| Togo | -0.0083 | 0.0021 | -0.0048 | 0.0014 | 0.0240 | 0.0024 | 0.0218 | 0.0056 |
| Tonga | -0.0001 | 0.0002 | -0.0001 | 0.0001 | 0.0074 | 0.0006 | 0.0039 | 0.0001 |
| Trinidad and Tobago | -0.0054 | 0.0023 | -0.0034 | 0.0015 | 0.0176 | 0.0016 | 0.0100 | 0.0042 |
| Tunisia | -0.0002 | 0.0002 | 0.0000 | 0.0003 | 0.0111 | 0.0019 | 0.0105 | 0.0008 |
| Turkey | -0.0001 | 0.0005 | 0.0003 | 0.0003 | 0.0083 | 0.0001 | 0.0222 | 0.0070 |
| Tuvalu | -0.0008 | 0.0005 | -0.0005 | 0.0003 | 0.0116 | 0.0019 | 0.0053 | 0.0011 |
| United Arab Emirates | -0.0048 | 0.0009 | -0.0016 | 0.0004 | 0.0190 | 0.0000 | 0.0112 | 0.0023 |
| Uruguay | -0.0087 | 0.0021 | -0.0046 | 0.0010 | 0.0284 | 0.0052 | 0.0144 | 0.0065 |
| USA | -0.0023 | 0.0044 | -0.0013 | 0.0026 | 0.0278 | 0.0180 | 0.0172 | 0.0151 |
| Vanuatu | -0.0007 | 0.0004 | -0.0004 | 0.0001 | 0.0085 | 0.0007 | 0.0036 | 0.0002 |
| Venezuela | -0.0086 | 0.0052 | -0.0053 | 0.0030 | 0.0219 | 0.0127 | 0.0204 | 0.0188 |
| Vietnam | -0.0014 | 0.0003 | -0.0005 | 0.0002 | 0.0127 | 0.0017 | 0.0063 | 0.0036 |
| Yemen | -0.0066 | 0.0042 | -0.0027 | 0.0019 | 0.0281 | 0.0044 | 0.0172 | 0.0044 |

**References**

1. Halpern BS, Longo C, Hardy D, McLeod KL, Samhouri JF, et al. (2012) An index to assess the health and benefits of the global ocean. Nature 488: 615-620.
