# Supplementary material for: Biotic and Human Vulnerability to Projected Changes in Ocean Biogeochemistry over the 21st Century
Source: PLoS Biol. 2013 Oct 15;11(10):e1001682. doi: 10.1371/journal.pbio.1001682 (PMC3797030; doi:10.1371/journal.pbio.1001682)
Supplement: Table S6 — Source of data. (DOCX) [file pbio.1001682.s008.docx]

# Table S6. Source of data.

| Variable | Source | URL |
| --- | --- | --- |
| Climate Change Data |  |  |
| All parameters analyzed here (Table S1) | CMIP5 | <http://esgf.org/wiki/Cmip5Status/P2PArchiveView> |
| Data for the test of accuracy |  |  |
| Temperature | WorldOcean database | <http://www.nodc.noaa.gov/OC5/WOA09/netcdf_data.html> |
| Oxygen | WorldOcean database | <http://www.nodc.noaa.gov/OC5/WOA09/netcdf_data.html> |
| Carbon Flux | Lutz et al [[1](#_ENREF_1)] |  |
| Primary carbon concentration | Ocean Color | <http://oceancolor.gsfc.nasa.gov/cgi/l3> |
| pH (calculated with the software CO2Sys (ver 16) using the following variables | | |
| Alkalinity | Global Ocean Data Analysis Project | <http://cdiac3.ornl.gov/las/servlets/dataset?lastvar=1> |
| Total carbon | Global Ocean Data Analysis Project | <http://cdiac3.ornl.gov/las/servlets/dataset?lastvar=1> |
| Salinity | WorldOcean database | http://www.nodc.noaa.gov/OC5/WOA09/netcdf_data.html |
| Phospate | WorldOcean database | http://www.nodc.noaa.gov/OC5/WOA09/netcdf_data.html |
| Silicate | WorldOcean database | http://www.nodc.noaa.gov/OC5/WOA09/netcdf_data.html |
| Depth | WorldOcean database | <http://www.nodc.noaa.gov/OC5/WOA09/netcdf_data.html> |
| Software | CO2Sys (ver 16) | http://www.ecy.wa.gov/programs/eap/models/co2sys_ver16.zip, http://cdiac.esd.ornl.gov/oceans/co2rprt.html#phscales |
|  |  |  |
|  |  |  |
| Marine Habitats |  |  |
| Canyons | Harris & Whiteway | <http://oceanids.geoiq.grida.no/overlays/6574> |
| Coral reefs | UNEP-WCMC | [http://data.unep-wcmc.org/datasets](http://www.nceas.ucsb.edu/globalmarine/ecosystems) |
| Deep water | Halpern et al [[2](#_ENREF_2)] | <http://www.nceas.ucsb.edu/globalmarine/ecosystems> |
| Hard deep | Halpern et al [[2](#_ENREF_2)] | <http://www.nceas.ucsb.edu/globalmarine/ecosystems> |
| Hard shelf | Halpern et al [[2](#_ENREF_2)] | <http://www.nceas.ucsb.edu/globalmarine/ecosystems> |
| Hard slope | Halpern et al [[2](#_ENREF_2)] | <http://www.nceas.ucsb.edu/globalmarine/ecosystems> |
| Mangroves | United States Geological Survey | [http://data.unep-wcmc.org/datasets](http://www.nceas.ucsb.edu/globalmarine/ecosystems) |
| Rocky reefs | Halpern et al [[2](#_ENREF_2)] | <http://www.nceas.ucsb.edu/globalmarine/ecosystems> |
| Seagrasses | UNEP-WCMC | [http://data.unep-wcmc.org/datasets](http://www.nceas.ucsb.edu/globalmarine/ecosystems) |
| Seamounts | Halpern et al [[2](#_ENREF_2)] | <http://www.nceas.ucsb.edu/globalmarine/ecosystems> |
| Shoft shallow | Halpern et al [[2](#_ENREF_2)] | <http://www.nceas.ucsb.edu/globalmarine/ecosystems> |
| Soft deep | Halpern et al [[2](#_ENREF_2)] | <http://www.nceas.ucsb.edu/globalmarine/ecosystems> |
| Soft shelf | Halpern et al [[2](#_ENREF_2)] | <http://www.nceas.ucsb.edu/globalmarine/ecosystems> |
| Soft slope | Halpern et al [[2](#_ENREF_2)] | <http://www.nceas.ucsb.edu/globalmarine/ecosystems> |
| Surface water | Halpern et al [[2](#_ENREF_2)] | <http://www.nceas.ucsb.edu/globalmarine/ecosystems> |
| Vents | Halpern et al [[2](#_ENREF_2)] | <http://www.nceas.ucsb.edu/globalmarine/ecosystems> |
| Biodiversity Hotspot |  |  |
| All coastal species | Tittensor et al [[3](#_ENREF_3)] | <http://www.mathstat.dal.ca/~derekt/publications/global_patterns_and_predictors_of_marine_biodiversity_across_taxa.zip> |
| All taxa | Tittensor et al [[3](#_ENREF_3)] | <http://www.mathstat.dal.ca/~derekt/publications/global_patterns_and_predictors_of_marine_biodiversity_across_taxa.zip> |
| Cetaceans | Tittensor et al [[3](#_ENREF_3)] | <http://www.mathstat.dal.ca/~derekt/publications/global_patterns_and_predictors_of_marine_biodiversity_across_taxa.zip> |
| Coastal fishes | Tittensor et al [[3](#_ENREF_3)] | <http://www.mathstat.dal.ca/~derekt/publications/global_patterns_and_predictors_of_marine_biodiversity_across_taxa.zip> |
| Coral reefs | Tittensor et al [[3](#_ENREF_3)] | <http://www.mathstat.dal.ca/~derekt/publications/global_patterns_and_predictors_of_marine_biodiversity_across_taxa.zip> |
| Euphausiids | Tittensor et al [[3](#_ENREF_3)] | <http://www.mathstat.dal.ca/~derekt/publications/global_patterns_and_predictors_of_marine_biodiversity_across_taxa.zip> |
| Forams | Tittensor et al [[3](#_ENREF_3)] | <http://www.mathstat.dal.ca/~derekt/publications/global_patterns_and_predictors_of_marine_biodiversity_across_taxa.zip> |
| Mangroves | Tittensor et al [[3](#_ENREF_3)] | <http://www.mathstat.dal.ca/~derekt/publications/global_patterns_and_predictors_of_marine_biodiversity_across_taxa.zip> |
| Non-oceanic sharks | Tittensor et al [[3](#_ENREF_3)] | <http://www.mathstat.dal.ca/~derekt/publications/global_patterns_and_predictors_of_marine_biodiversity_across_taxa.zip> |
| Non-squid cephalopod | Tittensor et al [[3](#_ENREF_3)] | <http://www.mathstat.dal.ca/~derekt/publications/global_patterns_and_predictors_of_marine_biodiversity_across_taxa.zip> |
| Oceanic sharks | Tittensor et al [[3](#_ENREF_3)] | <http://www.mathstat.dal.ca/~derekt/publications/global_patterns_and_predictors_of_marine_biodiversity_across_taxa.zip> |
| Oceanic species | Tittensor et al [[3](#_ENREF_3)] | <http://www.mathstat.dal.ca/~derekt/publications/global_patterns_and_predictors_of_marine_biodiversity_across_taxa.zip> |
| Pinnipeds | Tittensor et al [[3](#_ENREF_3)] | <http://www.mathstat.dal.ca/~derekt/publications/global_patterns_and_predictors_of_marine_biodiversity_across_taxa.zip> |
| Seagrasses | Tittensor et al [[3](#_ENREF_3)] | <http://www.mathstat.dal.ca/~derekt/publications/global_patterns_and_predictors_of_marine_biodiversity_across_taxa.zip> |
| Squids | Tittensor et al [[3](#_ENREF_3)] | <http://www.mathstat.dal.ca/~derekt/publications/global_patterns_and_predictors_of_marine_biodiversity_across_taxa.zip> |
| Tunas and Bill fishes | Tittensor et al [[3](#_ENREF_3)] | <http://www.mathstat.dal.ca/~derekt/publications/global_patterns_and_predictors_of_marine_biodiversity_across_taxa.zip> |
|  |  |  |
| Socio-economic data |  |  |
| Gridded human population  (counts of people in 2000) | Center for International Earth Science Information Network (CIESIN) | http://sedac.ciesin.columbia.edu/data/set/gpw-v3-population-count/data-download |
| Work Force | World Bank | <http://databank.worldbank.org/databank/download/WDIandGDF_excel.zip> |
| Gross Domestic Product | World Bank | <http://databank.worldbank.org/databank/download/WDIandGDF_excel.zip> |
| Animal Food Supply | FAO | <http://faostat3.fao.org/home/index.html#DOWNLOAD> |
| Ocean Jobs, revenues and food | Halper et al [[4](#_ENREF_4)] | <ftp://ohi.nceas.ucsb.edu/pub/data/2012/index.html> |
|  |  |  |
| Data for RCPs in Fig. S1 | RCP Concentrations Calculation and Data | <http://www.pik-potsdam.de/~mmalte/rcps/> |

References

1. Lutz MJ, Caldeira K, Dunbar RB, Behrenfeld MJ (2007) Seasonal rhythms of net primary production and particulate organic carbon flux to depth describe the efficiency of biological pump in the global ocean. J Geophys Res 112: C10011.

2. Halpern BS, Walbridge S, Selkoe KA, Kappel CV, Micheli F, et al. (2008) A global map of human impact on marine ecosystems. Science 319: 948-952.

3. Tittensor DP, Mora C, Jetz W, Lotze HK, Ricard D, et al. (2010) Global patterns and predictors of marine biodiversity across taxa. Nature 466: 1098-1101.

4. Halpern BS, Longo C, Hardy D, McLeod KL, Samhouri JF, et al. (2012) An index to assess the health and benefits of the global ocean. Nature 488: 615-620.
