# Supplementary material for: Biotic and Human Vulnerability to Projected Changes in Ocean Biogeochemistry over the 21st Century
Source: PLoS Biol. 2013 Oct 15;11(10):e1001682. doi: 10.1371/journal.pbio.1001682 (PMC3797030; doi:10.1371/journal.pbio.1001682)

# Supplement 1. Standardization of grid resolutions among models.

As part of the CMIP5, modeling groups were given the freedom to choose the grids that best fitted their needs. As a result there is a considerable variation in the resolutions of the grids used (see Fig. S1). However, for the purpose of comparison among models, we used a unique grid system of one by one degree. To ensure the standardization of all databases on the same grid-resolution, we used a linear interpolation based on the results of the test below. When necessary for the purpose of ensuring equal area, we used a cylindrical equal area projection with a cylinder secant at the 30° parallel (the so-call Behrmann Projection).

*Effect of interpolation method*

We tested common interpolation methods and to evaluate the error they add, we carried out the following approach. First, we took a high resolution database (here we used the data from the model MPI-ESM-MR, which for the most part is provided at ½ degree) and used two physical-chemical parameters with opposite patterns of spatial homogeneity [i.e. surface temperature (page S30) and particulate organic carbon flux (page 197)]. From these two databases, we removed every other point and used the resulting database for interpolation with the different methods and the removed points as a test. The magnitude of the error introduced by each interpolation method was assessed by the level of correlation between their interpolated values at the position of the test points and the original value of the test points. Description and results of the different interpolation methods are provided below.

Nearest Neighbor

This method produces a Thiessen polygon for each sample point, such that all points are nearer to its enclosed sample point than to any other sample points. The value for the un-sampled cell is determined by the nearest cell center on the Thiessen grid [[1](#_ENREF_1)].

Linear

With this method, the value at each un-sampled point is calculated from a linear regression model applied to the vertices of the Delaunay triangulation simplex (or triangle) into which the un-sampled point falls [[1](#_ENREF_1)].

Natural Neighbour

This method is similar to the Nearest Neighbor method with the difference that for each un-sampled point, the areas of contributing Thiessen polygons are calculated, scaled to sum to 1 and used as weights for the corresponding samples [[1](#_ENREF_1)].

Inverse Distance Weighted Interpolation

This method estimates the values at unsampled points using a linear combination of the values at, and distance to, sampled points. The assumption is that sampled points closer to the unsampled point are more similar to it than those further away. The main factors affecting the accuracy of IDW are 1) the choice of the power parameter, which often takes values of zero, one and two, with the resulting method often called “moving average”, “linear interpolation” and “inverse distance squared”, respectively; and 2) the number of neighboring points, which takes values of 4, 6, and 8 in our experiments [[1](#_ENREF_1)].

Thin-plate

This method consists of polynomials that fit a given number of sampled points exactly generating a surface of values over the entire area to be interpolated. The smoothing parameter is chosen in an ad hoc fashion.

Kriging

Kriging fits a function to a specific number of points or all points within a radius to determine the output value for each location, with the fitted curve not necessary passing exactly through the sampled points. Here, we fitted the experiment variograms using a spherical model and performed interpolation using ordinary kriging.

References

1. Li J, Heap AD (2008) A Review of Spatial Interpolation Methods for Environmental Scientists. Geoscience Australia.

**Test on temperature data**


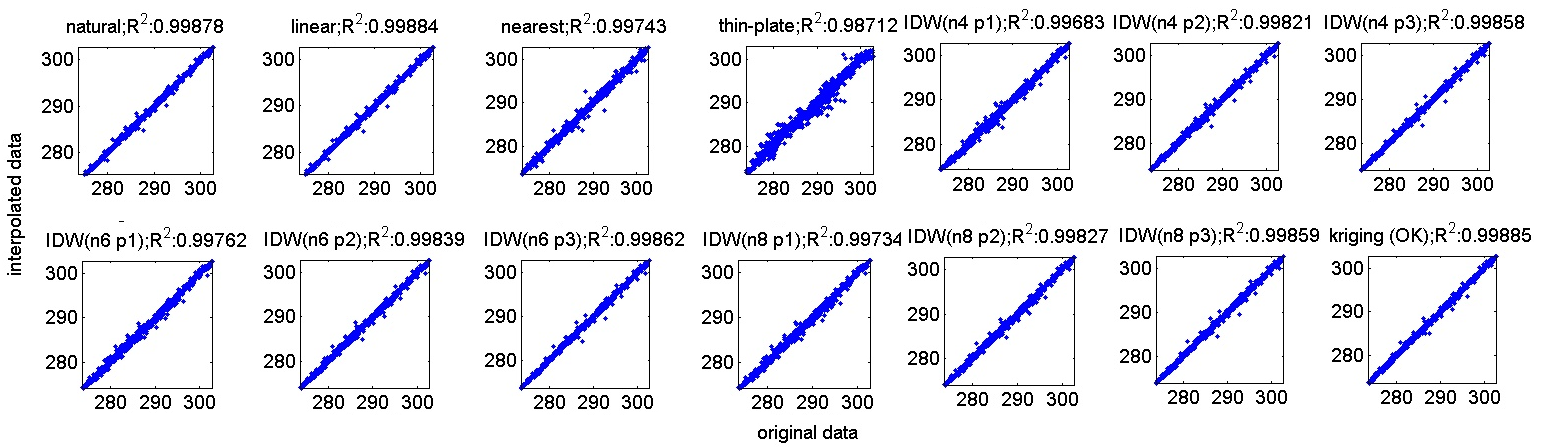


**Test on Particulate Organic Carbon Flux**
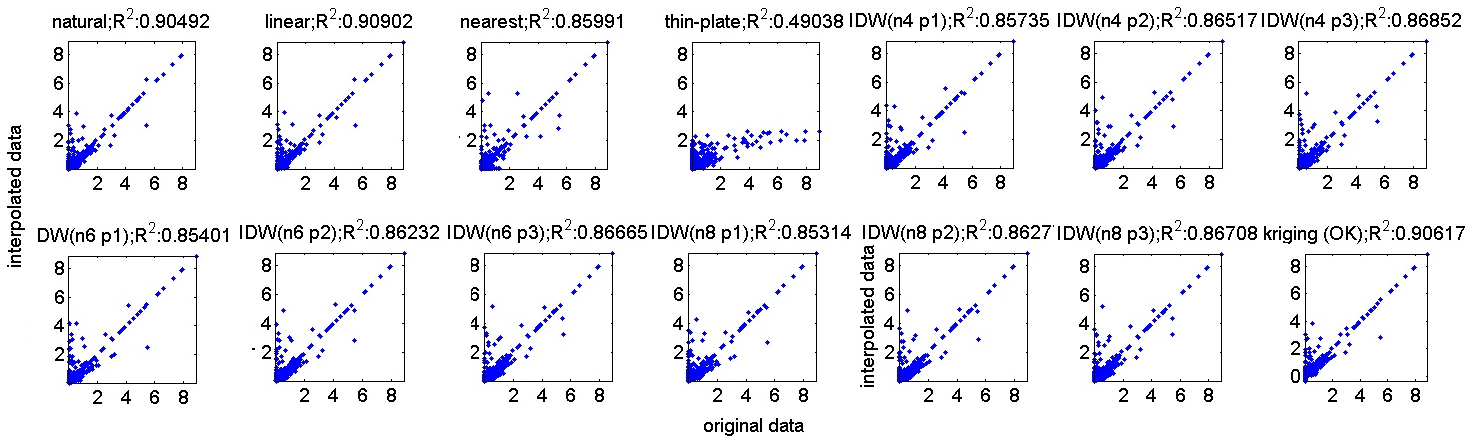

Supplement: Supplement S1 — Standardization of grid resolutions among models. (DOCX) [file pbio.1001682.s009.docx]
