# Supplementary material for: Biotic and Human Vulnerability to Projected Changes in Ocean Biogeochemistry over the 21st Century
Source: PLoS Biol. 2013 Oct 15;11(10):e1001682. doi: 10.1371/journal.pbio.1001682 (PMC3797030; doi:10.1371/journal.pbio.1001682)
Supplement: Supplement S2 — R scripts for processing CMIP5 files. (DOCX) [file pbio.1001682.s010.docx]

Supplement 2. R scripts for processing CMIP5 files. Available data in the CMIP5 represent the current state of affairs regarding climate change research [[1](#_ENREF_1),[2](#_ENREF_2)]. However, the number and size of the files impose a considerable impediment to personal computers and thus to the overall use of the data by non-expert users, particularly when data of all models are to be considered, which is highly recommended given that multimodel averages are commonly more accurate that models individually (Table. S1-S2). As an example, data from any given model for a given parameter often consist of 346 years (i.e. 156 years from 1850 to 2005 for the historical experiment, and 190 years from 2006 to 2100 for the two RCP experiments considered) at ~50 depths (from 0 meters to the seafloor) over the entire ocean (the spatial resolution varies by model, Table S1). In total, the number of files analyzed in this study combining all models, parameters and years was 27,792. Obtaining the actual values for a given parameter for a single year at the surface and the seafloor from any of these files, using ArcGis tools, takes on average two minutes. Thus processing the entire data analyzed here takes on the order of 40 days using a computer with an I7 processor at 3.3GHz. The other complication is the size of the files. The data in the CMIP5 are in “Network Common Data Form (i.e. NetCDF)” files and all the files analyzed here occupy 2.3 terabytes, ranging from files of ~10 megabytes to up to 25 gigabytes (models provided their data in files containing variable number of years; in other words, the entire time series for a given experiment could have been delivered in full in a single file or fractionated into smaller files with consecutive subsets of the years). Files with many years certainly facilitate data downloading but their large size posed an additional limitation for data processing as the entire file has to be loaded in the memory RAM of the computer making it crash if the overall use of memory RAM exceeds what is available to the computer (the most advance Intel I7 processor is limited to 16GB of memory RAM; the alternative is to use a Workstation, which allows for more memory RAM but such machine and configuration are considerably more expensive).

To overcome these limitations, we developed few short scripts in the software R and the open-access software NCO (all operational in Windows) that reduced considerably the processing time (~5 seconds per year) using a very basic computer. The NCO software is used solely to subdivide the NetCDF files into single years, which speeds up processing and prevents using considerable amount of memory RAM. Below we provide detailed instructions on the use of such scripts, which we hope will facilitate more uses of the CMIP5 data and to update our results as data from more models become available. The scripts below are based on the standards for file naming in the CMIP5 (so do not change the original CMIP5 file names).

1. Install NCO in Windows. Follow the instructions provided here: <http://sourceforge.net/projects/nco/forums/forum/9830/topic/5436391>. This is only necessary if you run into problems of memory RAM while processing CMIP5 NetCDF files.

2. In Windows, NCO runs in the “Command Prompt” also called “cmd” software; so you need to ensure that Command Prompt opens in the directory where you will be working with NCO. Here we created a folder named “WorkingFolder” located in an external hard-drive. The path to that folder is “G:\WorkingFolder”.You may have to change this path depending on where you placed your working folder.

To set Command Prompt to the working folder path, type “regedit” in the search window at the Start bottom in Windows and press Enter.


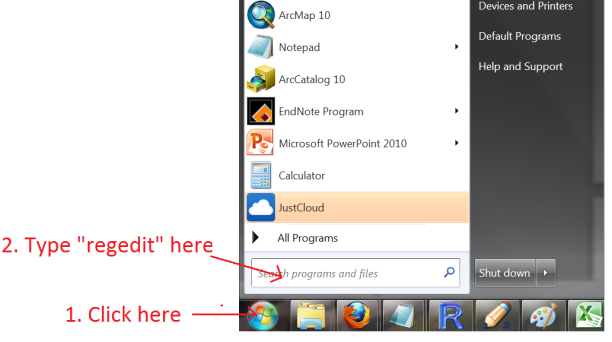


The “Registry Editor” window will pop-up. Scroll down to [HKEY_LOCAL_MACHINE\Software\Microsoft\Command Processor], click on “Command Processor” and double click on Autorun in the right-hand window. Type “CD /d G:\WorkingFolder” in the appearing window and then click ok. Change the “G:\WorkingFolder” part according to the path where you put your working folder.


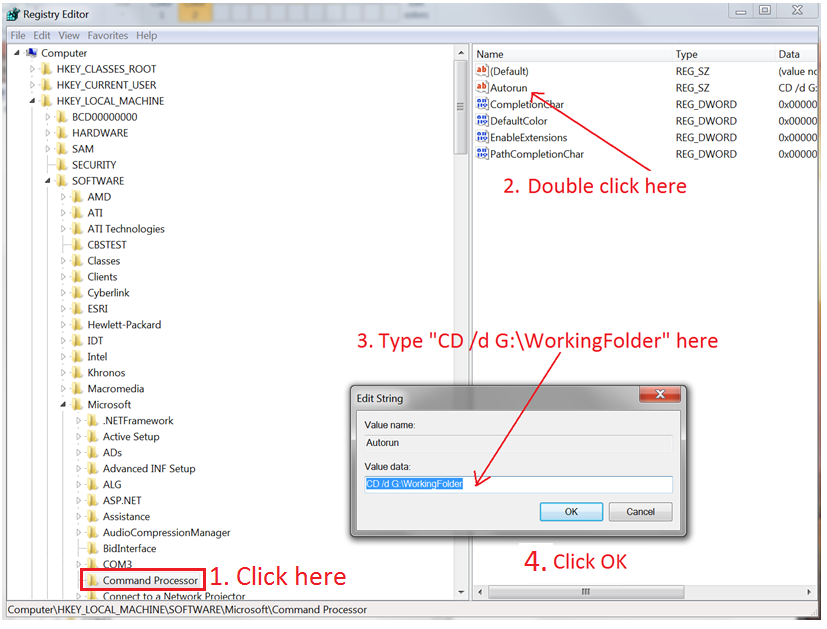


You are now set to use NCO in your working folder.

**Generating single year NetCDF files**

As mentioned above this step is implemented to reduce the size of the files, which speeds up processing and prevents using too much memory RAM. This step was also implemented to average monthly data, available only for temperature in some models, into a single year data file. The routine below simply uses a shell, in which R sets the commands used by NCO to generate the year-specific-NetCDF file. All of the tools available in the NCO software are stand-alone and here we use the “ncra” tool, which generates new NetCDF files by averaging the time series from an existing multi-year NetCDF file. It should be noted that the commands for “ncra” have to be entered manually in Command Prompt, which is unpractical and error prone when there are thousands of files to be processed. So the script below takes advantage of R programming to run NCO from within R and generate single year data from all of the multi-year NetCDF files contained in your WorkingFolder.

| *#****R-Script to generate single year NetCDF files from Multi-year CMIP5 NetCDF files using NCO-software.*** *Running this script is optional depending on whether you have a computer with enough memory to handle some of the large files available for some models in the CMIP5. By Camilo Mora, Ph.D., cmora@hawaii.edu*  *#Place all your multi-year NetCDF files obtained from the CMIP5 web-page in your “WorkingFolder”. Do not change the names of the original files. From the folder where you installed NCO, copy the file “ncra” and paste it in the “WorkingFolder”. Then just copy the script below, paste it in R and press Enter. The single year NetCDF files will be automatically generated in your WorkingFolder.*  path = "G:\\WorkingFolder\\" *#Defines the path to your working directory. Change this path accordingly*  Files =list.files (path) *#Gets the names of all the NetCDF files in your WorkingFolder*  Files=Files[Files!="ncra.exe"] *#Exclude the file “ncra” from your list of NetCDF files*  nFiles=length(Files) *#Counts the number of files to be processed*  for (i in 1:nFiles) *#Runs the procedure below for each NetCDF file in the working folder*  {  *#Gathers all the information to name the output file*  Variable=unlist(strsplit(Files[i], "_"))[1]  Model=unlist(strsplit(Files[i], "_"))[3]  Year1=as.numeric(substring(unlist(strsplit(Files[i], "_"))[6],1,4))  Year2=as.numeric(substring(unlist(strsplit(Files[i], "_"))[6],6,9))  Experiment=unlist(strsplit(Files[i], "_"))[4]  nTime= Year2- Year1 + 1  for (year in 1:nTime) *#Creates a NetCDF file for each year in the original multi-year NetCDF file.*  {  *#These are the commands to be run in ncra. Basically, this script opens Command Prompt, pastes the commands for ncra to calculate the average for the given year, saves the file as a new NetCDF file and then closes Command Prompt.*  shell(paste("ncra -F -d time,",year,",",year," ",Files[i]," ",Variable,"_Oyr_",Model,"_",Experiment,"_r1_",Year1+year-1,"-",Year1+year-1,".nc",sep=""),"C:/Windows/System32/cmd.exe",wait = TRUE)  }  }  *#All generated yearly files will now be located in your WorkingFolder. The names for the new files will maintain the same configuration as the original CMIP5 files.*  *# You will likely get some warnings in R, which are related to the internal configuration of ncra for Windows: (http://sourceforge.net/projects/nco/forums/forum/9830/topic/5511826). This warning, however, do not affect the data.* |
| --- |

**Extracting values from NetCDF files at the sea surface and floor**

This script is intended to generate summary files for the ocean surface and seafloor data that can be opened in Excel and most common statistical packages. Simplistically, the script takes all the NetCDF files that you place on a working folder and for each one year contained in the NetCDF files it generates a CSV file containing the values at the ocean surface and seafloor. To use this code:

1. Generate a new folder (name this “WorkingFolder”). Copy the path to this folder and replace it in the code below (Highlighted in red).

2. Place all your NetCDF files obtained from CMIP5 web-page in the folder created above.

3. Generate a new folder (name this “OutputFolder”). Copy the path to this folder and replace it in the code below (Highlighted in orange). This is the folder where R will save the generated files.

4. Copy the code in the grey box below, paste it in R and wait for the files to be generated.

NOTE. The results of this script have been cross-check with the results for the same NetCDF files processed with ArcGIS. The script is self-adaptable to several differences that remain among the NetCDF files provided by the different modeling centers to the CMIP5. We tested the script with all models considered in Table S1. Specific description for each step of the script is provided below.

| *#****R-Script to extract the values for the ocean surface and seafloor from CMIP5 NetCDF files****. This script generates csv files that can be opened in Excel or any other statistical program. The resulting files contain the latitude, longitude and values for each cell at the surface and seafloor. The generate files do maintain the generic naming of the original file. This script will crash if the memory RAM used exceeds what is available in your computer. If you face this problem, you can buy more RAM or fraction the NetCDF files into single years using the script provided above for NCO. By Camilo Mora, Ph.D., cmora@hawaii.edu*  path = "**G:\\WorkingFolder\\**" *#Path to the working folder. Change accordingly to the path of your Workingfolder.*  setwd("**G:\\OutputFolder\\**") *#Path to the folder where R will place the CSV files. Change accordingly to the path of your OutputFolder.*  library(reshape) *#Loads this package. If not installed, click on “Packages” in R, click on Install packages and follow instructions*  library(ncdf) *#Loads this package. If not installed, click on “Packages” in R, click on Install packages and follow instructions*  Files =list.files (path) *#Gets the names of all the NetCDF files in your WorkingFolder*  nFiles=length(Files) *#Counts the number of files to be processed*  for (i in 1:nFiles) *#For each NetCDF file it extracts the values of the parameter at the ocean surface and seafloor*  {  nc=open.ncdf(paste(path,Files[i],sep = "")) *#Opens NetCDF file* ***i*** *for processing*  *#Gathers all the information to name the output CSV file*  Variable=unlist(strsplit(Files[i], "_"))[1] *#Obtains the name of the variable/parameter of the file*  Model=unlist(strsplit(Files[i], "_"))[3] *#Obtains the name of the model of the file*  Year1=as.numeric(unlist(strsplit(unlist(strsplit(Files[i], "_"))[6], "-"))[1]) *#Obtains the first year in the file*  Experiment=unlist(strsplit(Files[i], "_"))[4] *#Obtains the name of the experiment in the file*  *#Gathers internal information that NetCDF file used to name the different dimensions*  dLon=nc$var[[Variable]]$dim[[1]]$name *#Obtains name used in the NetCDF file to refer to longitude. This name varied by model*  dLat=nc$var[[Variable]]$dim[[2]]$name *#Obtains name used in the NetCDF file to refer to latitude. This name varied by model*  Depth=nc$var[[Variable]]$dim[[3]]$name *#Obtains name used in the NetCDF file to refer to depth. This name varied by model*  Time ="time" *#All models named the dimension containing the years as “time”*  *#Values in each dimension*  vTime = get.var.ncdf(nc, Time)  vLon = get.var.ncdf(nc, dLon)  vLat = get.var.ncdf(nc, dLat)  vDepth = get.var.ncdf(nc, Depth)  vDepthOrdered = vDepth [order(vDepth)]  *#Number of values in each dimension*  nTime=length(vTime)  nDepth=length(vDepth)  nLon=length(vLon)  nLat=length(vLat)  *#Creates an empty data frame with the index coordinates for each cell. Values of the cells at each depth will be appended to this frame*  DataFrame=get.var.ncdf(nc, Variable, start=c(1,1,1,1), count=c(nLon,nLat,1,1))  DataFrame=as.data.frame(melt(DataFrame)[,1:2])  *#For each year in the NetCDF file* ***i*** *it extracts the values of the parameter for each cell at the ocean surface and seafloor at time* ***a***  for (a in 1:nTime)  {  for (b in 1:nDepth) *#Generates a matrix with the values for the given year* ***a*** *of all cells at all depths*  {  c=which(vDepth == vDepthOrdered[b]) *#Gets the dimension position of the given depth from the shallowest to the deepest.*  *#Generates a matrix with all the values for the given depth* ***b*** *at each coordinate at the time* ***a****.*  Data = get.var.ncdf(nc, Variable, start=c(1,1,c,a), count=c(nLon,nLat,1,1))  DatabyColumns=as.data.frame(melt(Data)[,3]) *#Converts matrix to flat table*  *#In some models land was represented as 1e20. This value is replaced with NA for consistency with most models.*  DatabyColumns=replace(DatabyColumns,DatabyColumns>1000000,NA)  colnames(DatabyColumns)=round(vDepthOrdered[b],0) *#Names the generated column with the value of the given depth* ***b***  *#Add the data from depth* ***b*** *to the data frame with all coordinates*  DataFrame=cbind(DataFrame,DatabyColumns)  }  #DataFrame[DataFrame==0]<-NA *#Note: one model (i.e. Thetao in MRI-CGCM3) set land to zero. In that case you have to active this script, which sets land to NA*  *#Extract data for the surface and seafloor*  dat2<-data.frame(t(DataFrame[,3:nDepth]))  dat3<-lapply(dat2,function(x) tail(x[!is.na(x)],1))  dat4<-ifelse(sapply(dat3,length)==0,NA,dat3)  dat5<-data.frame(unlist(dat4))  dat6<-data.frame(DataFrame[1:3],dat5)  *#Final table with matrix coordinates and values for the surface and seafloor*  colnames(dat6)=c("x", "y", "Surface", "Seafloor")  *#This section adds the latitude and longitude coordinates for each cell and centers the coordinates between -180 and 180. One important difference among the NetCDF files provided by the different modeling centers is that some centers provided latitudes and longitudes as a separate dimension, whereas others provided only the unique values of latitudes and longitudes. The code below adjust for such as difference among NetCDF files*  *#Determines if Latitude was provided as an independent dimension. The result for Longitude is the same.*  if(is.null(nc$var[["lat"]]$ndims)==TRUE){  dLat=data.frame(melt(nc$var[[Variable]]$dim[[2]]$vals))  dLon=data.frame(melt(nc$var[[Variable]]$dim[[1]]$vals))  dLon=data.frame(dLon[1], value=ifelse(dLon$value<=180, dLon$value, dLon$value-360))  dLon=data.frame(dLon[1], value=ifelse(dLon$value>-180, dLon$value,360+ dLon$value)) *#Center all longitudes between -180 to 180*  dt7=merge(dat6,dLon,by.x="x",by.y="indices")  dt8=merge(dt7,dLat,by.x="y",by.y="indices")  colnames(dt8)=c("y", "x", "Surface", "Seafloor", "LONGUITUDE", "LATITUDE")  } else  {  dLat= data.frame(melt(nc$var[[Variable]]$dim[[2]]$vals))  dLon=data.frame(melt(nc$var[[Variable]]$dim[[1]]$vals))  dLon=data.frame(dLon[1], value=ifelse(dLon$value>-180, dLon$value,360+ dLon$value)) *#Center all longitudes between -180 to 180*  dt7=merge(dat6,dLon,by.x="x",by.y="indices")  dt8=merge(dt7,dLat,by.x="y",by.y="indices")  colnames(dt8)=c("y", "x", "Surface", "Seafloor", "LONGUITUDE", "LATITUDE")  }  *#Saves the results keeping the original NetCDF file name. If you want the data from all depths, replace “dt8” for “DataFrame” in the script below*  write.table(dt8, file = paste(Variable,"_",Model,"_",Experiment,"_",Year1+a-1,".csv", sep = ""), sep = ",", col.names = NA)  }  }  proc.time() *#calculates the time it took for data processing* |
| --- |

# References

1. Taylor KE, Stouffer RJ, Meehl GA (2011) An overview of CMIP5 and the experiment design. Bulletin of the American Meteorological Society 93: 485-498.

2. Vuuren DP, Edmonds JA, Kainuma M, Riahi K, Thomson AM, et al. (2011) The representative concentration pathways: an overview. Climatic Change 109: 5-31.
